# Characterization of the Chicken‐Derived *Riemerella anatipestifer* Strains in Beijing and Fujian Provinces, China

**DOI:** 10.1155/tbed/5917276

**Published:** 2026-09-16

**Authors:** Pengfei Niu, Rong Guo, Yixuan Li, Yingting Cui, Fan Sun, Changming Guo, Qi Feng, Shanyuan Zhu, Shengqing Yu

**Affiliations:** ^1^ College of Animal Sciences, Fujian Agriculture and Forestry University, Fuzhou 350002, China, fjau.edu.cn; ^2^ Shanghai Veterinary Research Institute, Chinese Academy of Agricultural Sciences (CAAS), Shanghai 200241, China, caas.cn; ^3^ Jiangsu Key Laboratory for High-Tech Research and Development of Veterinary Biopharmaceuticals, Jiangsu Agri-Animal Husbandry Vocational College, Taizhou 225309, China, jsahvc.edu.cn; ^4^ Yangzhou You-Jia-Chuang Biotechnology Co., Ltd., Yangzhou 225261, China

**Keywords:** chicken, genomic analysis, pathogenicity, *R. anatipestifer*, virulence genes

## Abstract

*Riemerella anatipestifer* is an infectious pathogen that mainly affects waterfowl such as ducks and geese. Recently, it is becoming increasingly prevalent in chicken flocks in China. In this study, we isolated 12 strains of *R. anatipestifer* from chickens with clinical signs in Beijing and Fujian Provinces, China. Serotyping indicated five strains from Fujian Province belonged to serotype 1, two strains from Beijing belonged to serotype 10, and five strains from Fujian Province were not assigned to serotypes 1–13. All the 12 strains exhibited high rates of resistance to amikacin, gentamicin, kanamycin, norfloxacin, and polymyxin B and low virulence to healthy chicks and ducklings. Chicks and ducklings in experimental infection groups showed temporary depression and decreased appetite within 2 days after infection with any of the 12 strains. Furthermore, we selected RA‐FJ‐C1 (serotype 1), RA‐FJ‐C4 (untypable by serotypes 1–13 primers), and RA‐BJ‐C1 (serotype 10) strains for whole genome sequencing and predicted genes related to virulence, antimicrobial resistance, and pathogen‐host interactions. Comparative genomic analysis demonstrated that RA‐FJ‐C1, RA‐FJ‐C4, and RA‐BJ‐C1 harbored 399, 151, and 149 strain‐specific genes, as well as 10, 9, and 1 unique virulence genes, respectively. There were seven and eight strain‐specific virulence genes in serotype 1 strains CH3 and RA‐FJ‐C1, respectively, whereas nine and 15 specific virulence genes were identified in serotype 10 strains HXb2 and RA‐BJ‐C1. Moreover, most of these virulence genes were associated with capsules, lipopolysaccharide (LPS), or lipooligosaccharide (LOS). Colinearity analysis revealed that the gene arrangements of the genomes RA‐FJ‐C1, RA‐FJ‐C4, and RA‐BJ‐C1 were more similar to those of the Yb2 and JN1 strains, while it differed significantly from that of S63 and HXb2 strains. These findings provide a supplement to the limited study of *R. anatipestifer* from chicken; enhance our understanding of its prevalent serotype, drug resistance, and pathogenicity; and provide scientific foundation for the treatment and control of the disease.

## 1. Introduction

*Riemerella anatipestifer* is a Gram‐negative pathogen that causes acute and contagious infectious diseases in ducks, geese, turkeys, and a variety of domestic and wild birds [1]. *R. anatipestifer* infection can be acute or chronic, with acute infection being more common in ducks younger than 8 weeks of age [2]. Clinical signs include diarrhea, lethargy, ocular and nasal secretions, respiratory symptoms (such as dyspnea, coughing, and sneezing), nervous symptoms (such as tremors of the head and neck and ataxia), and pathological lesions present as fibrinous pericarditis, perihepatitis, airsacculitis, and meningitis [2–5]. The resulting high incidence and mortality rates have thus caused significant economic losses to the global poultry industry [6]. In recent years, reports have shown an increase in the number of cases of *R. anatipestifer* infection in chickens, including broiler chickens, laying hens, and breeder chickens [7, 8]. Chickens infected with *R. anatipestifer* exhibit a range of clinical signs, such as coughing, sneezing, and neurological symptoms. Postmortem examination reveals lesions including airsacculitis, perihepatitis, and pericarditis. The infected breeding flocks reduce egg productivity and hatching rates and cause jelly‐like lifeless embryos [8]. Broilers over 30 days often develop leg diseases, featuring joint swelling or the presence of purulent exudates in the joint cavity [9]. Laying hens often present with salpingitis. Postmortem examination reveals caseous material accompanied by milky white serous fluid in the oviduct, and this pathological change causes higher mortality rates among early‐stage embryos, inability to achieve peak egg production, and inferior eggshell quality [9]. In this study, we collected a total of 77 clinical samples from one breeder farm and eight broiler farms. The breeder chickens presented mild respiratory signs, accompanied by decreased egg production and poor eggshell quality. In broilers, clinical signs were dominated by respiratory symptoms, including moist cough and slight rales. Some affected chickens also exhibited depression and swollen joints. Then, we isolated 12 *R. anatipestifer* strains from these chickens with clinical symptoms. We further performed serotype identification, antibiotic resistance examination, and pathogenicity assessments of the 12 strains. Moreover, we selected three strains for whole genome sequencing and characterization. These findings will provide a scientific guidance for the clinical treatment of *R. anatipestifer* infections in chickens.

## 2. Materials and Methods

### 2.1. Bacterial Isolation and DNA Extraction

In this study, 77 samples of hearts, livers, brains, and tarsal joint tissues were collected from chickens from nine different farms in Beijing and Fujian Provinces, China, for bacterial isolation and DNA extraction. The tissue samples were streak‐inoculated on tryptic soy agar (TSA) and then incubated at 37°C with 5% CO_2_ for 36–48 h. The suspected colonies of *R. anatipestifer* were transferred to tryptic soy broth (TSB) for subculture and then detected by polymerase chain reaction (PCR) using *R. anatipestifer*–specific primers (RA‐16S‐F and RA‐16S‐R) (Table S1) [10]. The amplification comprised an initial 4 min step at 94°C, followed by 30 cycles of 94°C for 30 s, 55°C for 30 s, and 72°C for 40 s, with a final extension step at 72°C for 5 min. DNA was extracted from tissues collected from the farm individually using a genomic DNA kit (Tiangen Biotech, Co., Ltd., Beijing, China), and an equal quantity of each DNA extract from the same farm was subsequently pooled together, resulting in the preparation of nine mixed DNA samples. Each pooled DNA sample contained all tissues collected from one farm. The DNA pool for farm 1 had 5 individual samples, pools for farms 2–8 each comprised 10 samples, and the pool for farm 9 included two samples. All these pooled samples were preliminary screened for the presence of *R. anatipestifer* by PCR.

### 2.2. Identification of the *R. anatipestifer* Strains

The isolates were identified as *R. anatipestifer* strains by PCR‐based 16S rDNA amplification using primers (16S‐27‐F and 16S‐1492‐R), followed by sequencing [11]. The amplification included an initial at 94°C for 3 min, followed by 35 cycles of 94°C for 30 s, 52°C for 30 s, and 72°C for 90 s, and a final extension at 72°C for 5 min. The isolates were further subjected to Gram staining.

### 2.3. Serotyping of the *R. anatipestifer* Strains

*R. anatipestifer* isolates were subjected to PCR‐based serotyping to determine the serotypes, using the specific primers for serotypes 1–13 (Table S1) [12]. In short, if an amplification product is generated, the serotype corresponding to the specific primer is the serotype of the isolated strain. If there is no amplification band, the isolated strain will not belong to serotypes 1–13. In addition, we used slide agglutination to verify the serotypes of the 12 isolates. The rabbit antisera against serotypes 1, 2, and 10 were developed and stored by Shanghai Veterinary Research Institute, Chinese Academy of Agricultural Sciences, China.

### 2.4. Antimicrobial Susceptibility Testing

The Kirby–Bauer method was used for antimicrobial susceptibility testing according to the Clinical and Laboratory Standards Institute [13]. Eighteen antibiotic disks (Hangzhou Microbial Reagent Co., Ltd., Hangzhou, China) were selected for testing. They included penicillins (10 U), ampicillin (10 μg), piperacillin (100 μg), cefazolin (30 μg), cefuroxime (30 μg), ceftazidime (30 μg), ceftriaxone (30 μg), cefoperazone (75 μg), amikacin (30 μg), gentamicin (10 μg), kanamycin (30 μg), tetracycline (30 μg), doxycycline (10 μg), minocycline (30 μg), erythromycin (15 μg), norfloxacin (10 μg), trimethoprim‐sulfamethoxazole (1.25/23.75 μg), and chloramphenicol (30 μg). The susceptibility of the isolates to polymyxin B was judged with minimum inhibitory concentration (MIC), which was determined by the broth microdilution method as described [14]. *Escherichia coli* ATCC 25922 was used as a quality control strain. Currently, there are no species‐specific clinical breakpoints established for *R. anatipestifer* in CLSI. The diameter of the inhibition zone was measured, recorded, and interpreted according to the published paper [15].

### 2.5. Experimental Infection of Chicks and Ducklings and Sampling

One‐day‐old Suqin yellow chicks were purchased from the Poultry Institute, Chinese Academy of Agricultural Sciences (Yangzhou, China). One‐day‐old Cherry Valley ducks were obtained from Jiangsu Lijia Poultry Co., Ltd. (Taizhou, China). Chicks and ducklings were healthy, of mixed gender, have not been vaccinated, and have been confirmed to be negative for maternal antibodies against *R. anatipestifer*. The housing conditions were maintained at a constant temperature of 30–35°C, and clean drinking water and standard feed were provided ad libitum. Animals were randomly divided into different groups using a random number table, and a blind design was adopted throughout the entire animal experiments. Animals with severe depression, continuous weight loss, and obvious clinical deterioration were judged to reach the humane endpoint and were humanely euthanized immediately to reduce unnecessary suffering. At the end of the experiment, all surviving animals were humanely euthanized via intravenous injection of pentobarbital sodium.

One hundred and eleven 18‐day‐old chicks were randomly divided into 37 groups, including 12 mixed‐route experimental groups, 12 single route of intramuscular injection groups, 12 single route of air sac injection groups, and one PBS control group, with three chicks in each group. The pathogenicity of the 12 strains to the chicks was tested. The strains used for infection were passaged three times and were in the logarithmic growth phase. The chicks in groups 1–12 were infected with one of the 12 strains at a dose of 10^8^ CFU in 400 μL PBS via intramuscular injection (200 µL) and air sac (200 µL) injection. The chicks in groups 13–24 were infected with one of the 12 strains at a dose of 5 × 10^7^ CFU via intramuscular injection (200 µL). The chicks in groups 25–36 were infected with one of the 12 strains at a dose of 5 × 10^7^ CFU via air sac (200 µL) injection. The chicks in groups 37 were injected with 400 μL PBS, as the control. Forty‐two healthy 14‐day‐old ducklings were randomly assigned to 14 groups, including 12 experimental groups, one PBS control group, and one virulent strain control group (*n* = 3 per group). The ducklings in groups 1–12 were intramuscularly injected with one of the strains at a dose of 10^8^ CFU in 500 µL PBS. Ducklings in the PBS control group were injected with 500 µL PBS. The ducklings in the virulent strain control group were intramuscularly injected with the virulent strain Yb2 (LD_50_ = 1 × 10^5^ CFU) at a dose of 10^7^ CFU in 500 µL PBS. After infection, the chicks and ducklings were observed twice a day for a period of 7 days. The clinical manifestations of the chicks and ducklings were recorded. The hearts and livers of all the experimental birds were collected for pathological observation and bacterial isolation. Throat and anal swabs were collected from the infected chicks and ducklings, and their DNAs were extracted for PCR identification using primers RA‐16S‐F and RA‐16S‐R (Table S1).

### 2.6. Determination of Bacterial Loads in Tissues of Ducklings Infected With *R. anatipestifer*

Twenty‐five 14‐day‐old ducklings were randomly divided into five groups, with five ducklings in each group, to determine bacterial loads in tissues. The ducklings in groups 1–3 were intramuscularly inoculated with 500 μL of RA‐FJ‐C1, RA‐FJ‐C4, or RA‐BJ‐C1 strains at a concentration of 2 × 10^8^ CFU/mL, respectively. The ducklings in group 4 were intramuscularly inoculated with 500 μL of PBS. The ducklings in the virulent strain control group were intramuscularly injected with 500 μL of the virulent strain Yb2 at a concentration of 2 × 10^7^ CFU/mL. On the 3rd day after inoculation, ducklings were humanly sacrificed and dissected. Liver, heart, brain, and blood samples were collected, and tissues were individually lysed. Then, the collected blood samples and lyzed tissue samples were diluted with PBS appropriately and spread on TSA plates, which were incubated at 37°C with 5% CO_2_ incubator for 36 h for bacterial counting.

### 2.7. Whole Genome Sequencing and Assembly

Three different serotypes of the strains RA‐FJ‐C1, RA‐FJ‐C4, and RA‐BJ‐C1 were selected for whole genome sequencing and assembly. The strains were cultured in TSB medium to the logarithmic growth phase and then transferred to centrifuge tubes and centrifuged at 14,000 g for 5 min at room temperature, respectively. The medium was completely discarded, and the bacterial pellet was transferred to a 1.5 mL sterile cryotube (the height of the pellet should reach about 0.5–1.0 mL or weigh 0.5–1.0 g) and immediately stored at −80°C. Genome sequencing was performed by Shanghai Majorbio Bio‐pharm Technology Co., Ltd. (Shanghai, China).

Genomic DNA was sequenced using a combination of long‐read PacBio Sequel IIe and short‐read Illumina sequencing platforms. For Illumina sequencing, DNA samples were sheared into 400–500 base pair (bp) fragments. Illumina sequencing libraries were prepared from the sheared fragments using the NEXTFLEX Rapid DNA‐Seq Kit. The prepared libraries then were used for paired‐end Illumina sequencing (2 × 150 bp) on Illumina Novaseq 6000 (Illumina Inc., San Diego, CA, USA). For PacBio sequencing, genomic DNA was fragment at ~10 kb. DNA fragments were then purified, end‐repaired, and ligated with SMRT bell sequencing adapters following manufacturer’s recommendations (Pacific Biosciences, CA). Next, the PacBio library was prepared and sequenced on one SMRT cell using standard methods. The raw Illumina sequencing reads were subjected to quality‐filtered using fastp v0.23.0. The HiFi reads were generated from the PacBio platform. Then, the clean short reads and HiFi reads were assembled using the Unicycler v0.4.8 software [16], and during the assembly process, the Pilon v1.22 software was used for sequence correction. Contamination and completeness of the genomes were assessed using CheckM v1.1.3.

### 2.8. Genome Annotation and Analysis

The coding sequence (CDS) in the genome was predicted using the software Glimmer [17] (http://ccb.jhu.edu/software/glimmer/index.shtml), GeneMarkS [18], and Prodigal [19]. The transfer RNA (tRNA) in the genome was predicted using the software tRNAscan‐SE v2.0 [20] (http://trna.ucsc.edu/software/). The Barrnap and Infernal software were utilized to predict ribosomal RNA (rRNA) and small noncoding RNA (sRNA). Databases were used to predict gene functions, including NR [21], Swiss‐Prot [22], Pfam (http://pfamxfam.org/), COG (http://www.ncbi.nlm.nih.gov/COG/), GO [23], and KEGG [24]. Genomic maps were generated using Circos software [25]. Island Viewer [26], Phage_Finder [27], Minced (https://sourceforge.net/projects/minced/), Integron_Finder_v2.0 [28], ISEScan_v1.7.2.1 (https://github.com/xiezhq/ISEScan/release), and Transposon PSI (https://transposonpsi.sourceforge.net/) software were used to analyze the gene island (GI), prophage, clustered regularly interspaced short palindromic repeats (CRISPR)/Cas, integron, insertion sequence (Is), and transposon (Tns), respectively. Drug resistance genes were predicted using the CARD database [29]. Virulence factors and pathogen‐host interaction analysis were performed by using virulence factor database (VFDB) (http://www.mgc.ac.cn/VFs/main.htm) and PHI (http://www.phi-base.org/) databases, respectively. Matches with an *E*‐value ≤ 1e–5 were retained.

### 2.9. Comparative Genomic Analysis

The strain‐specific genes of RA‐FJ‐C1, RA‐FJ‐C4, and RA‐BJ‐C1 strains were obtained via homologous gene analysis. Specifically, we performed orthologous gene analysis using OrthoMCL v2.0.9 to classify gene families and calculate the exact gene count of each sample assigned to individual gene families (*E*‐value ≤ 1e–5). Based on the analysis results, strain‐specific genes of individual strains were screened. In addition, differential virulence genes among RA‐FJ‐C1, RA‐FJ‐C4, and RA‐BJ‐C1 were screened from virulence factor analysis. To investigate the molecular mechanisms responsible for the virulence divergence between chicken‐ and duck‐derived strains, we also compared the virulence genes between serotype 1 strains RA‐FJ‐C1 and CH3 (duck‐derived strain) and between serotype 10 strains RA‐BJ‐C1 and HXb2 (duck‐derived strain).

To characterize genomic regions with rearrangements and horizontal transfers, the reference genomes of *R. anatipestifer* strains JN1 (CP186021.1), S63 (CP110126.1), HXb2 (CP011859.1), and Yb2 (CP007204.1) were downloaded from the NCBI database. Subsequently, Mauve software (version 2.3.1) was employed to align the genomes of RA‐FJ‐C1 (CM134216.1), RA‐FJ‐C4 (CM134217.1), and RA‐BJ‐C1 (CM134218.1) against the four reference genomes, and the collinearity atlas was generated [30].

## 3. Results

### 3.1. Detection and Isolation

The specific 16S rDNA of *R. anatipestifer* was PCR‐amplified from four of the nine pooled DNA samples with the products at an expected size of 843 bp (Figure S1A,B). Twelve suspected *R. anatipestifer* strains were isolated from chicken farms 5, 6, 7, and 9 including 2, 1, 7, and 2 isolates, respectively. The detailed information on the isolated *R. anatipestifer* strains is shown in Table S2. Among them, eight strains were from the heart, one strain was from the liver, and three strains were from the brain. After 24–48 h of incubation, the isolates grow well on TSA plate, forming round, convex‐shaped, slightly wet, semitransparent colonies (Figure S1C).

### 3.2. Molecular Identification of *R. anatipestifer* Isolates

**Figure 1:**
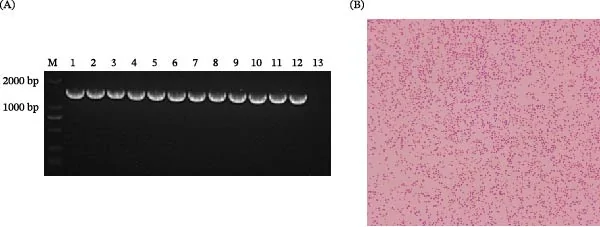
Amplification of the 16S rDNA genes and Gram staining. (A) PCR amplification of 16S rDNA gene (1465 bp fragment) from the 12 isolated strains. Lanes 1–12: strains 1–12, respectively; lane 13: negative control. (B) Gram staining of representative strain (1000×).

A 1465‐bp fragment of 16S rDNA was amplified from the 12 isolated strains (Figure 1A). They were identified as *R. anatipestifer* by further sequencing analysis. The morphology of the strains was observed under an optical microscope at 1000 × magnification. The strains were Gram‐negative stained and rod‐shaped, with rounded ends and a regular morphology. They generally exist singly and sometimes may be arranged in pairs or short chains (Figure 1B). Through 16S rDNA sequencing and Gram staining, a total of 12 strains of *R. anatipestifer* were identified and named RA‐FJ‐C1–RA‐FJ‐C10, RA‐BJ‐C1, and RA‐BJ‐C2, respectively.

### 3.3. Serotyping of the *R. anatipestifer* Isolates

Serotyping was determined by PCR and confirmed by slide agglutination test for the 12 *R. anatipestifer* strains. Of them, RA‐FJ‐C1, RA‐FJ‐C2, RA‐FJ‐C3, RA‐FJ‐C9, and RA‐FJ‐C10 (41.67%) were identified as serotype 1 strains, and RA‐BJ‐C1 and RA‐BJ‐C2 (16.67%) were serotype 10 strains. However, the serotypes of the strains RA‐FJ‐C4, RA‐FJ‐C5, RA‐FJ‐C6, RA‐FJ‐C7, and RA‐FJ‐C8 (41.67%) were untypable by the serotype 1–13 PCR scheme (Figure S2). Interestingly, serotype 1 strains RA‐FJ‐C9 and RA‐FJ‐C10 and untypable strain RA‐FJ‐C4–RA‐FJ‐C8 were isolated from one chicken farm. This phenomenon reveals the diversity and complexity of *R. anatipestifer* infections in chicken flocks.

### 3.4. Antimicrobial Susceptibility Testing

The antimicrobial susceptibility results are shown in Table 1. The 12 strains showed 100% resistance to amikacin, gentamicin, kanamycin, and polymyxin B and a high resistance rate of 91.67% to norfloxacin. Additionally, the resistance rates to erythromycin and trimethoprim‐sulfamethoxazole were 58.33%. On the other hand, they were fully sensitive to piperacillin, cefuroxime, ceftazidime, ceftriaxone, cefoperazone, doxycycline, and minocycline (100%). The susceptibility rates to ampicillin and chloramphenicol were 66.67%, and those to penicillins and cefazolin were 58.33%.

**Table 1:** The susceptibility testing of 12 *R. anatipestifer* strains to 19 antimicrobials.

Categories	Antimicrobials	Strain numbers (ratio); zone diameters (mm) or MIC (μg/mL)^a^		
		Resistance^b^	Intermediate^b^	Susceptible^b^
Penam	Penicillins	5 (41.67%); ≤14	—	7 (58.33%); ≥15
	Ampicillin	3 (25.00%); ≤13	1 (8.33%); 14–16	8 (66.67%); ≥17
	Piperacillin	0 (0.00%); ≤17	0 (0.00%); 18–21	12 (100.00%); ≥ 22
Cephalosporin	Cefazolin	3 (25.00%); ≤19	2 (16.67%); 20–22	7 (58.33%); ≥23
	Cefuroxime	0 (0.00%); ≤14	0 (0.00%); 15–17	12 (100.00%); ≥18
	Ceftazidime	0 (0.00%); ≤14	0 (0.00%); 15–17	12 (100.00%); ≥18
	Ceftriaxone	0 (0.00%); ≤13	0 (0.00%); 14–20	12 (100.00%); ≥21
	Cefoperazone	0 (0.00%); ≤15	0 (0.00%); 16–20	12 (100.00%); ≥21
Aminoglycosides	Amikacin	12 (100.00%); ≤14	0 (0.00%); 15–16	0 (0.00%); ≥17
	Gentamicin	12 (100.00%); ≤12	0 (0.00%); 13–14	0 (0.00%); ≥15
	Kanamycin	12 (100.00%;) ≤13	0 (0.00%); 14–17	0 (0.00%); ≥18
Tetracyclines	Tetracycline	6 (50.00%); ≤14	5 (41.67%); 15–18	1 (8.33%); ≥19
	Doxycycline	0 (0.00%); ≤12	0 (0.00%); 13–15	12 (100.00%); ≥16
	Minocycline	0 (0.00%); ≤14	0 (0.00%); 15–18	12 (100.00%); ≥19
Macrolides	Erythromycin	7 (58.33%); ≤13	0 (0.00%); 14–22	5 (41.67%); ≥23
Fluoroquinolones	Norfloxacin	11 (91.67%); ≤12	1 (8.33%); 13–16	0 (0.00%); ≥17
Diaminopyrimidine	Trimethoprim‐sulfamethoxazole	7 (58.33%); ≤10	0 (0.00%); 11–15	5 (41.67%); ≥16
Phenicols	Chloramphenicol	0 (0.00%); ≤12	4 (33.33%); 13–17	8 (66.67%); ≥18
Peptides	Polymyxin B	12 (100.00%); ≥4	0 (0.00%); ≤2	

^a^The MICs of polymyxin B for the isolates were determined by the two‐fold serial microdilution method, and the data were interpreted according to *Enterobacterales* [31].

^b^The data were interpreted according to [15].

### 3.5. Low Virulence to Chicks and Ducklings

In this study, all chicks inoculated with the 12 isolated strains of *R. anatipestifer* exhibited mild symptoms such as mental depression, anorexia, and a tendency to lie down. They recovered to normal 2 days postinoculation (2 dpi). On the other hand, all ducklings inoculated with the same dose of the isolated strains showed symptoms in a short period of time and recovered quickly. All the chicks in the experimental and PBS control groups did not die within 7 days. Similarly, the ducklings in the experimental groups and PBS control group did not die, whereas those in the virulent strain control group all died. On the 7th day postinfection, the chicks and ducklings in the experimental group showed no significant pathological changes in organs such as the heart and liver during the necropsy, and bacterial isolation results were all negative. Additionally, no positive result of *R. anatipestifer* 16S rDNA was detected in both oral and cloaca swabs of the chicks and ducklings. Therefore, these results showed that the 12 strains exhibited low virulence in chicks and ducklings under the tested conditions. Importantly, in the bacterial load experiment, the inoculated *R. anatipestifer* strains RA‐FJ‐C1, RA‐FJ‐C4, and RA‐BJ‐C1 were not recovered from the liver, heart, brain, or blood of the ducklings, indicating that RA‐FJ‐C1, RA‐FJ‐C4, and RA‐BJ‐C1 strains cannot survive and colonize in ducklings (Figure S3).

### 3.6. Genomic Characterization of *R. anatipestifer* Strains

**Figure 2:**
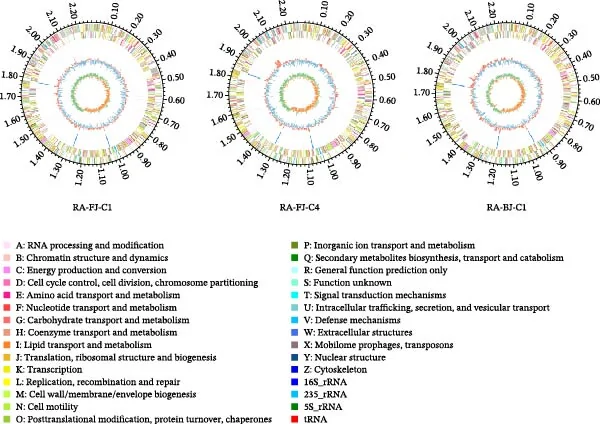
Summary of gene annotations, CDSs, and GC skew analyses of the strains RA‐FJ‐C1, RA‐FJ‐C4, and RA‐BJ‐C1. From outer to inner, the outermost circle represents the genome size; the second and third circles show the CDSs on the positive and negative strands, respectively; the fourth circle displays rRNA and tRNA; the fifth circle indicates the GC contents; and the innermost circle represents the GC‐skew values.

Genomic DNA was sequenced using a combination of PacBio Sequel IIe and Illumina sequencing platforms. Sequencing depth (PacBio HiFi) of RA‐FJ‐C1, RA‐FJ‐C4, and RA‐BJ‐C1 was 123.58×, 41.52×, and 35.13×, respectively. Coverage based on K‐mer analysis was 96.61%, 95.43%, and 98.67%. The three strains all achieved 100% genome completeness assessed by BUSCO v5.8.0 and CheckM v1.1.3. Genome maps showed that RA‐FJ‐C1, RA‐FJ‐C4, and RA‐BJ‐C1 strains each had a single circular chromosome (Figure 2). The three genomes were 2,285,028, 2,277,711, and 2,263,772 bp in size with 34.98%, 35.48%, and 35.25% G + C content and no plasmid. They contained 2139, 2142, and 2139 genes, and the average gene length is 957.52 bp, 964.33 bp, and 958.82 bp, respectively. The tRNA number of RA‐FJ‐C1 and RA‐FJ‐C4 was 40, while RA‐BJ‐C1 had 42 tRNAs. The numbers of rRNAs, 16S rRNAs, and 23S rRNAs of the three genomes were all 9, 3, and 3, respectively. The specific information of the genomes is shown in Table 2.

**Table 2:** Genomic features of *R. anatipestifer* strains RA‐FJ‐C1, RA‐FJ‐C4, and RA‐BJ‐C1.

Attributes	Strains		
	RA‐FJ‐C1	RA‐FJ‐C4	RA‐BJ‐C1
Chromosome No.	1	1	1
Plasmid No.	0	0	0
Size (bp)	2285028	2277711	2263772
G + C content (%)	34.98	35.48	35.25
CDS No.	2139	2142	2139
rRNA No.	9	9	9
tRNA No.	40	40	42
16S rRNA No.	3	3	3
sRNA No.	10	11	10
Gene island No.	5	8	6
Prophage No.	0	0	1
CRISPR No.	3	5	6
Integron No.	0	0	0
Insertion sequence No.	11	4	3
Transposon No.	2	4	4
CAZyme No.	70	63	64
Virulence gene No.	163	167	166
Antibiotic resistance gene No.	97	95	97
Pathogen‐host interaction gene No.	351	353	351
Transport protein No.	292	300	292
Transmembrane protein No.	428	429	412
Two component protein No.	25	24	22

Abbreviations: bps, base pairs; CAZymes, carbohydrate‐active enzymes; CDS, coding sequence; CRISPR, clustered regularly interspaced short palindromic repeats; No., number; rRNA, ribosomal RNA; sRNA, small noncoding RNA; tRNA, transfer RNA.

**Figure 3:**
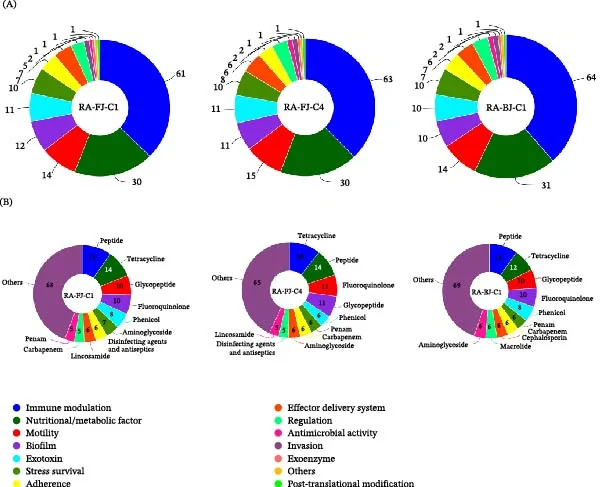
Prediction of virulence genes and resistance genes in RA‐FJ‐C1, RA‐FJ‐C4, and RA‐BJ‐C1 genomes. (A) Prediction of virulence genes. Immune modulation was the most dominant functional category, while nutritional/metabolic factors constituted the second‐largest one, with other categories harboring fewer genes. (B) Prediction of resistance genes. Genes conferring resistance to peptide, tetracycline, glycopeptide, fluoroquinolone, and phenicol antibiotics were the most prevalent. The distribution patterns of virulence and resistance genes of the three strains were largely consistent.

In this study, VFDB analysis revealed that a total of 163, 167, and 166 virulence genes were identified in the RA‐FJ‐C1, RA‐FJ‐C4, and RA‐BJ‐C1 strains, respectively (Tables S3–S5). The VFDB‐annotated virulence genes are related to immune modulation, nutritional/metabolic factor, motility, biofilm, exotoxin, stress survival, adherence, effector delivery system, regulation, invasion, posttranslational modification, antimicrobial activity, exoenzyme, and others (Figure 3A). Different types of virulence factors with different abundance were observed, and the most frequent types were immune modulation, nutritional/metabolic factor, and motility, respectively.

CARD analysis showed that RA‐FJ‐C1, RA‐FJ‐C4, and RA‐BJ‐C1 genomes harbored 97, 95, and 97 resistance genes, respectively (Tables S6–S8). At least 35 different types of antimicrobial resistance genes were identified in these three isolates, including peptides, tetracyclines, glycopeptides, fluoroquinolones, phenicols, aminoglycosides, disinfecting agents and antiseptics, lincosamides, carbapenems, penams, macrolides, cephalosporins, and others (Figure 3B). The five categories of genes associated with resistance to peptides, tetracyclines, glycopeptides, fluoroquinolones, and phenicols were the most prevalent. The resistance gene categories were predicted solely by CARD analysis, and no other AMR databases (such as ResFinder or similar databases) were used to validate the resistance gene predictions.

We compared the predicted resistance genes and detected resistance phenotypes of strains RA‐FJ‐C1, RA‐FJ‐C4, and RA‐BJ‐C1, as shown in Table S9, the resistance genotype of amikacin, gentamicin, kanamycin, norfloxacin, and polymyxin B, but not piperacillin, cefuroxime, ceftazidime, ceftriaxone, cefoperazone, doxycycline, minocycline, and chloramphenicol was consistent with their resistance phenotype in all 3 strains. The resistance genotype of penicillins, ampicillin, and cefazolin was consistent with phenotype for strain RA‐FJ‐C1, but not for strains RA‐FJ‐C4 and RA‐BJ‐C1. The genotype of trimethoprim‐sulfamethoxazol was consistent with their phenotype for strains RA‐FJ‐C1 and RA‐BJ‐C1 but not for strain RA‐FJ‐C4. The genotype of tetracycline was consistent with the phenotype for strains RA‐FJ‐C1 and RA‐FJ‐C4 but different in strain RA‐BJ‐C1.

**Figure 4:**
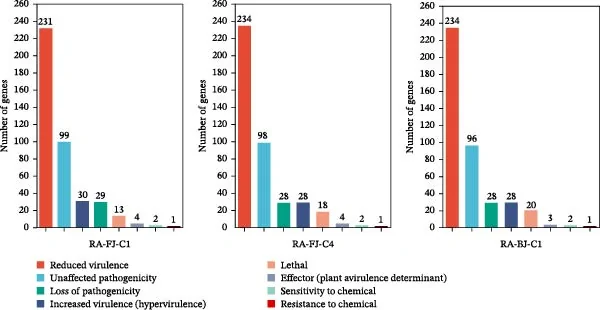
The PHI genes predicted in RA‐FJ‐C1, RA‐FJ‐C4, and RA‐BJ‐C1 genomes. Genes related to reduced virulence were the most abundant (231, 234, and 234, respectively), followed by those associated with unaffected pathogenicity (99, 98, and 96, respectively). Other categories, including loss of pathogenicity, increased virulence, lethal, effector, and chemical‐related genes, contained far fewer genes.

By comparing with the PHI database, a total of 351, 353, and 351 genes related to pathogen‐host interactions were annotated in the RA‐FJ‐C1, RA‐FJ‐C4, and RA‐BJ‐C1 genomes (Tables S10–S12). The distribution of phenotypic categories in the three strains was generally similar but with slight differences. The number of genes associated with reduced virulence was the largest, followed by genes with unaffected pathogenicity, and a smaller presence of genes related to other phenotypic categories such as loss of pathogenicity, increased virulence, lethality, and responses to chemicals (Figure 4). This distribution may provide insights into the potential mechanisms underlying the pathogenicity and survival of the isolated *R. anatipestifer* strains from chicken.

### 3.7. Comparative Genomic Analysis

By comparing the genomes of RA‐FJ‐C1, RA‐FJ‐C4, and RA‐BJ‐C1, we found that the numbers of their strain‐specific genes were 399, 151, and 149, respectively (Tables S13–S15). A large number of uncharacterized specific genes were present in RA‐FJ‐C1, RA‐FJ‐C4, and RA‐BJ‐C1 strains. In addition, the products of many genes were related to transmembrane substance transport and associated with synthesis. These specific genes may contribute to their classification into different serotypes.

Differential virulence genes were isolated from the total virulence factors of the three strains (Table 3). RA‐FJ‐C1 harbored 10 strain‐specific virulence genes, one virulence gene shared with RA‐FJ‐C4, and four virulence genes shared with RA‐BJ‐C1. RA‐FJ‐C4 had nine unique virulence genes and shared 14 virulence genes with RA‐BJ‐C1. However, RA‐BJ‐C1 possesses merely one specific virulence gene. Furthermore, we compared the virulence genes of serotypes 1 and 10 of *R. anatipestifer* strains from different hosts and found that CH3, RA‐FJ‐C1, HXb2, and RA‐BJ‐C1 harbored 7, 8, 9, and 15 specific virulence genes, respectively (Table 4). Among the virulence genes, a total of 20 genes, accounting for more than half of all genes, were associated with the virulence factors lipopolysaccharide (LPS), capsule, or lipooligosaccharide (LOS). This indicates that variations in LPS, capsule, or LOS among different strains may be key determinants of virulence differences. In addition, *FTT_RS04150*, O‐acetyltransferase A (*OatA*), *pvdI*, and *lpxD2* were only present in CH3, whereas *coxFIC1* and *groEL* are specific genes of HXb2. However, *yhbX* was identified in both CH3 and HXb2. These divergent virulence genes may collectively lead to the significant differences in virulence among these strains.

**Table 3:** Different virulence genes predicted among *R. anatipestifer* strains RA‐FJ‐C1, RA‐FJ‐C4, and RA‐BJ‐C1.

Gene ID^a^	Strains	Related genes^b^	Products^c^	Virulence factors^d^
*ACXA16_04465*	RA‐FJ‐C1	*lpxB*	Lipid A disaccharide synthetase	LPS
*ACXA16_10345*	RA‐FJ‐C1	*bepA*	Effector protein BepA	T4SS secreted effector
*ACXA16_08205*	RA‐FJ‐C1	*escN*	ATPase EscN	TTSS
*ACXA16_04530*	RA‐FJ‐C1	*ptmA*	Flagellin modification protein A	Flagella
*ACXA16_07985*	RA‐FJ‐C1	*GBS_RS06565*	Glycosyltransferase	Capsule
*ACXA16_00010*	RA‐FJ‐C1	*vpsU*	Tyrosine phosphatase VpsU	VPS
*ACXA16_04595*	RA‐FJ‐C1	*cpsD*	Glycosyltransferase, group 2 family protein	Capsule
*ACXA16_09775*	RA‐FJ‐C1	*flgR*	Response regulator	Flagella
*ACXA16_01565*	RA‐FJ‐C1	*waaV*	Glucosyltransferase	LOS
*ACXA16_04520*	RA‐FJ‐C1	*wbaP/rfbP*	Undecaprenyl‐phosphate galactosephosphotransferase	LOS
*ACXA16_07980* *ACXA17_08300*	RA‐FJ‐C1 RA‐FJ‐C4	*cpsI*	UDP‐galactopyranose mutase	Capsule
*ACXA16_10005* *ACXA18_10130*	RA‐FJ‐C1 RA‐BJ‐C1	*cap8J*	Type 8 capsular polysaccharide synthesis protein Cap8	Capsule
*ACXA16_01535* *ACXA18_01405*	RA‐FJ‐C1 RA‐BJ‐C1	*pce/cbpE*	Choline binding protein E	CBPs
*ACXA16_07995* *ACXA18_04130*	RA‐FJ‐C1 RA‐BJ‐C1	*wbtD*	Galacturonosyl transferase	LPS
*ACXA16_04640* *ACXA18_04195*	RA‐FJ‐C1 RA‐BJ‐C1	*ACICU_RS00405*	Polysaccharide biosynthesis/export family protein	Capsule
*ACXA17_04850*	RA‐FJ‐C4	*cap8O*	Type 8 capsular polysaccharide synthesis protein Cap8O	Capsule
*ACXA17_09810*	RA‐FJ‐C4	*fleR*	Two‐component response regulator	Flagella
*ACXA17_04760*	RA‐FJ‐C4	*per*	Perosamine synthetase	LPS
*ACXA17_09815*	RA‐FJ‐C4	*algB*	Two‐component response regulator AlgB	Alginate
*ACXA17_00205*	RA‐FJ‐C4	*lirB*	Dot/Icm type IV secretion system effector LirB	T4SS secreted effector
*ACXA17_01770*	RA‐FJ‐C4	*hlyB*	Hemolysin B	Hemolysin
*ACXA17_04845*	RA‐FJ‐C4	*cap8E*	Type 8 capsular polysaccharide synthesis protein Cap8E	Capsule
*ACXA17_04785*	RA‐FJ‐C4	*bplG*	Probable sugar transferase	LPS
*ACXA17_04810*	RA‐FJ‐C4	*cps4F*	Capsular polysaccharide biosynthesis protein Cps4F	Capsule
*ACXA17_06065* *ACXA18_05390*	RA‐FJ‐C4 RA‐BJ‐C1	*GBS_RS06570*	Glycosyltransferase	Capsule
*ACXA17_00010* *ACXA18_00010*	RA‐FJ‐C4 RA‐BJ‐C1	*KP1_RS17335*	Protein‐tyrosine‐phosphatase	Capsule
*ACXA17_02530* *ACXA18_01870*	RA‐FJ‐C4 RA‐BJ‐C1	*exsA*	Type III secretion system transcriptional regulator	T3SS
*ACXA17_06785* *ACXA18_07900*	RA‐FJ‐C4 RA‐BJ‐C1	*FTT_RS04145*	Glycosyltransferase family 2 protein	Capsule
*ACXA17_06935* *ACXA18_06290*	RA‐FJ‐C4 RA‐BJ‐C1	*iroC*	ABC transporter	Sal
*ACXA17_08315* *ACXA18_07885*	RA‐FJ‐C4 RA‐BJ‐C1	*KP1_RS17240*	DUF4422 domain–containing protein	LPS
*ACXA17_08300* *ACXA18_07865*	RA‐FJ‐C4 RA‐BJ‐C1	*glf*	UDP‐galactopyranose mutase	Capsule
*ACXA17_08335* *ACXA18_07910*	RA‐FJ‐C4 RA‐BJ‐C1	*lgtA*	N‐acetylglucosamine glycosyltransferase	LOS
*ACXA17_04340* *ACXA18_03685*	RA‐FJ‐C4 RA‐BJ‐C1	*mig-5*	Putative carbonic anhydrase	Mig‐5
*ACXA17_06120* *ACXA18_05445*	RA‐FJ‐C4 RA‐BJ‐C1	*fleS/flrB*	Flagellar sensor histidine kinase FleS	Flagella
*ACXA17_04855* *ACXA18_04170*	RA‐FJ‐C4 RA‐BJ‐C1	*cps4I*	Capsular polysaccharide biosynthesis protein Cps4I	Capsule
*ACXA17_06970 ACXA18_06325*	RA‐FJ‐C4 RA‐BJ‐C1	*letA*	Response regulator transcription factor	LetA (S)
*ACXA17_06785 ACXA17_08325* *ACXA18_06140 ACXA18_07900*	RA‐FJ‐C4 RA‐BJ‐C1	*FTT_RS04145*	Glycosyltransferase family 2 protein	Capsule
*ACXA18_04110*	RA‐BJ‐C1	*wbtB*	Galactosyl transferase	LPS

^a^The genes assigned in the respective genome of the three strains, corresponding to the virulence gene in the virulence factor database (VFDB).

^b^Virulence gene names in VFDB.

^c^Description of the proteins encoded by the respective virulence gene.

^d^Virulence factors associated with the respective virulence gene.

**Table 4:** Different virulence genes predicted between RA‐FJ‐C1 and CH3 and between RA‐BJ‐C1 and HXb2 strains.

Gene ID^a^	Strains	Serotypes	Related genes^b^	Products^c^	Virulence factors^d^
*M949_RS00645*	CH3	1	*FTT_RS04150*	Glycosyltransferase family 4 protein	Capsule
*M949_RS04105*	CH3	1	*lgtA*	N‐acetylglucosamine glycosyltransferase	LOS
*M949_RS11035*	CH3	1	*FTT_RS04145*	Glycosyltransferase family 2 protein	Capsule
*M949_RS04135*	CH3	1	*oatA*	Peptidoglycan O‐acetyltransferase	OatA
*M949_RS04205*	CH3	1	*yhbX*	Phosphoethanolamine transferase	LOS
*M949_RS07890*	CH3	1	*pvdI*	Peptide synthase	Pyoverdine
*M949_RS08345*	CH3	1	*lpxD2*	UDP‐3‐O‐[3‐hydroxymyristoyl] glucosamine N‐acyltransferase	LPS
*ACXA16_04320*	RA‐FJ‐C1	1	*pvdJ*	Pyoverdine	Protein PvdJ
*ACXA16_10345*	RA‐FJ‐C1	1	*bepA*	Protein BepA	T4SS secreted effector
*ACXA16_07985*	RA‐FJ‐C1	1	*GBS_RS06565*	Glycosyltransferase	Capsule
*ACXA16_01535*	RA‐FJ‐C1	1	*pce/cbpE*	Choline binding protein E	CBPs
*ACXA16_00675*	RA‐FJ‐C1	1	*wbkA*	Mannosyltransferase	LPS
*ACXA16_05980*	RA‐FJ‐C1	1	*brkB*	Serum resistance protein	Brk
*ACXA16_07980*	RA‐FJ‐C1	1	*cpsI*	UDP‐galactopyranose mutase	Capsule
*ACXA16_01565*	RA‐FJ‐C1	1	*waaV*	Glucosyltransferase	LOS
*AB406_RS00970*	HXb2	10	*coxFIC1*	Dot/Icm type IVB secretion system translocated effector	T4SS secreted effector
*AB406_RS03075*	HXb2	10	*lpxB*	Lipid A disaccharide synthetase	LPS
*AB406_RS03130*	HXb2	10	*bplG*	Sugar transferase	LPS
*AB406_RS06590*	HXb2	10	*yhbX*	Phosphoethanolamine transferase	LOS
*AB406_RS06870*	HXb2	10	*escN*	ATPase EscN	TTSS
*AB406_RS09050*	HXb2	10	*groEL*	Chaperonin GroEL	GroEL
*AB406_RS09435*	HXb2	10	*waaV*	Glucosyltransferase	LOS
*AB406_RS11185*	HXb2	10	*vpsU*	Exopolysaccharide regulatory protein VpsU	VPS
*AB406_RS11695*	HXb2	10	*bepA*	Effector protein BepA	T4SS secreted effector
*ACXA18_06140*	RA‐BJ‐C1	10	*FTT_RS04145*	Glycosyltransferase family 2 protein	Capsule
*ACXA18_04110*	RA‐BJ‐C1	10	*wbtB*	Galactosyl transferase	LPS
*ACXA18_01870*	RA‐BJ‐C1	10	*exsA*	Type III secretion system transcriptional regulator	T3SS
*ACXA18_00165*	RA‐BJ‐C1	10	*Rv2952*	Phthiotriol/phenolphthiotriol dimycocerosates methyltransferase	PDIM
*ACXA18_05390*	RA‐BJ‐C1	10	*GBS_RS06570*	Glycosyltransferase	Capsule
*ACXA18_05445*	RA‐BJ‐C1	10	*fleS/flrB*	Flagellar sensor histidine kinase FleS	Flagella
*ACXA18_07900*	RA‐BJ‐C1	10	*FTT_RS04145*	Glycosyltransferase family 2 protein	Capsule
*ACXA18_06290*	RA‐BJ‐C1	10	*iroC*	ABC transporter	Sal
*ACXA18_07885*	RA‐BJ‐C1	10	*KP1_RS17240*	DUF4422 domain–containing protein	LPS
*ACXA18_07865*	RA‐BJ‐C1	10	*glf*	UDP‐galactopyranose mutase	Capsule
*ACXA18_01765*	RA‐BJ‐C1	10	*htpB*	Heat shock protein HtpB	Hsp60
*ACXA18_06325*	RA‐BJ‐C1	10	*letA*	Response regulator transcription factor	LetA/S
*ACXA18_03685*	RA‐BJ‐C1	10	*mig-5*	Putative carbonic anhydrase	Mig‐5
*ACXA18_00010*	RA‐BJ‐C1	10	*KP1_RS17335*	Protein‐tyrosine‐phosphatase	Capsule
*ACXA18_03900*	RA‐BJ‐C1	10	*pvdJ*	Protein PvdJ	Pyoverdine

^a^The genes assigned in the respective genome of the four strains, corresponding to the virulence genes in the virulence factor database (VFDB).

^b^Virulence gene names in VFDB.

^c^Description of the proteins encoded by the respective virulence genes.

^d^Virulence factors associated with the respective virulence genes.

**Figure 5:**
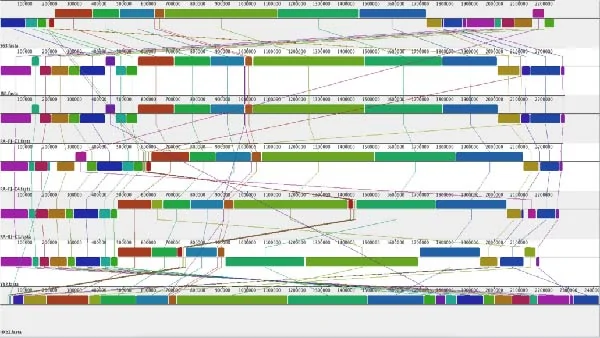
Comparison diagram of *R. anatipestifer* representative genomic sequences. Each contiguously colored region is a colinear block, a region without rearrangement of the homologous backbone sequence. Lines between genomes trace each orthologous colinear block through every genome. The gene arrangement of the strains RA‐FJ‐C1, RA‐FJ‐C4, and RA‐BJ‐C1 was highly similar to that of strains Yb2 and JN1 but differed markedly from strains S63 and HXb2. The image was generated by the Mauve rearrangement viewer.

The genomes of RA‐FJ‐C1, RA‐FJ‐C4, RA‐BJ‐C1, JN1, S63, Yb2, and HXb2 were aligned by Mauve to investigate the degree of colinearity between them. The RA‐FJ‐C1, RA‐FJ‐C4, RA‐BJ‐C1, JN1, and Yb2 genomes had consistent mosaic arrangements (Figure 5), while they differed significantly from that of S63 and HXb2 strains.

## 4. Discussion

Ducks, geese, turkeys, and other fowl are mainly susceptible to infection by *R. anatipestifer*, which is a member of the phylum Bacteroidetes that causes septicemic diseases [32]. However, the emergence of *R. anatipestifer* infection in chickens has become increasingly prevalent in recent years, demonstrating it has successfully adapted to chickens as a host [9, 11]. In this study, 77 samples were collected from chicken farms, and 12 *R. anatipestifer* strains were isolated, with a positive rate of 15.58%. While, a recent study shows that the positive rate of *R. anatipestifer* infection in chickens is 2.77% during 2021–2024 and the detection rate is 4.56% in 2024 [9]. Our results and recent reports indicate a high incidence of *R. anatipestifer* infection in chickens.

*R. anatipestifer* displays considerable diversity in its serotypes. To date, at least 21 serotypes have been identified worldwide, and studies have shown that serotypes 1, 2, and 10 are prevalent in China [33]. In this study, the serotypes of the 12 *R. anatipestifer* strains from chicken were similar to that from duck, with slight difference. While serotype 1 maintained its predominant position (41.67%), serotype 10 became a notable serotype (16.67%). However, 41.67% of the strains failed to be categorized into serotypes 1–13. The recent studies show that the *R. anatipestifer* isolates of serotypes 1 and 10 are primary, and unknown‐typeable isolates are also identified in chickens [9, 11]. Therefore, the primary distribution of serotypes 1 and 10 in chicken isolates may provide a promising foundation for the development of targeted vaccines to control and prevent *R. anatipestifer* infections in chickens.

In recent years, *R. anatipestifer* isolates exhibit a growing level of antimicrobial resistance and multiple antimicrobial resistance phenotypes in ducks [15, 34]. The recent finding shows that *R. anatipestifer* in chickens had significant resistance to amikacin, enrofloxacin, and polymyxin B [9], while our finding displayed that the 12 isolates were highly resistant to amikacin, kanamycin, gentamicin, polymyxin B, and norfloxacin. Differences in drug resistance may be related to variations in medication practices or genetic differences. In this study, we found that the resistance genotypes and phenotypes of RA‐FJ‐C1, RA‐FJ‐C4, and RA‐BJ‐C1 were not fully consistent. The discordance is likely confounded by additional factors. Resistance genes exhibit silent or regulated expression when they are not actively transcribed [35]. Furthermore, incomplete resistance gene variants do not confer resistance, such as truncated *mcr* genes [36]. Genetic or structural contexts, including the carriage of nonconjugative plasmids, disruption by Iss, or mutations in regulatory regions, may result in failure of functional expression [37]. Therefore, molecular test results should be interpreted with caution.

The 12 isolated strains had low virulence to chicks and ducklings. Infected chickens or ducks showed no obvious clinical signs, such as respiratory symptoms, and no mortality was observed. Seven days after infection, postmortem examination revealed no pathological changes in organs, and no *R. anatipestifer* DNA was detected. In the PHI analysis, genes associated with reduced virulence accounted for the majority of the distribution. Our results are highly consistent with those of the S63 strain. Chicks and ducklings infected with the *R. anatipestifer* S63 strain did not cause clinical symptoms and death, and the S63 was completely cleared from the body [8]. However, the recent study showed that chickens infected with JN01 and BZ strains exhibited obvious clinical symptoms and mortality, with typical pathological changes in the heart and liver [11].

Comparative genomic analysis showed that seven virulence genes, present in CH3 or HXb2, were absent in strains RA‐FJ‐C1, RA‐FJ‐C4, and RA‐BJ‐C1. These genes were related to diverse virulence factors, including capsules, LOS, OatA, pyoverdine, LPS, type IV secretion system (T4SS) secreted effector, and GroEL. Capsules of *R. anatipestifer* are critical for virulence, which can enhance the bacterial resistance to duck serum and enable the bacteria to cross the blood‐brain barrier [38]. LOS may contribute to the pathogenicity of *Acinetobacter baumannii* by triggering inflammatory responses through mast cells [39]. OatA plays a key role in suppressing innate immune responses and enhancing bacterial survival within the infected host [40]. During host‐pathogen interactions, iron extraction mediated by pyoverdine induces mitochondrial damage, disrupts organelle function, and initiates mitochondrial turnover via autophagic pathways [41]. LPS is not only a key virulence factor but also an important mediator of both innate and adaptive host immune responses to infection, and the LPS biosynthesis in *R. anatipestifer* is associated with the bacterial pathogenicity [42]. Effectors of T4SS manipulate host cellular pathways to promote bacterial persistence [43]. In addition, GroEL mediates environmental stress resistance in *R. anatipestifer*, which is crucial for its survival and pathogenicity [44]. So far, there have not been any studies on LOS, OatA, luciferase, or T4SS effector proteins in *R. anatipestifer*. These virulence factors are promising targets for further investigation.

In autumn and winter, large‐scale chicken houses reduce ventilation to maintain the environmental temperature. This increases the risk of coinfection with *R. anatipestifer* and other airborne pathogens. In our isolation experiment, only a very small number of *R. anatipestifer* was isolated. However, most of the isolated pathogens were *Escherichia coli*, and even *Enterococcus* and *Staphylococcus aureus* were isolated. This indicates that coinfection of *R. anatipestifer* with other pathogens exists. Interestingly, coinfections of *R. anatipestifer* with *E. coli*, H9 AIV, or IBV have also been observed in other research investigations [9, 11]. It is speculated that IBV may create conditions for the invasion of *R. anatipestifer*, while chickens infected with *R. anatipestifer* are more prone to secondary infection by other bacteria, such as *E. coli*. Consequently, the morbidity and mortality rates of the chicken flock were increased significantly. These results mean that future study on *R. anatipestifer* from chicken should focus on host immune responses, pathogenesis, and coinfection, so as to provide potential methods for the prevention and control of *R. anatipestifer* in chickens.

## 5. Conclusion

In this study, 12 *R. anatipestifer* strains were isolated. Serotyping identified five serotype 1 strains, two serotype 10 strains, and five strains untypable by the 1–13 serotyping scheme. All strains showed high resistance to amikacin, gentamicin, kanamycin, and polymyxin B and low virulence in healthy chicks and ducklings. RA‐FJ‐C1, RA‐FJ‐C4, and RA‐BJ‐C1 carried 10, nine, and one unique virulence genes, respectively. In addition, CH3 and RA‐FJ‐C1 harbored seven and eight strain‐specific virulence genes, while HXb2 and RA‐BJ‐C1 had nine and 15. Most of these genes were related to capsules, LPS, or LOS.

## Author Contributions

**Pengfei Niu:** writing – original draft, investigation. **Rong Guo**: investigation. **Yixuan Li, Yingting Cui, and Fan Sun:** methodology, data curation. **Changming Guo and Qi Feng:** resources, sampling. **Shanyuan Zhu**: project administration, funding acquisition. **Shengqing Yu**: writing – review and editing, conceptualization, supervision, funding acquisition.

## Funding

This work was supported by the Shanghai Science and Technology Innovation Action Plan (Grant 19391902800) and the Jiangsu Province Modern Agriculture Key Technology Integration and Promotion Project (Grant JCTG[2025]16).

## Disclosure

All the authors have read and approved the final manuscript.

## Ethics Statement

The protocol and the animal experiments of this study were conducted in accordance with institutional animal welfare guidelines and the ARRIVE recommendations and approved by the Jiangsu Key Laboratory for High‐Tech Research and Development of Veterinary Biopharmaceuticals, Jiangsu Agri‐Animal Husbandry Vocational College, Taizhou, China (jsahvc‐2024‐85).

## Conflicts of Interest

The authors declare no conflicts of interest.

## Supporting Information

Additional supporting information can be found online in the Supporting Information section.

## Supporting information


**Supporting Information** The supporting information for this article include Figures S1–S3 and Tables S1–S15. Figure S1: Isolation and identification of *R. anatipestifer* from chickens. Figure S2: Serotyping of *R. anatipestifer* isolates 1–12. Figure S3: Determination of bacterial loads in tissues of infected ducklings. Table S1: Primers used in this study. Table S2: Detailed information of samples and isolated strains. Tables S3–S5: Predicted virulence genes of RA‐FJ‐C1, RA‐FJ‐C4, and RA‐BJ‐C1, respectively. Tables S6–S8: Predicted resistance genes of RA‐FJ‐C1, RA‐FJ‐C4, and RA‐BJ‐C1, respectively. Table S9: Comparison of resistance genes and phenotypes in RA‐FJ‐C1, RA‐FJ‐C4, and RA‐BJ‐C1. Tables S10–S12: Predicted genes associated with pathogen‐host interactions in RA‐FJ‐C1, RA‐FJ‐C4, and RA‐BJ‐C1, respectively. Tables S13–S15: Strain‐specific genes of RA‐FJ‐C1, RA‐FJ‐C4, and RA‐BJ‐C1, respectively.

## Data Availability

The full genomic sequences of *R. anatipestifer* strains RA‐FJ‐C1, RA‐FJ‐C4, and RA‐BJ‐C1 have been submitted to the GenBank database (http://www.ncbi.nlm.nih.gov/GenBank/) under Accession Numbers CM134216.1, CM134217.1, and CM134218.1.
