# Supplementary material for: Characterization of the Chicken‐Derived Riemerella anatipestifer Strains in Beijing and Fujian Provinces, China
Source: Transbound Emerg Dis. 2026 Sep 16;2026:5917276. doi: 10.1155/tbed/5917276 (PMC13579530; doi:10.1155/tbed/5917276)
Supplement: Supplementary file 1 [file TBED-2026-5917276-s001.docx]

**Figure S1. *R. anatipestifer* strains were identified and isolated from chickens.** (A) The specific 16S rDNA (843 bp fragment) of *R. anatipestifer* was detected in DNA samples. Lanes 1–9: DNA samples from 9 different chicken farms; Lane 10: negative control. (B) The specific 16S rDNA of *R. anatipestifer* was identified in the isolates. Lanes 1–12: the 12 isolated strains; Lane 13: negative control. (C) The colonies of *R. anatipestifer* isolated strain on the TSA plate.

**Figure S2. Serotyping of *R. anatipestifer* isolates 1–12. PCR-based identification (A–H).** M: DL2,000 DNA marker; Lane 1: Samples amplified with *R. anatipestifer* specific primers; Lanes 2–14: Samples amplified with *R. anatipestifer* serotypes 1–13 primers respectively. (A–E) The strains RA-FJ-C1, RA-FJ-C2, RA-FJ-C3, RA-FJ-C9, and RA-FJ-C10, respectively, all strains were serotype 1 positive. (F–G) The strains RA-BJ-C1 and RA-BJ-C2, respectively, both strains were serotype 10 positive. (H) Strain RA-FJ-C4, representative strain of RA-FJ-C4, RA-FJ-C5, RA-FJ-C6, RA-FJ-C7, and RA-FJ-C8, unknown serotypes than serotypes 1-13. **Serum agglutination assays (I–P)**. Lane 1: Serotype 1 antiserum; Lane 2: Serotype 2 antiserum; Lane 3: Serotype 10 antiserum; Lane 4: Negative control serum. (I**–**M) The strains RA-FJ-C1, RA-FJ-C2, RA-FJ-C3, RA-FJ-C9, and RA-FJ-C10, were serotype 1, respectively. (N–O) The strains RA-BJ-C1 and RA-BJ-C2 were serotype 10. (P) Strain RA-FJ-C4, representative strain of RA-FJ-C4, RA-FJ-C5, RA-FJ-C6, RA-FJ-C7, and RA-FJ-C8, not assigned to serotypes 1–13.

**Figure S3. Determination of bacterial loads in tissues of infected ducklings.** Bacterial loads in the tissues of ducklings infected with RA-FJ-C1, RA-FJ-C4, RA-BJ-C1, Yb2, or PBS were determined on the 3rd day post-inoculation. Bacterial colony-forming units were counted.

**Table S1 The primers used in this study**

| **Primers** | **Primers sequences (5'-3')** | **Products (bp)** | **Citations** |
| --- | --- | --- | --- |
| RA-16S-F | GAGCGGTAGAGTATCTTCGGATACT | 843 | (Wu et al., 2025) |
| RA-16S-R | AATTCCTTTGAGTTTCAACCTTGCG |  |  |
| 16S-27-F | AGAGTTTGATCCTGGCTCAG | 1465 | (Zhang et al., 2025b) |
| 16S-1492-R | AAGGAGGTGATCCAGCC |  |  |
| RA-serotype 1-F | TTCAAGGAGGGGGAGGGTTA | 758 | (Zhu et al., 2024) |
| RA-serotype 1-R | TGCAACAACAGCTCCACTTG |  |  |
| RA-serotype 2-F | TCCAAAAGAGTTTGGTATTATGGGA | 614 |  |
| RA-serotype 2-R | CCAAATGCCGTACGGGAAAA |  |  |
| RA-serotype 3-F | GGAGCATTACTTTCGCAGGTC | 321 |  |
| RA-serotype 3-R | TGACGAACCCGCAGCTATTG |  |  |
| RA-serotype 4-F | TCAGCCGCAGGACTTTTTGA | 420 |  |
| RA-serotype 4-R | GGACAAACTTAATGCTCCCC |  |  |
| RA-serotype 5-F | TCGGGAATACTTGGCGCAAT | 308 |  |
| RA-serotype 5-R | GCAAAAACACCCGAGGATGC |  |  |
| RA-serotype 6-F | TCGGTGGTTTGGGGAGTTTT | 724 |  |
| RA-serotype 6-R | TCCCATATTCTGATGCCCCC |  |  |
| RA-serotype 7-F | TCCACCCCTAAGTTGGGAAC | 308 |  |
| RA-serotype 7-R | ACCCCCGACCCTCCAATAAA |  |  |
| RA-serotype 8-F | TGCGTGTCGTAACAGGGATT | 735 |  |
| RA-serotype 8-R | ACGCAATAGCTCCTCCCAAA |  |  |
| RA-serotype 9-F | GTGGTGGTTTCAGGGGATGA | 472 |  |
| RA-serotype 9-R | CTTCGCTCGCTCCATATCCA |  |  |
| RA-serotype 10-F | CAGCGGATAGGCTGTATCCAT | 522 |  |
| RA-serotype 10-R | TAGGAGCACCAATATCCCCGA |  |  |
| RA-serotype 11-F | GCAGACGCACGAAGAAGCTA | 433 |  |
| RA-serotype 11-R | TGGCATCCCATACGAAAGAACT |  |  |
| RA-serotype 12-F | TGCCCAAATACCAGTTGCCT | 208 |  |
| RA-serotype 12-R | ATCAATAGTTGCTCCCGCCC |  |  |
| RA-serotype 13-F | GGCAAGGCATGTGAGAGAGT | 634 |  |
| RA-serotype 13-R | CAACCGCTGTTGCCAAATGA |  |  |

**Table S2 Detailed information of samples and isolated strains**

| **Farms** | **Number of samples** | **Age (days)** | **Breed** | **Geographic origin** | **DNA detection** | **Strain (tissue-number)** |
| --- | --- | --- | --- | --- | --- | --- |
| 1 | 5 | 232 | Breeder | Fujian Province | Negative | None |
| 2 | 10 | 25 | Broiler | Fujian Province | Negative | None |
| 3 | 10 | 26 | Broiler | Fujian Province | Negative | None |
| 4 | 10 | 32 | Broiler | Fujian Province | Negative | None |
| 5 | 10 | 34 | Broiler | Fujian Province | Positive | RA-FJ-C1 (Heart-3),  RA-FJ-C2 (Heart-4) |
| 6 | 10 | 35 | Broiler | Fujian Province | Positive | RA-FJ-C3 (Brain-9) |
| 7 | 10 | 36 | Broiler | Fujian Province | Positive | RA-FJ-C4 (Heart-3),  RA-FJ-C5 (Heart-5),  RA-FJ-C6 (Heart-7),  RA-FJ-C7 (Heart-9),  RA-FJ-C8 (Liver-10),  RA-FJ-C9 (Brain-2),  RA-FJ-C10 (Brain-6) |
| 8 | 10 | 37 | Broiler | Fujian Province | Negative | None |
| 9 | 2 | 30 | Broiler | Beijing | Positive | RA-BJ-C1 (Heart-1),  RA-BJ-C2 (Heart-2) |

**Table S3 Predicted virulence genes of RA-FJ-C1**

| **Gene ID^a^** | **Related genes^b^** | **Products^c^** | **Virulence factors^d^** |
| --- | --- | --- | --- |
| *ACXA16_04465* | *lpxB* | Lipid A disaccharide synthetase | LPS |
| *ACXA16_01115* | *relA* | Probable GTP pyrophosphokinase RelA | RelA |
| *ACXA16_06120* | *rfbD* | dTDP-4-dehydrorhamnose reductase | LPS |
| *ACXA16_09475* | *flgJ* | Flagellar rod assembly protein/muramidase FlgJ | Flagella |
| *ACXA16_07225* | *feoB* | Fe (2+) transporter permease subunit FeoB | FupA |
| *ACXA16_04515* | *rfbC* | dTDP-4-dehydrorhamnose 3,5-epimerase | LPS |
| *ACXA16_04320* | *pvdJ* | Pyoverdine biosynthesis protein PvdJ | Pyoverdine |
| *ACXA16_04575* | *wbtH* | Asparagine synthase (glutamine-hydrolyzing) | LPS |
| *ACXA16_06165* | *cpsB/cdsA* | Phosphatidate cytidylyltransferase | Capsule |
| *ACXA16_06905* | *flhF* | Flagellar biosynthesis regulator FlhF | Flagella |
| *ACXA16_07490* | *wbtF* | NAD dependent epimerase | LPS |
| *ACXA16_04345* | *aatC* | ATP-binding protein AatC | Dispersin |
| *ACXA16_05760* | *manCcore* | Mannose-1-phosphate guanylyltransferase | LPS |
| *ACXA16_03365* | *ompA* | Outer membrane protein A | OmpA |
| *ACXA16_00375* | *LPG_RS14985* | OmpA family protein | Flagella |
| *ACXA16_00580* | *scpA/scpB* | Streptococcal C5a peptidase | C5a peptidase |
| *ACXA16_10345* | *bepA* | Bartonella effector protein BepA | T4SS secreted effector |
| *ACXA16_01500* | *acrB* | Acriflavine resistance protein B | AcrAB |
| *ACXA16_08595* | *rpoN* | RNA polymerase factor sigma-54 | Type IV pili |
| *ACXA16_09365* | *cylA* | ABC (ATP-binding cassette) transporter CylA | CylA |
| *ACXA16_08780* | *kdtB* | Lipopolysaccharide core biosynthesis protein | LPS |
| *ACXA16_08205* | *escN* | ATPase EscN | TTSS |
| *ACXA16_09820* | *bioA* | Adenosylmethionine-8-amino-7-oxononanoate aminotransferase | Biotin synthesis |
| *ACXA16_02610* | *carA* | Carbamoyl phosphate synthase small subunit | Pyrimidine biosynthesis |
| *ACXA16_04195* | *kpsU* | 3-deoxy-manno-octulosonate cytidylyltransferase | K1 capsule |
| *ACXA16_02575* | *fepD* | Ferrienterobactin ABC transporter permease | Enterobactin |
| *ACXA16_06145* | *cpsA/uppS* | Undecaprenyl diphosphate synthase | Capsule |
| *ACXA16_05600* | *YE_RS15445* | Glycosyltransferase | O-antigen |
| *ACXA16_04510* | *rffG* | dTDP-glucose 46-dehydratase | LOS |
| *ACXA16_07580* | *fepA* | Outer membrane receptor FepA | Ent |
| *ACXA16_02580* | *bauE* | Ferric siderophore ABC transporter, ATP-binding protein BauE | Acinetobactin |
| *ACXA16_10550* | *pilR* | Two-component response regulator PilR | Type IV pili |
| *ACXA16_00975* | *cesC* | ABC transporter ATP-binding protein, CesC | Cereulide |
| *ACXA16_04530* | *ptmA* | Flagellin modification protein A | Flagella |
| *ACXA16_05215* | *cylG* | 3-ketoacyl-ACP-reductase CylG | CylG |
| *ACXA16_01125* | *ggt* | Gamma-glutamyltranspeptidase | GGT |
| *ACXA16_00220* | *Rv2952* | Phthiotriol/phenolphthiotriol dimycocerosates methyltransferase | PDIM |
| *ACXA16_04660* | *pgi* | Glucose-6-phosphate isomerase | Capsule |
| *ACXA16_01930* | *htpB* | Hsp60, 60K heat shock protein HtpB | Hsp60 |
| *ACXA16_04175* | *rpe* | Ribulose-phosphate 3-epimerase | Capsule |
| *ACXA16_08900* | *fepA* | Ferrienterobactin outer membrane transporter | Enterobactin |
| *ACXA16_07370* | *recN* | DNA repair protein RecN | RecN |
| *ACXA16_10005* | *cap8J* | Type 8 capsular polysaccharide synthesis protein Cap8J | Capsule |
| *ACXA16_04505* | *wbtL* | Glucose-1-phosphate thymidylyltransferase | LPS |
| *ACXA16_09220* | *cesC* | ABC transporter ATP-binding protein, CesC | Cereulide |
| *ACXA16_00460* | *wbtG* | Glycosyl transferase family protein | LPS |
| *ACXA16_00500* | *ahpC* | Alkyl hydroperoxide reductase subunit AhpC | AhpC |
| *ACXA16_02615* | *pyrB* | Aspartate carbamoyltransferase | Pyrimidine biosynthesis |
| *ACXA16_04025* | *fadD29* | Long-chain-fatty-acid--AMP ligase FadD29 | PDIM |
| *ACXA16_08310* | *mgtB* | Mg2+ transport protein | MgtBC |
| *ACXA16_02310* | *clbF* | Colibactin biosynthesis dehydrogenase ClbF | Colibactin |
| *ACXA16_03590* | *lpxC* | UDP-3-O-(R-3-hydroxymyristoyl)-N-acetylglucosamine deacetylase | LOS |
| *ACXA16_07985* | *GBS_RS06565* | Glycosyltransferase | Capsule |
| *ACXA16_00470* | *kpsF* | D-arabinose 5-phosphate isomerase | Capsule |
| *ACXA16_06080* | *clpE* | ATP-dependent protease | ClpE |
| *ACXA16_08645* | *prsA2* | Post translocation chaperone PrsA2 | PrsA2 |
| *ACXA16_10525* | *clpP* | ATP-dependent Clp protease proteolytic subunit | ClpP |
| *ACXA16_07930* | *sodB* | Superoxide dismutase | SodB |
| *ACXA16_09810* | *bioF* | 8-amino-7-oxononanoate synthase | Biotin synthesis |
| *ACXA16_00390* | *flgS* | Signal transduction histidine kinase | Flagella |
| *ACXA16_00325* | *orfM* | Deoxyribonucleotide triphosphate pyrophosphatase | LOS |
| *ACXA16_09080* | *wbtH* | Asparagine synthase (glutamine-hydrolyzing) | LPS |
| *ACXA16_01980* | *galE* | UDP-glucose 4-epimerase | LOS |
| *ACXA16_08110* | *gluP* | Glucose/galactose transporter | LPS |
| *ACXA16_07320* | *clbG* | colibactin biosynthesis acyltransferase ClbG | Colibactin |
| *ACXA16_00610* | *AHML_RS07540* | CobQ/CobB/MinD/ParA family protein | Polar flagella |
| *ACXA16_06075* | *msrA/BpilB* | Trifunctional thioredoxin/methionine sulfoxide reductase A/B protein | MsrAB |
| *ACXA16_02390* | *tlyC* | Hemolysin C | TlyC |
| *ACXA16_01535* | *pce/cbpE* | Choline binding protein E | CBPs |
| *ACXA16_10635* | *eptC* | Phosphoethanolamine transferase | Flagella |
| *ACXA16_02980* | *bioF* | 8-amino-7-oxononanoate synthase | Biotin synthesis |
| *ACXA16_06635* | *algI* | Alginate o-acetyltransferase AlgI | Alginate |
| *ACXA16_05125* | *clpC* | Endopeptidase Clp ATP-binding chain C | ClpC |
| *ACXA16_01185* | *ybtS* | Yersiniabactin biosynthesis salicylate synthase YbtS | Ybt |
| *ACXA16_04580* | *phzH* | Phenazine-modifying enzyme | Pyocyanin |
| *ACXA16_06665* | *flmH* | Short chain dehydrogenase/reductase family oxidoreductase | Polar flagella |
| *ACXA16_04620* | *tviB* | UDP-N-acetylglucosamine C-6 dehydrogenase TviB | Capsule |
| *ACXA16_04095* | *lpxK* | Tetraacyldisaccharide 4'-kinase | LOS |
| *ACXA16_09065* | *panC* | Pantothenate synthetase | PanC/PanD |
| *ACXA16_05605* | *mucD* | Serine protease MucD precursor | Alginate |
| *ACXA16_07995* | *wbtD* | Galacturonosyl transferase | LPS |
| *ACXA16_06510* | *paa* | Outer membrane adhesin Paa | Paa |
| *ACXA16_01210* | *clbD* | Colibactin biosynthesis dehydrogenase ClbD | Colibactin |
| *ACXA16_04630* | *wbtF* | NAD dependent epimerase | LPS |
| *ACXA16_07085* | *manCcore* | Mannose-1-phosphate guanylyltransferase | LPS |
| *ACXA16_01040* | *icaA* | N-acetylglucosaminyltransferase | Intercellular adhesion proteins |
| *ACXA16_04525* | *LPG_RS03745* | Aminotransferase class I/II-fold pyridoxal phosphate-dependent enzyme | LPS |
| *ACXA16_04640* | *ACICU_RS00405* | Polysaccharide biosynthesis/export family protein | Capsule |
| *ACXA16_05955* | *bauE* | Ferric siderophore ABC transporter, ATP-binding protein BauE | Acinetobactin |
| *ACXA16_04520* | *wbaP/rfbP* | Undecaprenyl-phosphate galactosephosphotransferase | LOS |
| *ACXA16_05545* | *msbA* | Lipid transporter ATP-binding/permease | LOS |
| *ACXA16_09935* | *cesC* | ABC transporter ATP-binding protein, CesC | Cereulide |
| *ACXA16_06390* | *adeG* | Cation/multidrug efflux pump | AdeFGH efflux pump |
| *ACXA16_06925* | *FTT_RS04140* | Glycosyltransferase family 2 protein | Capsule |
| *ACXA16_02315* | *dhbE* | 2,3-dihydroxybenzoate adenylase DhbE | Bacillibactin |
| *ACXA16_05865* | *iroN* | Salmochelin receptor IroN | Sal |
| *ACXA16_06945* | *cap8B* | Type 8 capsular polysaccharide synthesis protein Cap8B | Capsule |
| *ACXA16_00010* | *vpsU* | Exopolysaccharide regulatory protein-tyrosine phosphatase VpsU | VPS |
| *ACXA16_03995* | *wcaJ* | Undecaprenyl-phosphate glucose phosphotransferase | Capsule |
| *ACXA16_04865* | *ylxH* | ATP-binding protein | Flagella |
| *ACXA16_04180* | *sigA/rpoV* | RNA polymerase sigma factor SigA | SigA |
| *ACXA16_10650* | *kdsA* | 2-dehydro-3-deoxyphosphooctonate aldolase | LPS |
| *ACXA16_02400* | *cdsN* | Type III secretion system ATPase | TTSS |
| *ACXA16_00505* | *wbpE* | UDP-2-acetamido-2-deoxy-3-oxo-D-glucuronate aminotransferase | LPS |
| *ACXA16_00675* | *wbkA* | Mannosyltransferase | LPS |
| *ACXA16_09040* | *cesC* | ABC transporter ATP-binding protein, CesC | Cereulide |
| *ACXA16_07915* | *katA* | Catalase | KatA |
| *ACXA16_04570* | *wbtD* | Galacturonosyl transferase | LPS |
| *ACXA16_07420* | *fleQ* | Transcriptional regulator FleQ | Flagella |
| *ACXA16_04565* | *ACICU_RS00475* | Sugar transferase | Capsule |
| *ACXA16_00940* | *purM* | Phosphoribosylaminoimidazole synthetase | Purine biosynthesis |
| *ACXA16_05815* | *pdxA* | 4-hydroxythreonine-4-phosphate dehydrogenase | Flagella |
| *ACXA16_07975* | *msbA* | Lipid transporter ATP-binding/permease | LOS |
| *ACXA16_05690* | *acpXL* | Acyl carrier protein | LPS |
| *ACXA16_08255* | *algD* | GDP-mannose 6-dehydrogenase AlgD | Alginate |
| *ACXA16_05195* | *cpsE* | Glycosyl transferase, group 2 family protein | Capsule |
| *ACXA16_08385* | *tufA* | Elongation factor Tu | EF-Tu |
| *ACXA16_02595* | *carB* | Carbamoyl phosphate synthase large subunit | Pyrimidine biosynthesis |
| *ACXA16_06385* | *adeF* | Membrane-fusion protein | AdeFGH efflux pump |
| *ACXA16_00685* | *gtrB* | Bactoprenol glucosyl transferase | LPS |
| *ACXA16_09410* | *vscN* | Type III secretion system ATPase VscN | T3SS1 |
| *ACXA16_03585* | *lpxA* | UDP-N-acetylglucosamine acyltransferase | LOS |
| *ACXA16_00740* | *essC* | Type VII secretion system protein EssC | T7SS |
| *ACXA16_05885* | *adeG* | Cation/multidrug efflux pump | AdeFGH efflux pump |
| *ACXA16_05980* | *brkB* | Serum resistance protein | Brk |
| *ACXA16_06700* | *bplA* | Probable oxidoreductase | LPS |
| *ACXA16_08270* | *msbA* | Lipid transporter ATP-binding/permease | LOS |
| *ACXA16_04595* | *cpsD* | Glycosyl transferase, group 2 family protein | Capsule |
| *ACXA16_10690* | *btrS* | RNA polymerase sigma factor | TTSS |
| *ACXA16_05985* | *dhbA* | 2,3-dihydroxybenzoate-2,3-dehydrogenase, DhbA | Bacillibactin |
| *ACXA16_07025* | *mucP* | Metalloprotease protease | Alginate |
| *ACXA16_08175* | *msbA* | Lipid transporter ATP-binding/permease | LOS |
| *ACXA16_09560* | *iraB* | Di/tripeptide permease | IraAB |
| *ACXA16_00955* | *phzH* | Phenazine-modifying enzyme | Pyocyanin |
| *ACXA16_06425* | *basJ* | Acinetobactin biosynthesis protein BasJ | Acinetobactin |
| *ACXA16_08085* | *vpsD* | Exopolysaccharide biosynthesis glycosyltransferase VpsD | VPS |
| *ACXA16_09035* | *msbA* | Lipid transporter ATP-binding/permease | LOS |
| *ACXA16_07980* | *cpsI* | UDP-galactopyranose mutase | Capsule |
| *ACXA16_01985* | *wbpE* | UDP-2-acetamido-2-deoxy-3-oxo-D-glucuronate aminotransferase | LPS |
| *ACXA16_02290* | *clbF* | Colibactin biosynthesis dehydrogenase ClbF | Colibactin |
| *ACXA16_05175* | *algI* | Alginate o-acetyltransferase AlgI | Alginate |
| *ACXA16_07385* | *bioB* | Biotin synthase | Biotin synthesis |
| *ACXA16_06565* | *letS* | Hybrid sensor histidine kinase/response regulator | LetA/S |
| *ACXA16_05835* | *cylA* | ABC (ATP-binding cassette) transporter CylA | CylA |
| *ACXA16_03150* | *phzG1* | Phenazine biosynthesis protein PhzG | Pyocyanin |
| *ACXA16_00670* | *purCD* | Fusion protein PurC/PurD | Purine biosynthesis |
| *ACXA16_09125* | *phoR* | Two component system response sensor kinase PhoR | PhoR |
| *ACXA16_04210* | *manB/yhxB* | Phosphomannomutase | LOS |
| *ACXA16_09775* | *flgR* | Response regulator | Flagella |
| *ACXA16_05080* | *lpxH* | UDP-2,3-diacylglucosamine pyrophosphohydrolase | LOS |
| *ACXA16_07705* | *katB* | Catalase-peroxidase KatB | KatAB |
| *ACXA16_06695* | *clpC* | Endopeptidase Clp ATP-binding chain C | ClpC |
| *ACXA16_08025* | *FTT_RS04140* | Glycosyltransferase family 2 protein | Capsule |
| *ACXA16_07555* | *motB* | Flagellar motor rotation protein MotB | Flagella |
| *ACXA16_08960* | *pvdH* | Diaminobutyrate-2-oxoglutarate aminotransferase PvdH | Pyoverdine |
| *ACXA16_07825* | *ricA* | Type IV secretion system effector RicA | T4SS secreted effector |
| *ACXA16_04645* | *cap8D* | Type 8 capsular polysaccharide synthesis protein Cap8D | Capsule |
| *ACXA16_04635* | *KP1_RS17330* | Polysaccharide biosynthesis tyrosine autokinase | Capsule |
| *ACXA16_06400* | *algC* | Phosphomannomutase AlgC | Alginate |
| *ACXA16_01565* | *waaV* | Glucosyltransferase | LOS |
| *ACXA16_03140* | *panD* | Aspartate 1-decarboxylase | PanC/PanD |
| *ACXA16_09120* | *phoP* | Two component system response regulator PhoP | PhoP |
| *ACXA16_10665* | *flmH* | Short chain dehydrogenase/reductase family oxidoreductase | Polar flagella |

^a^The genes assigned in the genome of RA-FJ-C1, corresponding to the virulence genes in the Virulence Factors Database (VFDB).

^b^Virulence gene names in VFDB.

^c^Description of the proteins encoded by the virulence genes.

^d^Virulence factors associated with the virulence genes.

**Table S4 Predicted virulence genes of RA-FJ-C4**

| **Gene ID^a^** | **Related genes^b^** | **Products^c^** | **Virulence factors^d^** |
| --- | --- | --- | --- |
| *ACXA17_00330* | *flgS* | Signal transduction histidine kinase | Flagella |
| *ACXA17_04850* | *cap8O* | Type 8 capsular polysaccharide synthesis protein Cap8O | Capsule |
| *ACXA17_02660* | *tlyC* | Hemolysin C | TlyC |
| *ACXA17_01340* | *relA* | Probable GTP pyrophosphokinase RelA | RelA |
| *ACXA17_00540* | *AHML_RS07540* | CobQ/CobB/MinD/ParA family protein | Polar flagella |
| *ACXA17_00510* | *scpA/scpB* | Streptococcal C5a peptidase | C5a peptidase |
| *ACXA17_02830* | *bauE* | Ferric siderophore ABC transporter, ATP-binding protein BauE | Acinetobactin |
| *ACXA17_02565* | *clbF* | Colibactin biosynthesis dehydrogenase ClbF | Colibactin |
| *ACXA17_05365* | *clpC* | Endopeptidase Clp ATP-binding chain C | ClpC |
| *ACXA17_06245* | *bauE* | Ferric siderophore ABC transporter, ATP-binding protein BauE | Acinetobactin |
| *ACXA17_10360* | *bioF* | 8-amino-7-oxononanoate synthase | Biotin synthesis |
| *ACXA17_08395* | *vpsD* | Exopolysaccharide biosynthesis glycosyltransferase VpsD | VPS |
| *ACXA17_06270* | *brkB* | Serum resistance protein | Brk |
| *ACXA17_04195* | *wcaJ* | Undecaprenyl-phosphate glucose phosphotransferase | Capsule |
| *ACXA17_07430* | *manCcore* | Mannose-1-phosphate guanylyltransferase/mannose-6-phosphate isomerase | LPS |
| *ACXA17_08305* | *FTT_RS04140* | Glycosyltransferase family 2 protein | Capsule |
| *ACXA17_06065* | *GBS_RS06570* | Glycosyltransferase | Capsule |
| *ACXA17_06055* | *manCcore* | Mannose-1-phosphate guanylyltransferase/mannose-6-phosphate isomerase | LPS |
| *ACXA17_08560* | *msbA* | Lipid transporter ATP-binding/permease | LOS |
| *ACXA17_09380* | *cesC* | ABC transporter ATP-binding protein, CesC | Cereulide |
| *ACXA17_00635* | *purCD* | Fusion protein PurC/PurD | Purine biosynthesis |
| *ACXA17_07850* | *wbtF* | NAD dependent epimerase | LPS |
| *ACXA17_02670* | *cdsN* | Type III secretion system ATPase | TTSS |
| *ACXA17_07190* | *flhF* | Flagellar biosynthesis regulator FlhF | Flagella |
| *ACXA17_05110* | *ylxH* | ATP-binding protein | Flagella |
| *ACXA17_07230* | *cap8B* | Type 8 capsular polysaccharide synthesis protein Cap8B | Capsule |
| *ACXA17_04750* | *wbtL* | Glucose-1-phosphate thymidylyltransferase | LPS |
| *ACXA17_09565* | *cesC* | ABC transporter ATP-binding protein, CesC | Cereulide |
| *ACXA17_00165* | *Rv2952* | Phthiotriol/phenolphthiotriol dimycocerosates methyltransferase | PDIM |
| *ACXA17_04900* | *pgi* | Glucose-6-phosphate isomerase | Capsule |
| *ACXA17_07735* | *recN* | DNA repair protein RecN | RecN |
| *ACXA17_10745* | *btrS* | RNA polymerase sigma factor | TTSS |
| *ACXA17_02550* | *clbF* | Colibactin biosynthesis dehydrogenase ClbF | Colibactin |
| *ACXA17_08555* | *algD* | GDP-mannose 6-dehydrogenase AlgD | Alginate |
| *ACXA17_09810* | *fleR* | Two-component response regulator | Flagella |
| *ACXA17_02855* | *carA* | Carbamoyl phosphate synthase small subunit | Pyrimidine biosynthesis |
| *ACXA17_06130* | *cylA* | ABC (ATP-binding cassette) transporter CylA | CylA |
| *ACXA17_10040* | *flgJ* | Flagellar rod assembly protein/muramidase FlgJ | Flagella |
| *ACXA17_00010* | *KP1_RS17335* | Protein-tyrosine-phosphatase | Capsule |
| *ACXA17_01260* | *icaA* | N-acetylglucosaminyltransferase | Intercellular adhesion proteins |
| *ACXA17_01350* | *ggt* | Gamma-glutamyltranspeptidase | GGT |
| *ACXA17_06810* | *paa* | Outer membrane adhesin Paa | Paa |
| *ACXA17_06410* | *rfbD* | dTDP-4-dehydrorhamnose reductase | LPS |
| *ACXA17_02020* | *acrB* | Acriflavine resistance protein B | AcrAB |
| *ACXA17_09135* | *kdtB* | Lipopolysaccharide core biosynthesis protein | LPS |
| *ACXA17_08255* | *sodB* | Superoxide dismutase | SodB |
| *ACXA17_06675* | *adeF* | Membrane-fusion protein | AdeFGH efflux pump |
| *ACXA17_04305* | *lpxK* | Tetraacyldisaccharide 4'-kinase | LOS |
| *ACXA17_04780* | *rfbC* | dTDP-4-dehydrorhamnose 3,5-epimerase | LPS |
| *ACXA17_05315* | *lpxH* | UDP-2,3-diacylglucosamine pyrophosphohydrolase | LOS |
| *ACXA17_07915* | *motB* | Flagellar motor rotation protein MotB | Flagella |
| *ACXA17_07000* | *bplA* | Probable oxidoreductase | LPS |
| *ACXA17_08420* | *gluP* | Glucose/galactose transporter | LPS |
| *ACXA17_06690* | *algC* | Phosphomannomutase AlgC | Alginate |
| *ACXA17_02530* | *exsA* | Type III secretion system transcriptional regulator | T3SS |
| *ACXA17_02570* | *dhbE* | 2,3-dihydroxybenzoate adenylase DhbE | Bacillibactin |
| *ACXA17_06785* | *FTT_RS04145* | Glycosyltransferase family 2 protein | Capsule |
| *ACXA17_05445* | *cylG* | 3-ketoacyl-ACP-reductase CylG | CylG |
| *ACXA17_00265* | *orfM* | Deoxyribonucleotide triphosphate pyrophosphatase | LOS |
| *ACXA17_06935* | *iroC* | ABC transporter | Sal |
| *ACXA17_05415* | *algI* | Alginate o-acetyltransferase AlgI | Alginate |
| *ACXA17_00630* | *wbkA* | Mannosyltransferase | LPS |
| *ACXA17_06435* | *cpsA/uppS* | Undecaprenyl diphosphate synthase | Capsule |
| *ACXA17_00440* | *wbpE* | UDP-2-acetamido-2-deoxy-3-oxo-D-glucuronate aminotransferase | LPS |
| *ACXA17_08035* | *katB* | Catalase-peroxidase KatB | KatAB |
| *ACXA17_04760* | *per* | Perosamine synthetase | LPS |
| *ACXA17_09465* | *phoR* | Two component system response sensor kinase membrane associated PhoR | PhoR |
| *ACXA17_08300* | *cpsI* | UDP-galactopyranose mutase | Capsule |
| *ACXA17_09740* | *vscN* | Type III secretion system ATPase VscN | T3SS1 |
| *ACXA17_03515* | *phzG1* | Phenazine biosynthesis protein PhzG, pyridoxamine 5'-phosphate oxidase | Pyocyanin |
| *ACXA17_07750* | *bioB* | Biotin synthase | Biotin synthesis |
| *ACXA17_09815* | *algB* | Two-component response regulator AlgB | Alginate |
| *ACXA17_04445* | *manB /yhxB* | Phosphomannomutase | LOS |
| *ACXA17_03500* | *panD* | Aspartate 1-decarboxylase | PanC/PanD |
| *ACXA17_06715* | *basJ* | Acinetobactin biosynthesis protein BasJ | Acinetobactin |
| *ACXA17_09405* | *panC* | Pantothenate synthetase | PanC/PanD |
| *ACXA17_02480* | *wbpE* | UDP-2-acetamido-2-deoxy-3-oxo-D-glucuronate aminotransferase | LPS |
| *ACXA17_04870* | *wbtF* | NAD dependent epimerase | LPS |
| *ACXA17_07785* | *fleQ* | Transcriptional regulator FleQ | Flagella |
| *ACXA17_04075* | *lpxA* | UDP-N-acetylglucosamine acyltransferase | LOS |
| *ACXA17_02540* | *flmH* | Short chain dehydrogenase/reductase family oxidoreductase | Polar flagella |
| *ACXA17_10560* | *clpP* | ATP-dependent Clp protease proteolytic subunit | ClpP |
| *ACXA17_08315* | *KP1_RS17240* | DUF4422 domain-containing protein | LPS |
| *ACXA17_09700* | *cylA* | ABC (ATP-binding cassette) transporter CylA | CylA |
| *ACXA17_00410* | *kpsF* | D-arabinose 5-phosphate isomerase | Capsule |
| *ACXA17_02840* | *carB* | Carbamoyl phosphate synthase large subunit | Pyrimidine biosynthesis |
| *ACXA17_08300* | *glf* | UDP-galactopyranose mutase | Capsule |
| *ACXA17_02860* | *pyrB* | Aspartate carbamoyltransferase | Pyrimidine biosynthesis |
| *ACXA17_10720* | *flmH* | Short chain dehydrogenase/reductase family oxidoreductase | Polar flagella |
| *ACXA17_02825* | *fepD* | Ferrienterobactin ABC transporter permease | Enterobactin |
| *ACXA17_05780* | *msbA* | Lipid transporter ATP-binding/permease | LOS |
| *ACXA17_09460* | *phoP* | Two component system response transcriptional positive regulator PhoP | PhoP |
| *ACXA17_00870* | *purM* | Phosphoribosylaminoimidazole synthetase | Purine biosynthesis |
| *ACXA17_04860* | *tviB* | UDP-N-acetylglucosamine C-6 dehydrogenase TviB | Capsule |
| *ACXA17_08965* | *prsA2* | Post translocation chaperone PrsA2 | PrsA2 |
| *ACXA17_00205* | *lirB* | Dot/Icm type IV secretion system effector LirB | T4SS secreted effector |
| *ACXA17_00400* | *wbtG* | Glycosyl transferase family protein | LPS |
| *ACXA17_05840* | *mucD* | Serine protease MucD precursor | Alginate |
| *ACXA17_07590* | *feoB* | Fe (2+) transporter permease subunit FeoB | FupA |
| *ACXA17_05835* | *YE_RS15445* | Glycosyltransferase | O-antigen |
| *ACXA17_06110* | *pdxA* | 4-hydroxythreonine-4-phosphate dehydrogenase | Flagella |
| *ACXA17_10135* | *iraB* | Di/tripeptide permease | IraAB |
| *ACXA17_04800* | *ACICU_RS00475* | Sugar transferase | Capsule |
| *ACXA17_07940* | *fepA* | Outer membrane receptor FepA | Ent |
| *ACXA17_00895* | *cesC* | ABC transporter ATP-binding protein, CesC | Cereulide |
| *ACXA17_04430* | *kpsU* | 3-deoxy-manno-octulosonate cytidylyltransferase | K1 capsule |
| *ACXA17_01770* | *hlyB* | Hemolysin B | Hemolysin |
| *ACXA17_04410* | *rpe* | Ribulose-phosphate 3-epimerase | Capsule |
| *ACXA17_04225* | *fadD29* | Long-chain-fatty-acid--AMP ligase FadD29 | PDIM |
| *ACXA17_04580* | *aatC* | ATP-binding protein AatC | Dispersin |
| *ACXA17_06680* | *adeG* | Cation/multidrug efflux pump | AdeFGH efflux pump |
| *ACXA17_06370* | *clpE* | ATP-dependent protease | ClpE |
| *ACXA17_08335* | *lgtA* | N-acetylglucosamine glycosyltransferase | LOS |
| *ACXA17_09290* | *pvdH* | Diaminobutyrate-2-oxoglutarate aminotransferase PvdH | Pyoverdine |
| *ACXA17_07210* | *FTT_RS04140* | Glycosyltransferase family 2 protein | Capsule |
| *ACXA17_08240* | *katA* | Catalase | KatA |
| *ACXA17_04340* | *mig-5* | Putative carbonic anhydrase | Mig-5 |
| *ACXA17_10370* | *bioA* | Adenosylmethionine-8-amino-7-oxononanoate aminotransferase | Biotin synthesis |
| *ACXA17_08675* | *tufA* | Elongation factor Tu | EF-Tu |
| *ACXA17_07685* | *clbG* | Colibactin biosynthesis acyltransferase ClbG | Colibactin |
| *ACXA17_08150* | *ricA* | Type IV secretion system effector RicA | T4SS secreted effector |
| *ACXA17_08900* | *rpoN* | RNA polymerase factor sigma-54 | Type IV pili |
| *ACXA17_09220* | *fepA* | Ferrienterobactin outer membrane transporter | Enterobactin |
| *ACXA17_08490* | *msbA* | Lipid transporter ATP-binding/permease | LOS |
| *ACXA17_07370* | *mucP* | Metalloprotease protease | Alginate |
| *ACXA17_06000* | *acpXL* | Acyl carrier protein | LPS |
| *ACXA17_00885* | *phzH* | Phenazine-modifying enzyme | Pyocyanin |
| *ACXA17_06160* | *iroN* | Salmochelin receptor IroN | Sal |
| *ACXA17_04875* | *KP1_RS17330* | Polysaccharide biosynthesis tyrosine autokinase | Capsule |
| *ACXA17_06960* | *flmH* | Short chain dehydrogenase/reductase family oxidoreductase | Polar flagella |
| *ACXA17_06120* | *fleS/flrB* | Flagellar sensor histidine kinase FleS | Flagella |
| *ACXA17_00565* | *essC* | Type VII secretion system protein EssC | T7SS |
| *ACXA17_06455* | *cpsB/cdsA* | Phosphatidate cytidylyltransferase | Capsule |
| *ACXA17_10705* | *kdsA* | 2-dehydro-3-deoxyphosphooctonate aldolase | LPS |
| *ACXA17_09375* | *msbA* | Lipid transporter ATP-binding/permease | LOS |
| *ACXA17_06365* | *msrA/BpilB* | Trifunctional thioredoxin/methionine sulfoxide reductase A/B protein | MsrAB |
| *ACXA17_08500* | *cdsN* | Type III secretion system ATPase | TTSS |
| *ACXA17_00435* | *ahpC* | Alkyl hydroperoxide reductase subunit AhpC | AhpC |
| *ACXA17_01405* | *ybtS* | Salicylate synthase Irp9 | Yersiniabactin |
| *ACXA17_04855* | *cps4I* | Capsular polysaccharide biosynthesis protein Cps4I | Capsule |
| *ACXA17_04080* | *lpxC* | UDP-3-O-(R-3-hydroxymyristoyl)-N-acetylglucosamine deacetylase | LOS |
| *ACXA17_04845* | *cap8E* | Type 8 capsular polysaccharide synthesis protein Cap8E | Capsule |
| *ACXA17_06970* | *letA* | UvrY/SirA/GacA family response regulator transcription factor | LetA/S |
| *ACXA17_04555* | *pvdJ* | Pyoverdine biosynthesis protein PvdJ | Pyoverdine |
| *ACXA17_04885* | *cap8D* | Type 8 capsular polysaccharide synthesis protein Cap8D | Capsule |
| *ACXA17_06275* | *dhbA* | 2,3-dihydroxybenzoate-2,3-dehydrogenase, DhbA | Bacillibactin |
| *ACXA17_02425* | *htpB* | Hsp60, 60K heat shock protein HtpB | Hsp60 |
| *ACXA17_02475* | *galE* | UDP-glucose 4-epimerase | LOS |
| *ACXA17_03830* | *ompA* | Outer membrane protein A | OmpA |
| *ACXA17_08600* | *mgtB* | Mg2+ transport protein | MgtBC |
| *ACXA17_10690* | *eptC* | Phosphoethanolamine transferase | Flagella |
| *ACXA17_04775* | *rffG* | dTDP-glucose 46-dehydratase | LOS |
| *ACXA17_05425* | *cpsE* | Glycosyl transferase, group 2 family protein | Capsule |
| *ACXA17_10585* | *pilR* | Two-component response regulator PilR | Type IV pili |
| *ACXA17_04790* | *LPG_RS03745* | Aminotransferase class I/II-fold pyridoxal phosphate-dependent enzyme | LPS |
| *ACXA17_06175* | *adeG* | Cation/multidrug efflux pump | AdeFGH efflux pump |
| *ACXA17_06865* | *letS* | Hybrid sensor histidine kinase/response regulator | LetA/S |
| *ACXA17_01430* | *clbD* | Colibactin biosynthesis dehydrogenase ClbD | Colibactin |
| *ACXA17_04785* | *bplG* | Probable sugar transferase | LPS |
| *ACXA17_08325* | *FTT_RS04145* | Glycosyltransferase family 2 protein | Capsule |
| *ACXA17_03345* | *bioF* | 8-amino-7-oxononanoate synthase | Biotin synthesis |
| *ACXA17_04810* | *cps4F* | Capsular polysaccharide biosynthesis protein Cps4F | Capsule |
| *ACXA17_00620* | *gtrB* | Bactoprenol glucosyl transferase | LPS |
| *ACXA17_04415* | *sigA/rpoV* | RNA polymerase sigma factor SigA | SigA |
| *ACXA17_06995* | *clpC* | Endopeptidase Clp ATP-binding chain C | ClpC |
| *ACXA17_00315* | *LPG_RS14985* | OmpA family protein | Flagella |
| *ACXA17_09420* | *wbtH* | Asparagine synthase (glutamine-hydrolyzing) | LPS |

^a^The genes assigned in the genome of RA-FJ-C4, corresponding to the virulence genes in the Virulence Factors Database (VFDB).

^b^Virulence gene names in VFDB.

^c^Description of the proteins encoded by the virulence genes.

^d^Virulence factors associated with the virulence genes.

**Table S5 Predicted virulence genes of RA-BJ-C1**

| **Gene ID^a^** | **Related genes^b^** | **Products^c^** | **Virulence factors^d^** |
| --- | --- | --- | --- |
| *ACXA18_04175* | *tviB* | UDP-N-acetylglucosamine C-6 dehydrogenase TviB | Capsule |
| *ACXA18_06050* | *algC* | Phosphomannomutase AlgC | Alginate |
| *ACXA18_01890* | *clbF* | Colibactin biosynthesis dehydrogenase ClbF | Colibactin |
| *ACXA18_03570* | *fadD29* | Long-chain-fatty-acid--AMP ligase FadD29 | PDIM |
| *ACXA18_06140* | *FTT_RS04145* | Glycosyltransferase family 2 protein | Capsule |
| *ACXA18_07970* | *vpsD* | Exopolysaccharide biosynthesis glycosyltransferase VpsD | VPS |
| *ACXA18_05165* | *mucD* | Serine protease MucD precursor | Alginate |
| *ACXA18_07000* | *manCcore* | Mannose-1-phosphate guanylyltransferase/mannose-6-phosphate isomerase | LPS |
| *ACXA18_01405* | *pce/cbpE* | Choline binding protein E | CBPs |
| *ACXA18_04150* | *FTT_RS04140* | Glycosyltransferase family 2 protein | Capsule |
| *ACXA18_10010* | *bioA* | Adenosylmethionine-8-amino-7-oxononanoate aminotransferase | Biotin synthesis |
| *ACXA18_04105* | *rfbC* | dTDP-4-dehydrorhamnose 3,5-epimerase | LPS |
| *ACXA18_07720* | *ricA* | Type IV secretion system effector RicA | T4SS secreted effector |
| *ACXA18_04110* | *wbtB* | Galactosyl transferase | LPS |
| *ACXA18_00410* | *kpsF* | D-arabinose 5-phosphate isomerase | Capsule |
| *ACXA18_01870* | *exsA* | Type III secretion system transcriptional regulator | T3SS |
| *ACXA18_00615* | *gtrB* | Bactoprenol glucosyl transferase | LPS |
| *ACXA18_04100* | *rffG* | dTDP-glucose 46-dehydratase | LOS |
| *ACXA18_00265* | *orfM* | Deoxyribonucleotide triphosphate pyrophosphatase | LOS |
| *ACXA18_03790* | *manB/yhxB* | Phosphomannomutase | LOS |
| *ACXA18_06940* | *mucP* | Metalloprotease protease | Alginate |
| *ACXA18_04170* | *cps4I* | Capsular polysaccharide biosynthesis protein Cps4I | Capsule |
| *ACXA18_10000* | *bioF* | 8-amino-7-oxononanoate synthase | Biotin synthesis |
| *ACXA18_03775* | *kpsU* | 3-deoxy-manno-octulosonate cytidylyltransferase | K1 capsule |
| *ACXA18_04195* | *ACICU_RS00405* | Polysaccharide biosynthesis/export family protein | Capsule |
| *ACXA18_06550* | *flhF* | Flagellar biosynthesis regulator FlhF | Flagella |
| *ACXA18_05690* | *msrA/BpilB* | Trifunctional thioredoxin/methionine sulfoxide reductase A/B protein | MsrAB |
| *ACXA18_02600* | *phzG1* | Phenazine biosynthesis protein PhzG, pyridoxamine 5'-phosphate oxidase | Pyocyanin |
| *ACXA18_04135* | *YE_RS15445* | Glycosyltransferase | O-antigen |
| *ACXA18_01130* | *ybtS* | Salicylate synthase Irp9 | Yersiniabactin |
| *ACXA18_05105* | *msbA* | Lipid transporter ATP-binding/permease | LOS |
| *ACXA18_00165* | *Rv2952* | Phthiotriol/phenolphthiotriol dimycocerosates methyltransferase | PDIM |
| *ACXA18_03420* | *lpxA* | UDP-N-acetylglucosamine acyltransferase | LOS |
| *ACXA18_02000* | *tlyC* | Hemolysin C | TlyC |
| *ACXA18_00985* | *icaA* | N-acetylglucosaminyltransferase | Intercellular adhesion proteins |
| *ACXA18_06590* | *cap8B* | Type 8 capsular polysaccharide synthesis protein Cap8B | Capsule |
| *ACXA18_09275* | *cylA* | ABC (ATP-binding cassette) transporter CylA | CylA |
| *ACXA18_06570* | *FTT_RS04140* | Glycosyltransferase family 2 protein | Capsule |
| *ACXA18_04425* | *ylxH* | ATP-binding protein | Flagella |
| *ACXA18_04755* | *cpsE* | Glycosyl transferase, group 2 family protein | Capsule |
| *ACXA18_08875* | *pvdH* | Diaminobutyrate-2-oxoglutarate aminotransferase PvdH | Pyoverdine |
| *ACXA18_09040* | *phoR* | Two component system response sensor kinase membrane associated PhoR | PhoR |
| *ACXA18_10660* | *eptC* | Phosphoethanolamine transferase | Flagella |
| *ACXA18_09775* | *iraB* | Di/tripeptide permease | IraAB |
| *ACXA18_01060* | *relA* | Probable GTP pyrophosphokinase RelA | RelA |
| *ACXA18_01820* | *wbpE* | UDP-2-acetamido-2-deoxy-3-oxo-D-glucuronate aminotransferase | LPS |
| *ACXA18_00560* | *essC* | type VII secretion system protein EssC | T7SS |
| *ACXA18_04185* | *wbtF* | NAD dependent epimerase | LPS |
| *ACXA18_10530* | *clpP* | ATP-dependent Clp protease proteolytic subunit | ClpP |
| *ACXA18_05390* | *GBS_RS06570* | Glycosyltransferase | Capsule |
| *ACXA18_08230* | *tufA* | Elongation factor Tu | EF-Tu |
| *ACXA18_07320* | *bioB* | Biotin synthase | Biotin synthesis |
| *ACXA18_04690* | *clpC* | Endopeptidase Clp ATP-binding chain C | ClpC |
| *ACXA18_09035* | *phoP* | Two component system response transcriptional positive regulator PhoP | PhoP |
| *ACXA18_05160* | *YE_RS15445* | Glycosyltransferase | O-antigen |
| *ACXA18_02125* | *fepD* | Ferrienterobactin ABC transporter permease | Enterobactin |
| *ACXA18_08980* | *panC* | Pantothenate synthetase | PanC/PanD |
| *ACXA18_05600* | *dhbA* | 2,3-dihydroxybenzoate-2,3-dehydrogenase, DhbA | Bacillibactin |
| *ACXA18_07620* | *katB* | Catalase-peroxidase KatB | KatAB |
| *ACXA18_03650* | *lpxK* | Tetraacyldisaccharide 4'-kinase | LOS |
| *ACXA18_08450* | *rpoN* | RNA polymerase factor sigma-54 | Type IV pili |
| *ACXA18_05805* | *cpsB/cdsA* | Phosphatidate cytidylyltransferase | Capsule |
| *ACXA18_05380* | *manCcore* | Mannose-1-phosphate guanylyltransferase/mannose-6-phosphate isomerase | LPS |
| *ACXA18_10675* | *kdsA* | 2-dehydro-3-deoxyphosphooctonate aldolase | LPS |
| *ACXA18_00900* | *cesC* | ABC transporter ATP-binding protein, CesC | Cereulide |
| *ACXA18_04130* | *wbtD* | Galacturonosyl transferase | LPS |
| *ACXA18_00630* | *purCD* | Fusion protein PurC/PurD | Purine biosynthesis |
| *ACXA18_08055* | *cdsN* | Type III secretion system ATPase | TTSS |
| *ACXA18_03425* | *lpxC* | UDP-3-O-(R-3-hydroxymyristoyl)-N-acetylglucosamine deacetylase | LOS |
| *ACXA18_05760* | *rfbD* | dTDP-4-dehydrorhamnose reductase | LPS |
| *ACXA18_05445* | *fleS/flrB* | Flagellar sensor histidine kinase FleS | Flagella |
| *ACXA18_07900* | *FTT_RS04145* | Glycosyltransferase family 2 protein | Capsule |
| *ACXA18_05595* | *brkB* | Serum resistance protein | Brk |
| *ACXA18_06290* | *iroC* | ABC transporter | Sal |
| *ACXA18_04640* | *lpxH* | UDP-2,3-diacylglucosamine pyrophosphohydrolase | LOS |
| *ACXA18_07885* | *KP1_RS17240* | DUF4422 domain-containing protein | LPS |
| *ACXA18_05500* | *adeG* | Cation/multidrug efflux pump | AdeFGH efflux pump |
| *ACXA18_02130* | *bauE* | Ferric siderophore ABC transporter, ATP-binding protein BauE | Acinetobactin |
| *ACXA18_09330* | *vscN* | Type III secretion system ATPase VscN | T3SS1 |
| *ACXA18_02160* | *pyrB* | Aspartate carbamoyltransferase | Pyrimidine biosynthesis |
| *ACXA18_00435* | *ahpC* | Alkyl hydroperoxide reductase subunit AhpC | AhpC |
| *ACXA18_00315* | *LPG_RS14985* | OmpA family protein | Flagella |
| *ACXA18_07255* | *clbG* | Colibactin biosynthesis acyltransferase ClbG | Colibactin |
| *ACXA18_07820* | *sodB* | Superoxide dismutase | SodB |
| *ACXA18_07525* | *fepA* | Outer membrane receptor FepA | Ent |
| *ACXA18_07865* | *glf* | UDP-galactopyranose mutase | Capsule |
| *ACXA18_10690* | *flmH* | Short chain dehydrogenase/reductase family oxidoreductase | Polar flagella |
| *ACXA18_05325* | *acpXL* | Acyl carrier protein | LPS |
| *ACXA18_03760* | *sigA/rpoV* | RNA polymerase sigma factor SigA | SigA |
| *ACXA18_06315* | *flmH* | Short chain dehydrogenase/reductase family oxidoreductase | Polar flagella |
| *ACXA18_08815* | *fepA* | Ferrienterobactin outer membrane transporter | Enterobactin |
| *ACXA18_00625* | *wbkA* | Mannosyltransferase | LPS |
| *ACXA18_02585* | *panD* | Aspartate 1-decarboxylase | PanC/PanD |
| *ACXA18_08115* | *msbA* | Lipid transporter ATP-binding/permease | LOS |
| *ACXA18_03540* | *wcaJ* | Undecaprenyl-phosphate glucose phosphotransferase | Capsule |
| *ACXA18_04215* | *pgi* | Glucose-6-phosphate isomerase | Capsule |
| *ACXA18_04775* | *cylG* | 3-ketoacyl-ACP-reductase CylG | CylG |
| *ACXA18_00890* | *phzH* | Phenazine-modifying enzyme | Pyocyanin |
| *ACXA18_08155* | *mgtB* | Mg2+ transport protein | MgtBC |
| *ACXA18_00400* | *wbtG* | Glycosyl transferase family protein | LPS |
| *ACXA18_01765* | *htpB* | Hsp60, 60K heat shock protein HtpB | Hsp60 |
| *ACXA18_05485* | *iroN* | Salmochelin receptor IroN | Sal |
| *ACXA18_01815* | *galE* | UDP-glucose 4-epimerase | LOS |
| *ACXA18_06325* | *letA* | UvrY/SirA/GacA family response regulator transcription factor | LetA/S |
| *ACXA18_05570* | *bauE* | Ferric siderophore ABC transporter, ATP-binding protein BauE | Acinetobactin |
| *ACXA18_08995* | *wbtH* | Asparagine synthase (glutamine-hydrolyzing) | LPS |
| *ACXA18_10130* | *cap8J* | Type 8 capsular polysaccharide synthesis protein Cap8J | Capsule |
| *ACXA18_07160* | *feoB* | Fe(2+) transporter permease subunit FeoB | FupA |
| *ACXA18_07420* | *wbtF* | NAD dependent epimerase | LPS |
| *ACXA18_04160* | *FTT_RS04140* | Glycosyltransferase family 2 protein | Capsule |
| *ACXA18_06040* | *FTT_RS04140* | Cation/multidrug efflux pump | AdeFGH efflux pump |
| *ACXA18_06075* | *basJ* | Acinetobactin biosynthesis protein BasJ | Acinetobactin |
| *ACXA18_08110* | *algD* | GDP-mannose 6-dehydrogenase AlgD | Alginate |
| *ACXA18_01910* | *dhbE* | 2,3-dihydroxybenzoate adenylase DhbE | Bacillibactin |
| *ACXA18_05435* | *pdxA* | 4-hydroxythreonine-4-phosphate dehydrogenase | Flagella |
| *ACXA18_08310* | *bauE* | Ferric siderophore ABC transporter, ATP-binding protein BauE | Acinetobactin |
| *ACXA18_08685* | *kdtB* | Lipopolysaccharide core biosynthesis protein | LPS |
| *ACXA18_05455* | *cylA* | ABC (ATP-binding cassette) transporter CylA | CylA |
| *ACXA18_00875* | *purM* | Phosphoribosylaminoimidazole synthetase | Purine biosynthesis |
| *ACXA18_01070* | *ggt* | Gamma-glutamyltranspeptidase | GGT |
| *ACXA18_02430* | *bioF* | 8-amino-7-oxononanoate synthase | Biotin synthesis |
| *ACXA18_07870* | *FTT_RS04140* | Glycosyltransferase family 2 protein | Capsule |
| *ACXA18_01880* | *flmH* | Short chain dehydrogenase/reductase family oxidoreductase | Polar flagella |
| *ACXA18_01370* | *acrB* | Acriflavine resistance protein B | AcrAB |
| *ACXA18_03685* | *mig-5* | Putative carbonic anhydrase | Mig-5 |
| *ACXA18_06035* | *adeF* | Membrane-fusion protein | AdeFGH efflux pump |
| *ACXA18_07355* | *fleQ* | Transcriptional regulator FleQ | Flagella |
| *ACXA18_02155* | *carA* | Carbamoyl phosphate synthase small subunit | Pyrimidine biosynthesis |
| *ACXA18_01905* | *clbF* | Colibactin biosynthesis dehydrogenase ClbF | Colibactin |
| *ACXA18_03755* | *rpe* | Ribulose-phosphate 3-epimerase | Capsule |
| *ACXA18_00535* | *AHML_RS07540* | CobQ/CobB/MinD/ParA family protein | Polar flagella |
| *ACXA18_00440* | *wbpE* | UDP-2-acetamido-2-deoxy-3-oxo-D-glucuronate aminotransferase | LPS |
| *ACXA18_00010* | *KP1_RS17335* | Protein-tyrosine-phosphatase | Capsule |
| *ACXA18_06355* | *clpC* | Endopeptidase Clp ATP-binding chain C | ClpC |
| *ACXA18_01155* | *clbD* | Colibactin biosynthesis dehydrogenase ClbD | Colibactin |
| *ACXA18_06220* | *letS* | Hybrid sensor histidine kinase/response regulator | LetA/S |
| *ACXA18_08515* | *prsA2* | Post translocation chaperone PrsA2 | PrsA2 |
| *ACXA18_03900* | *pvdJ* | Pyoverdine biosynthesis protein PvdJ | Pyoverdine |
| *ACXA18_07910* | *lgtA* | N-acetylglucosamine glycosyltransferase | LOS |
| *ACXA18_02840* | *ompA* | Outer membrane protein A | OmpA |
| *ACXA18_08045* | *msbA* | Lipid transporter ATP-binding/permease | LOS |
| *ACXA18_06165* | *paa* | Outer membrane adhesin Paa | Paa |
| *ACXA18_08955* | *cesC* | ABC transporter ATP-binding protein, CesC | Cereulide |
| *ACXA18_07305* | *recN* | DNA repair protein RecN | RecN |
| *ACXA18_08950* | *msbA* | Lipid transporter ATP-binding/permease | LOS |
| *ACXA18_09140* | *cesC* | ABC transporter ATP-binding protein, CesC | Cereulide |
| *ACXA18_09395* | *flgJ* | Flagellar rod assembly protein/muramidase FlgJ | Flagella |
| *ACXA18_04125* | *ACICU_RS00475* | Sugar transferase | Capsule |
| *ACXA18_02140* | *carB* | Carbamoyl phosphate synthase large subunit | Pyrimidine biosynthesis |
| *ACXA18_07995* | *gluP* | Glucose/galactose transporter | LPS |
| *ACXA18_00330* | *flgS* | Signal transduction histidine kinase | Flagella |
| *ACXA18_07500* | *motB* | Flagellar motor rotation protein MotB | Flagella |
| *ACXA18_03925* | *aatC* | ATP-binding protein AatC | Dispersin |
| *ACXA18_00505* | *scpA/scpB* | Streptococcal C5a peptidase | C5a peptidase |
| *ACXA18_07805* | *katA* | Catalase | KatA |
| *ACXA18_06360* | *bplA* | Probable oxidoreductase | LPS |
| *ACXA18_04200* | *cap8D* | Type 8 capsular polysaccharide synthesis protein Cap8D | Capsule |
| *ACXA18_10715* | *btrS* | RNA polymerase sigma factor | TTSS |
| *ACXA18_10555* | *pilR* | Two-component response regulator PilR | Type IV pili |
| *ACXA18_04745* | *algI* | Alginate o-acetyltransferase AlgI | Alginate |
| *ACXA18_02010* | *cdsN* | Type III secretion system ATPase | TTSS |
| *ACXA18_05785* | *cpsA/uppS* | Undecaprenyl diphosphate synthase | Capsule |
| *ACXA18_05695* | *clpE* | ATP-dependent protease | ClpE |
| *ACXA18_04190* | *KP1_RS17330* | Polysaccharide biosynthesis tyrosine autokinase | Capsule |
| *ACXA18_04115* | *LPG_RS03745* | Aminotransferase class I/II-fold pyridoxal phosphate-dependent enzyme | LPS |
| *ACXA18_04095* | *wbtL* | Glucose-1-phosphate thymidylyltransferase | LPS |

^a^The genes assigned in the genome of RA-BJ-C1, corresponding to the virulence genes in the Virulence Factors Database (VFDB).

^b^Virulence gene names in VFDB.

^c^Description of the proteins encoded by the virulence genes.

^d^Virulence factors associated with the virulence genes.

**Table S6 Predicted resistance genes of RA-FJ-C1**

| **Gene ID^a^** | **ARO Name^b^** | **Resistance Mechanism** | **Identity (%)** | **Coverage (%)** | **E-value** |
| --- | --- | --- | --- | --- | --- |
| *ACXA16_01110* | *Clostridioides difficile gyrA* conferring resistance to fluoroquinolones | Antibiotic target alteration | 29.4 | 55.5 | ≤ 1e-5 |
| *ACXA16_02320* | *tet*(36) | Antibiotic target protection | 27.9 | 85.6 | ≤ 1e-5 |
| *ACXA16_07290* | *Streptomyces cinnamoneus* EF-Tu mutants conferring resistance to elfamycin | Antibiotic target alteration | 28 | 21.6 | ≤ 1e-5 |
| *ACXA16_06165* | *Streptococcus mitis* *CdsA* with mutation conferring daptomycin resistance | Antibiotic target alteration | 36.7 | 53.1 | ≤ 1e-5 |
| *ACXA16_08745* | *vanH* gene in *vanA* cluster | Antibiotic target alteration | 34.5 | 78.7 | ≤ 1e-5 |
| *ACXA16_09980* | *ErmF* | Antibiotic target alteration | 97.4 | 99.6 | ≤ 1e-5 |
| *ACXA16_01635* | *Clostridioides difficile* *gyrB* conferring resistance to fluoroquinolones | Antibiotic target alteration | 57 | 98 | ≤ 1e-5 |
| *ACXA16_03555* | *Mycobacterium tuberculosis thyA* with mutation conferring resistance to para-aminosalicylic acid | Antibiotic target alteration | 65.2 | 99.6 | ≤ 1e-5 |
| *ACXA16_03095* | *macB* | Antibiotic efflux | 36.9 | 99.5 | ≤ 1e-5 |
| *ACXA16_09965* | *OXA-209* | Antibiotic inactivation | 86.5 | 99.6 | ≤ 1e-5 |
| *ACXA16_01500* | *MexF* | Antibiotic efflux | 39.8 | 97.8 | ≤ 1e-5 |
| *ACXA16_06495* | *Staphylococcus aureus fusA* with mutation conferring resistance to fusidic acid | Antibiotic target alteration | 24.9 | 63.5 | ≤ 1e-5 |
| *ACXA16_10010* | *tet*(X) | Antibiotic inactivation | 99 | 99.7 | ≤ 1e-5 |
| *ACXA16_10550* | *TxR* | Antibiotic efflux | 45.3 | 59.1 | ≤ 1e-5 |
| *ACXA16_00205* | *Helicobacter pylori pbp1* mutants conferring resistance to amoxicillin | Antibiotic target alteration | 41.1 | 69.6 | ≤ 1e-5 |
| *ACXA16_09935* | *lmrC* | Antibiotic target protection | 33.1 | 99.6 | ≤ 1e-5 |
| *ACXA16_09365* | *bcrA* | Antibiotic efflux | 34.7 | 88.4 | ≤ 1e-5 |
| *ACXA16_00050* | *bacA* | Antibiotic target alteration | 41.6 | 97.8 | ≤ 1e-5 |
| *ACXA16_01625* | *Capnocytophaga gingivalis gyrA* conferring resistance to fluoroquinolones | Antibiotic target alteration | 67.7 | 98.2 | ≤ 1e-5 |
| *ACXA16_01810* | *Mycobacterium tuberculosis pncA* mutations conferring resistance to pyrazinamide | Antibiotic target alteration | 38.2 | 82.1 | ≤ 1e-5 |
| *ACXA16_05425* | *Thermus thermophilus uL*3 mutations conferring resistance to pleuromutilin antibiotics | Antibiotic target alteration | 52.5 | 98.1 | ≤ 1e-5 |
| *ACXA16_04345* | *optrA* | Antibiotic target protection | 33.4 | 98.3 | ≤ 1e-5 |
| *ACXA16_10635* | *MCR-8.4* | Antibiotic target alteration | 34.4 | 85 | ≤ 1e-5 |
| *ACXA16_08450* | *Escherichia coli rpoB* mutants conferring resistance to rifampicin | Antibiotic target alteration or replacement | 45.7 | 98 | ≤ 1e-5 |
| *ACXA16_09120* | *vanR* gene in *vanF* cluster | Antibiotic target alteration | 39 | 98.2 | ≤ 1e-5 |
| *ACXA16_04495* | *DfrA38* | Antibiotic target replacement | 42.9 | 95.7 | ≤ 1e-5 |
| *ACXA16_04220* | *PmrF* | Antibiotic target alteration | 33.1 | 98.7 | ≤ 1e-5 |
| *ACXA16_07195* | *Helicobacter pylori frxA* mutation conferring resistance to metronidazole | Antibiotic target alteration | 26.2 | 84.8 | ≤ 1e-5 |
| *ACXA16_06475* | *Staphylococcus aureus mupB* conferring resistance to mupirocin | Antibiotic target alteration | 37.4 | 95.8 | ≤ 1e-5 |
| *ACXA16_04000* | *RanB* | Antibiotic efflux | 100 | 99.6 | ≤ 1e-5 |
| *ACXA16_04530* | *Escherichia coli fabG* mutations conferring resistance to triclosan | Antibiotic target alteration | 28.2 | 96.8 | ≤ 1e-5 |
| *ACXA16_05350* | *Staphylococcus aureus fusE* with mutation conferring resistance to fusidic acid | Antibiotic target alteration | 48.9 | 98.9 | ≤ 1e-5 |
| *ACXA16_06535* | *lmrD* | Antibiotic efflux | 29.1 | 92.7 | ≤ 1e-5 |
| *ACXA16_07270* | *Mycobacterium tuberculosis folC* with mutation conferring resistance to para-aminosalicylic acid | Antibiotic target alteration | 27.9 | 80.2 | ≤ 1e-5 |
| *ACXA16_05685* | *Mycobacterium tuberculosis kasA* mutant conferring resistance to isoniazid | Antibiotic target alteration | 38.4 | 98.3 | ≤ 1e-5 |
| *ACXA16_08385* | *Enterococcus faecium EF-Tu* mutants conferring resistance to GE2270A | Antibiotic target alteration | 70.5 | 99.2 | ≤ 1e-5 |
| *ACXA16_06765* | *Neisseria gonorrhoeae PBP1* conferring resistance to beta-lactam Antibiotics | Antibiotic target alteration | 29.9 | 86.2 | ≤ 1e-5 |
| *ACXA16_07760* | *Staphylococcus aureus fusA* with mutation conferring resistance to fusidic acid | Antibiotic target alteration | 53.3 | 99.3 | ≤ 1e-5 |
| *ACXA16_06395* | *OprA* | Antibiotic efflux | 26.4 | 88.3 | ≤ 1e-5 |
| *ACXA16_03590* | *LpxC* | Antibiotic target alteration | 29.1 | 63.4 | ≤ 1e-5 |
| *ACXA16_01100* | *Clostridioides difficile gyrB* conferring resistance to fluoroquinolones | Antibiotic target alteration | 33.7 | 96.8 | ≤ 1e-5 |
| *ACXA16_09995* | *tet(X)* | Antibiotic inactivation | 99 | 99.7 | ≤ 1e-5 |
| *ACXA16_07705* | *Mycobacterium tuberculosis katG* mutations conferring resistance to isoniazid | Antibiotic target alteration | 54.9 | 95.4 | ≤ 1e-5 |
| *ACXA16_01500* | *adeB* | Antibiotic efflux | 23.7 | 98.4 | ≤ 1e-5 |
| *ACXA16_03960* | *arnA* | Antibiotic target alteration | 31.2 | 83 | ≤ 1e-5 |
| *ACXA16_05835* | *mlaF* | Antibiotic efflux | 25.1 | 83.9 | ≤ 1e-5 |
| *ACXA16_07420* | *TxR* | Antibiotic efflux | 39.6 | 60.4 | ≤ 1e-5 |
| *ACXA16_06570* | *vanR* gene in *vanL* cluster | Antibiotic target alteration | 29.2 | 90.8 | ≤ 1e-5 |
| *ACXA16_03120* | *Escherichia coli folP* with mutation conferring resistance to sulfonamides | Antibiotic target alteration | 37.3 | 96.7 | ≤ 1e-5 |
| *ACXA16_00975* | *bcrA* | Antibiotic efflux | 32.9 | 71.8 | ≤ 1e-5 |
| *ACXA16_05160* | *Agrobacterium* fabrum chloramphenicol acetyltransferase | Antibiotic inactivation | 43.4 | 28.4 | ≤ 1e-5 |
| *ACXA16_07545* | *mecC* | Antibiotic target replacement | 27.9 | 74.4 | ≤ 1e-5 |
| *ACXA16_09975* | *floR* | Antibiotic efflux | 99.3 | 98 | ≤ 1e-5 |
| *ACXA16_01415* | *Mycobacterium tuberculosis rpsA* mutations conferring resistance to pyrazinamide | Antibiotic target alteration | 38.6 | 55.1 | ≤ 1e-5 |
| *ACXA16_05215* | *Escherichia coli fabI* mutations conferring resistance to isoniazid and triclosan | Antibiotic target alteration | 28.7 | 84.2 | ≤ 1e-5 |
| *ACXA16_03365* | *OmpA* | Reduced permeability to antibiotic | 52.9 | 10.3 | ≤ 1e-5 |
| *ACXA16_07765* | *rpsJ* | Antibiotic target protection | 65 | 98 | ≤ 1e-5 |
| *ACXA16_05955* | *optrA* | Antibiotic target protection | 26.4 | 96.5 | ≤ 1e-5 |
| *ACXA16_04240* | *Staphylococcus aureus mupA* conferring resistance to mupirocin | Antibiotic target alteration | 25.4 | 76 | ≤ 1e-5 |
| *ACXA16_09265* | *CRP* | Antibiotic efflux | 25 | 85.8 | ≤ 1e-5 |
| *ACXA16_06645* | *vanT* gene in *vanG* cluster | Antibiotic target alteration | 33.6 | 43.4 | ≤ 1e-5 |
| *ACXA16_08255* | *ugd* | Antibiotic target alteration | 29 | 81.7 | ≤ 1e-5 |
| *ACXA16_10000* | *DfrA36* | Antibiotic target replacement | 67.3 | 69.8 | ≤ 1e-5 |
| *ACXA16_10005* | *catB8* | Antibiotic inactivation | 71.5 | 98.6 | ≤ 1e-5 |
| *ACXA16_02810* | *ykkD* | Antibiotic efflux | 40.4 | 98.1 | ≤ 1e-5 |
| *ACXA16_07975* | *msbA* | Antibiotic efflux | 30.4 | 68.3 | ≤ 1e-5 |
| *ACXA16_08270* | *msbA* | Antibiotic efflux | 42.1 | 87.5 | ≤ 1e-5 |
| *ACXA16_09040* | *bcrA* | Antibiotic efflux | 36.7 | 92.6 | ≤ 1e-5 |
| *ACXA16_05545* | *tetA(60)* | Antibiotic efflux | 29.8 | 99.1 | ≤ 1e-5 |
| *ACXA16_09035* | *msbA* | Antibiotic efflux | 34.6 | 87.3 | ≤ 1e-5 |
| *ACXA16_05885* | *mdtC* | Antibiotic efflux | 29.9 | 97.8 | ≤ 1e-5 |
| *ACXA16_05825* | *vanS* gene in *vanL* cluster | Antibiotic target alteration | 36.6 | 21.7 | ≤ 1e-5 |
| *ACXA16_00390* | *vanS* gene in *vanL* cluster | Antibiotic target alteration | 28.1 | 47.2 | ≤ 1e-5 |
| *ACXA16_06015* | *Clostridioides difficile murG* with mutation conferring resistance to vancomycin | Antibiotic target alteration | 28.1 | 82.4 | ≤ 1e-5 |
| *ACXA16_08470* | TaeA | Antibiotic efflux | 42.6 | 99.5 | ≤ 1e-5 |
| *ACXA16_06580* | *Borreliella burgdorferi murA* with mutation conferring resistance to fosfomycin | Antibiotic target alteration | 47.9 | 99.1 | ≤ 1e-5 |
| *ACXA16_06385* | *oqxA* | Antibiotic efflux | 31.6 | 79.7 | ≤ 1e-5 |
| *ACXA16_10665* | *Escherichia coli fabG* mutations conferring resistance to triclosan | Antibiotic target alteration | 29.9 | 72.3 | ≤ 1e-5 |
| *ACXA16_02580* | *salE* | Antibiotic target protection | 28.8 | 61.3 | ≤ 1e-5 |
| *ACXA16_00065* | *Erm(52)* | Antibiotic target alteration | 26 | 67.9 | ≤ 1e-5 |
| *ACXA16_00685* | *PmrF* | Antibiotic target alteration | 31.8 | 98.4 | ≤ 1e-5 |
| *ACXA16_02725* | *cfr(B)* | Antibiotic target alteration | 31.1 | 91 | ≤ 1e-5 |
| *ACXA16_01970* | *Mycobacterium tuberculosis gidB* mutation conferring resistance to streptomycin | Antibiotic target alteration | 26.5 | 52.6 | ≤ 1e-5 |
| *ACXA16_04005* | *RanA* | Antibiotic efflux | 98.4 | 99.6 | ≤ 1e-5 |
| *ACXA16_08175* | *msbA* | Antibiotic efflux | 35.7 | 84.8 | ≤ 1e-5 |
| *ACXA16_09220* | *bcrA* | Antibiotic efflux | 32.9 | 86.5 | ≤ 1e-5 |
| *ACXA16_09990* | *aadS* | Antibiotic inactivation | 100 | 99.7 | ≤ 1e-5 |
| *ACXA16_10545* | *abeM* | Antibiotic efflux | 32.6 | 96.6 | ≤ 1e-5 |
| *ACXA16_08775* | *vanE* | Antibiotic target alteration | 32.5 | 96.9 | ≤ 1e-5 |
| *ACXA16_02425* | *tet(41)* | Antibiotic efflux | 40 | 89.9 | ≤ 1e-5 |
| *ACXA16_06665* | *Escherichia coli fabG* mutations conferring resistance to triclosan | Antibiotic target alteration | 51.9 | 97.2 | ≤ 1e-5 |
| *ACXA16_01275* | *Streptomyces rimosus otr(A)* | Antibiotic target protection | 28.5 | 48.3 | ≤ 1e-5 |
| *ACXA16_00650* | *vanH* gene in *vanF* cluster | Antibiotic target alteration | 31.3 | 65.9 | ≤ 1e-5 |
| *ACXA16_09125* | *vanS* gene in *vanG* cluster | Antibiotic target alteration | 28.8 | 69.4 | ≤ 1e-5 |
| *ACXA16_07750* | *Mycobacterium tuberculosis rpsL* mutations conferring resistance to Streptomycin | Antibiotic target alteration | 76.4 | 96.1 | ≤ 1e-5 |
| *ACXA16_09950* | *tet(X4)* | Antibiotic inactivation | 95.8 | 98.4 | ≤ 1e-5 |
| *ACXA16_08455* | *Helicobacter pylori rpoB* mutation conferring resistance to rifampicin | Antibiotic target alteration or replacement | 45 | 98.4 | ≤ 1e-5 |

^a^Resistance genes in the RA-FJ-C1 genome predicted by CARD.

^b^The standardized entry name assigned to an antimicrobial resistance determinant in the Antibiotic Resistance Ontology (ARO), which typically represents a specific resistance gene, mutation, mechanism, or drug class.

**Table S7 Predicted resistance genes of RA-FJ-C4**

| **Gene ID^a^** | **ARO Name^b^** | **Resistance Mechanism** | **Identity (%)** | **Coverage (%)** | **E-value** |
| --- | --- | --- | --- | --- | --- |
| *ACXA17_08560* | *msbA* | Antibiotic efflux | 36.4 | 45.2 | ≤ 1e-5 |
| *ACXA17_08745* | *Escherichia coli rpoB* mutants conferring resistance to rifampicin | Antibiotic target alteration or replacement | 45.6 | 98 | ≤ 1e-5 |
| *ACXA17_08065* | *Mycobacterium tuberculosis rpsL* mutations conferring resistance to Streptomycin | Antibiotic target alteration | 76.4 | 96.1 | ≤ 1e-5 |
| *ACXA17_01335* | *Clostridioides difficile gyrA* conferring resistance to fluoroquinolones | Antibiotic target alteration | 30.3 | 52.4 | ≤ 1e-5 |
| *ACXA17_02575* | *tet(36)* | Antibiotic target protection | 27.9 | 85.6 | ≤ 1e-5 |
| *ACXA17_03830* | *OmpA* | Reduced permeability to antibiotic | 52.9 | 10.3 | ≤ 1e-5 |
| *ACXA17_02020* | *adeB* | Antibiotic efflux | 23.9 | 98.4 | ≤ 1e-5 |
| *ACXA17_10440* | *tet(X4)* | Antibiotic inactivation | 94.7 | 99.7 | ≤ 1e-5 |
| *ACXA17_00895* | *bcrA* | Antibiotic efflux | 32.9 | 69 | ≤ 1e-5 |
| *ACXA17_08765* | *TaeA* | Antibiotic efflux | 42.6 | 99.5 | ≤ 1e-5 |
| *ACXA17_02830* | *salE* | Antibiotic target protection | 29.3 | 61.3 | ≤ 1e-5 |
| *ACXA17_08175* | *vanZ* gene in *vanF* cluster | Antibiotic target alteration | 32.4 | 48.2 | ≤ 1e-5 |
| *ACXA17_07635* | *Mycobacterium tuberculosis folC* with mutation conferring resistance to para-aminosalicylic acid | Antibiotic target alteration | 27.7 | 80.2 | ≤ 1e-5 |
| *ACXA17_09700* | *bcrA* | Antibiotic efflux | 34.7 | 88.4 | ≤ 1e-5 |
| *ACXA17_03480* | *Escherichia coli folP* with mutation conferring resistance to sulfonamides | Antibiotic target alteration | 38.7 | 92.3 | ≤ 1e-5 |
| *ACXA17_07905* | *Helicobacter pylori pbp2* mutants conferring resistance to amoxicillin | Antibiotic target alteration | 25.5 | 81.5 | ≤ 1e-5 |
| *ACXA17_02695* | *tet(41)* | Antibiotic efflux | 40.8 | 89.9 | ≤ 1e-5 |
| *ACXA17_07560* | *Helicobacter pylori frxA* mutation conferring resistance to metronidazole | Antibiotic target alteration | 26.2 | 84.8 | ≤ 1e-5 |
| *ACXA17_06120* | *vanS* gene in *vanL* cluster | Antibiotic target alteration | 37.8 | 23.1 | ≤ 1e-5 |
| *ACXA17_00050* | *bacA* | Antibiotic target alteration | 40.9 | 97.8 | ≤ 1e-5 |
| *ACXA17_09130* | *vanE* | Antibiotic target alteration | 31.9 | 96.9 | ≤ 1e-5 |
| *ACXA17_09810* | *evgS* | Antibiotic efflux | 27.8 | 58.8 | ≤ 1e-5 |
| *ACXA17_04475* | *Staphylococcus aureus mupA* conferring resistance to mupirocin | Antibiotic target alteration | 24.8 | 76 | ≤ 1e-5 |
| *ACXA17_00065* | *Erm(52)* | Antibiotic target alteration | 26 | 67.9 | ≤ 1e-5 |
| *ACXA17_02155* | *Clostridioides difficile gyrB* conferring resistance to fluoroquinolones | Antibiotic target alteration | 56.8 | 98 | ≤ 1e-5 |
| *ACXA17_02465* | *Mycobacterium tuberculosis gidB* mutation conferring resistance to streptomycin | Antibiotic target alteration | 28.2 | 52.6 | ≤ 1e-5 |
| *ACXA17_04045* | *Mycobacterium tuberculosis thyA* with mutation conferring resistance to para-aminosalicylic acid | Antibiotic target alteration | 65.5 | 99.6 | ≤ 1e-5 |
| *ACXA17_08075* | *Staphylococcus aureus fusA* with mutation conferring resistance to fusidic acid | Antibiotic target alteration | 53.6 | 99.3 | ≤ 1e-5 |
| *ACXA17_10585* | TxR | Antibiotic efflux | 44.9 | 59.1 | ≤ 1e-5 |
| *ACXA17_07065* | *Neisseria gonorrhoeae PBP1* conferring resistance to beta-lactam Antibiotics | Antibiotic target alteration | 29.9 | 86.2 | ≤ 1e-5 |
| *ACXA17_00330* | *vanS* gene in *vanL* cluster | Antibiotic target alteration | 28.1 | 47.2 | ≤ 1e-5 |
| *ACXA17_06305* | *Clostridioides difficile murG* with mutation conferring resistance to vancomycin | Antibiotic target alteration | 28.1 | 82.4 | ≤ 1e-5 |
| *ACXA17_06880* | *Borreliella burgdorferi murA* with mutation conferring resistance to fosfomycin | Antibiotic target alteration | 48.1 | 99.1 | ≤ 1e-5 |
| *ACXA17_05400* | *Agrobacterium fabrum* chloramphenicol acetyltransferase | Antibiotic inactivation | 39.6 | 28.4 | ≤ 1e-5 |
| *ACXA17_04080* | *LpxC* | Antibiotic target alteration | 29.1 | 63.4 | ≤ 1e-5 |
| *ACXA17_01985* | *Mycobacterium tuberculosis rpsA* mutations conferring resistance to pyrazinamide | Antibiotic target alteration | 38.6 | 55.1 | ≤ 1e-5 |
| *ACXA17_05580* | *Staphylococcus aureus fusE* with mutation conferring resistance to fusidic acid | Antibiotic target alteration | 48.9 | 98.9 | ≤ 1e-5 |
| *ACXA17_06870* | *vanR* gene in *vanL* cluster | Antibiotic target alteration | 29.2 | 90.8 | ≤ 1e-5 |
| *ACXA17_08330* | *vatF* | Antibiotic inactivation | 30.4 | 31.4 | ≤ 1e-5 |
| *ACXA17_00150* | *Helicobacter pylori pbp1* mutants conferring resistance to amoxicillin | Antibiotic target alteration | 41.7 | 66.8 | ≤ 1e-5 |
| *ACXA17_05445* | *Escherichia coli fabI* mutations conferring resistance to isoniazid and triclosan | Antibiotic target alteration | 28.7 | 88 | ≤ 1e-5 |
| *ACXA17_09465* | *vanS* gene in *vanG* cluster | Antibiotic target alteration | 29.5 | 69.4 | ≤ 1e-5 |
| *ACXA17_09610* | *CRP* | Antibiotic efflux | 25.1 | 83.2 | ≤ 1e-5 |
| *ACXA17_00655* | *vanH* gene in *vanF* cluster | Antibiotic target alteration | 31.3 | 65.9 | ≤ 1e-5 |
| *ACXA17_04160* | *arnA* | Antibiotic target alteration | 31.6 | 83 | ≤ 1e-5 |
| *ACXA17_01325* | *Clostridioides difficile gyrB* conferring resistance to fluoroquinolones | Antibiotic target alteration | 33.9 | 96.9 | ≤ 1e-5 |
| *ACXA17_01860* | *Streptomyces rimosus otr(A)* | Antibiotic target protection | 29.3 | 48.3 | ≤ 1e-5 |
| *ACXA17_08490* | *msbA* | Antibiotic efflux | 36.3 | 84.8 | ≤ 1e-5 |
| *ACXA17_02320* | *Mycobacterium tuberculosis pncA* mutations conferring resistance to pyrazinamide | Antibiotic target alteration | 37.6 | 82.1 | ≤ 1e-5 |
| *ACXA17_06130* | *mlaF* | Antibiotic efflux | 24.6 | 83.9 | ≤ 1e-5 |
| *ACXA17_09375* | *msbA* | Antibiotic efflux | 34.6 | 87.3 | ≤ 1e-5 |
| *ACXA17_04580* | *optrA* | Antibiotic target protection | 33.6 | 98.3 | ≤ 1e-5 |
| *ACXA17_08750* | *Helicobacter pylori rpoB* mutation conferring resistance to rifampicin | Antibiotic target alteration or replacement | 45 | 98.4 | ≤ 1e-5 |
| *ACXA17_09100* | *vanH* gene in *vanA* cluster | Antibiotic target alteration | 33.5 | 85.2 | ≤ 1e-5 |
| *ACXA17_06455* | *Streptococcus mitis CdsA* with mutation conferring daptomycin resistance | Antibiotic target alteration | 36.7 | 53.1 | ≤ 1e-5 |
| *ACXA17_03185* | *ykkD* | Antibiotic efflux | 39.4 | 98.1 | ≤ 1e-5 |
| *ACXA17_05995* | *Mycobacterium tuberculosis kasA* mutant conferring resistance to isoniazid | Antibiotic target alteration | 38.2 | 98.3 | ≤ 1e-5 |
| *ACXA17_01770* | *tetB(46)* | Antibiotic efflux | 31.8 | 63.1 | ≤ 1e-5 |
| *ACXA17_04740* | *DfrA38* | Antibiotic target replacement | 42.3 | 95.7 | ≤ 1e-5 |
| *ACXA17_06675* | *oqxA* | Antibiotic efflux | 31.6 | 79.7 | ≤ 1e-5 |
| *ACXA17_03455* | *macB* | Antibiotic efflux | 36.4 | 99.5 | ≤ 1e-5 |
| *ACXA17_02145* | *Capnocytophaga gingivalis gyrA* conferring resistance to fluoroquinolones | Antibiotic target alteration | 67.7 | 98.9 | ≤ 1e-5 |
| *ACXA17_04200* | *RanB* | Antibiotic efflux | 100 | 99.6 | ≤ 1e-5 |
| *ACXA17_09805* | *tet(Q)* | Antibiotic target protection | 97 | 99.8 | ≤ 1e-5 |
| *ACXA17_06940* | *vanT* gene in *vanG* cluster | Antibiotic target alteration | 33.6 | 43.4 | ≤ 1e-5 |
| *ACXA17_10435* | *OXA-209* | Antibiotic inactivation | 83.2 | 99.6 | ≤ 1e-5 |
| *ACXA17_06245* | *optrA* | Antibiotic target protection | 26.4 | 96.5 | ≤ 1e-5 |
| *ACXA17_06965* | *ADC-131* | Antibiotic inactivation | 25.3 | 77.9 | ≤ 1e-5 |
| *ACXA17_10440* | *tet(X4)* | Antibiotic inactivation | 94.5 | 98.4 | ≤ 1e-5 |
| *ACXA17_05655* | *Thermus thermophilus uL3* mutations conferring resistance to pleuromutilin antibiotics | Antibiotic target alteration | 52.5 | 98.1 | ≤ 1e-5 |
| *ACXA17_09460* | *vanR* gene in *vanF* cluster | Antibiotic target alteration | 39 | 98.2 | ≤ 1e-5 |
| *ACXA17_03100* | *cfr(B)* | Antibiotic target alteration | 31.1 | 91 | ≤ 1e-5 |
| *ACXA17_06175* | *mdtC* | Antibiotic efflux | 29.8 | 97.8 | ≤ 1e-5 |
| *ACXA17_06680* | *MexF* | Antibiotic efflux | 39.8 | 97.8 | ≤ 1e-5 |
| *ACXA17_06685* | *OprA* | Antibiotic efflux | 26.6 | 88.3 | ≤ 1e-5 |
| *ACXA17_06765* | *Staphylococcus aureus mupB* conferring resistance to mupirocin | Antibiotic target alteration | 37.3 | 95.8 | ≤ 1e-5 |
| *ACXA17_10420* | *MYO-1* | Antibiotic inactivation | 56.1 | 86.2 | ≤ 1e-5 |
| *ACXA17_04455* | *PmrF* | Antibiotic target alteration | 33.1 | 98.7 | ≤ 1e-5 |
| *ACXA17_10720* | *Escherichia coli fabG* mutations conferring resistance to triclosan | Antibiotic target alteration | 31 | 72.3 | ≤ 1e-5 |
| *ACXA17_08675* | *Enterococcus faecium EF-Tu* mutants conferring resistance to GE2270A | Antibiotic target alteration | 70.5 | 99.2 | ≤ 1e-5 |
| *ACXA17_10430* | *LnuH* | Antibiotic inactivation | 93.4 | 99.6 | ≤ 1e-5 |
| *ACXA17_08035* | *Mycobacterium tuberculosis katG* mutations conferring resistance to isoniazid | Antibiotic target alteration | 54.5 | 95.4 | ≤ 1e-5 |
| *ACXA17_06960* | *Escherichia coli fabG* mutations conferring resistance to triclosan | Antibiotic target alteration | 51.9 | 97.2 | ≤ 1e-5 |
| *ACXA17_10690* | *MCR-8.4* | Antibiotic target alteration | 34.5 | 85 | ≤ 1e-5 |
| *ACXA17_10580* | *abeM* | Antibiotic efflux | 32.9 | 96.6 | ≤ 1e-5 |
| *ACXA17_07655* | *Streptomyces cinnamoneus EF-Tu* mutants conferring resistance to elfamycin | Antibiotic target alteration | 28 | 21.8 | ≤ 1e-5 |
| *ACXA17_06790* | *Staphylococcus aureus fusA* with mutation conferring resistance to fusidic acid | Antibiotic target alteration | 24.9 | 63.5 | ≤ 1e-5 |
| *ACXA17_08555* | *ugd* | Antibiotic target alteration | 28.4 | 81.7 | ≤ 1e-5 |
| *ACXA17_05780* | *tetA(60)* | Antibiotic efflux | 30.2 | 98.6 | ≤ 1e-5 |
| *ACXA17_09565* | *bcrA* | Antibiotic efflux | 34.2 | 90.3 | ≤ 1e-5 |
| *ACXA17_08080* | *rpsJ* | Antibiotic target protection | 65 | 98 | ≤ 1e-5 |
| *ACXA17_04205* | *RanA* | Antibiotic efflux | 99.6 | 99.6 | ≤ 1e-5 |
| *ACXA17_07785* | *TxR* | Antibiotic efflux | 36.9 | 65.6 | ≤ 1e-5 |
| *ACXA17_09380* | *bcrA* | Antibiotic efflux | 36.7 | 92.6 | ≤ 1e-5 |
| *ACXA17_06835* | *lmrD* | Antibiotic efflux | 29.1 | 92.7 | ≤ 1e-5 |

^a^Resistance genes in the RA-FJ-C4 genome predicted by CARD.

^b^The standardized entry name assigned to an antimicrobial resistance determinant in the Antibiotic Resistance Ontology (ARO), which typically represents a specific resistance gene, mutation, mechanism, or drug class.

**Table S8 Predicted resistance genes of RA-BJ-C1**

| **Gene ID^a^** | **ARO Name^b^** | **Resistance Mechanism** | **Identity (%)** | **Coverage (%)** | **E-value** |
| --- | --- | --- | --- | --- | --- |
| *ACXA18_03925* | *optrA* | Antibiotic target protection | 33.6 | 98.3 | ≤ 1e-5 |
| *ACXA18_08115* | *msbA* | Antibiotic efflux | 36.4 | 45.2 | ≤ 1e-5 |
| *ACXA18_10050* | *ErmF* | Antibiotic target alteration | 98.5 | 99.6 | ≤ 1e-5 |
| *ACXA18_06045* | *OprA* | Antibiotic efflux | 26.6 | 88.3 | ≤ 1e-5 |
| *ACXA18_09035* | *vanR* gene in *vanF* cluster | Antibiotic target alteration | 39 | 98.2 | ≤ 1e-5 |
| *ACXA18_01335* | *Mycobacterium tuberculosis rpsA* mutations conferring resistance to pyrazinamide | Antibiotic target alteration | 38.6 | 55.1 | ≤ 1e-5 |
| *ACXA18_01045* | *Clostridioides difficile gyrB* conferring resistance to fluoroquinolones | Antibiotic target alteration | 33.9 | 96.9 | ≤ 1e-5 |
| *ACXA18_01210* | *Streptomyces rimosus otr(A)* | Antibiotic target protection | 29.3 | 48.3 | ≤ 1e-5 |
| *ACXA18_10660* | *MCR-8.4* | Antibiotic target alteration | 34.5 | 85 | ≤ 1e-5 |
| *ACXA18_10085* | *EreD* | Antibiotic inactivation | 95.3 | 99.8 | ≤ 1e-5 |
| *ACXA18_06295* | *vanT* gene in *vanG* cluster | Antibiotic target alteration | 33.6 | 43.4 | ≤ 1e-5 |
| *ACXA18_00150* | *Helicobacter pylori pbp1* mutants conferring resistance to amoxicillin | Antibiotic target alteration | 41.7 | 66.8 | ≤ 1e-5 |
| *ACXA18_01915* | *tet(36)* | Antibiotic target protection | 27.9 | 85.6 | ≤ 1e-5 |
| *ACXA18_01490* | *Capnocytophaga gingivalis gyrA* conferring resistance to fluoroquinolones | Antibiotic target alteration | 67.7 | 98.2 | ≤ 1e-5 |
| *ACXA18_01805* | *Mycobacterium tuberculosis gidB* mutation conferring resistance to streptomycin | Antibiotic target alteration | 28.2 | 52.6 | ≤ 1e-5 |
| *ACXA18_09185* | *CRP* | Antibiotic efflux | 25.1 | 83.2 | ≤ 1e-5 |
| *ACXA18_10110* | *tet(X)* | Antibiotic inactivation | 97.7 | 99.7 | ≤ 1e-5 |
| *ACXA18_06190* | *lmrD* | Antibiotic efflux | 29.1 | 92.7 | ≤ 1e-5 |
| *ACXA18_07205* | *Mycobacterium tuberculosis folC* with mutation conferring resistance to para-aminosalicylic acid | Antibiotic target alteration | 27.4 | 80.2 | ≤ 1e-5 |
| *ACXA18_10555* | *TxR* | Antibiotic efflux | 44.9 | 59.1 | ≤ 1e-5 |
| *ACXA18_06120* | *Staphylococcus aureus mupB* conferring resistance to mupirocin | Antibiotic target alteration | 37.3 | 95.8 | ≤ 1e-5 |
| *ACXA18_01370* | *adeB* | Antibiotic efflux | 23.9 | 98.4 | ≤ 1e-5 |
| *ACXA18_08295* | *Helicobacter pylori rpoB* mutation conferring resistance to rifampicin | Antibiotic target alteration or replacement | 45 | 98.4 | ≤ 1e-5 |
| *ACXA18_02035* | *tet(41)* | Antibiotic efflux | 40.8 | 89.9 | ≤ 1e-5 |
| *ACXA18_08680* | *vanE* | Antibiotic target alteration | 31.9 | 96.9 | ≤ 1e-5 |
| *ACXA18_04775* | *Escherichia coli fabI* mutations conferring resistance to isoniazid and triclosan | Antibiotic target alteration | 28.7 | 88 | ≤ 1e-5 |
| *ACXA18_04085* | *DfrA38* | Antibiotic target replacement | 42.3 | 95.7 | ≤ 1e-5 |
| *ACXA18_10060* | *OXA-209* | Antibiotic inactivation | 86.1 | 99.6 | ≤ 1e-5 |
| *ACXA18_06035* | *MexE* | Antibiotic efflux | 29.7 | 91.4 | ≤ 1e-5 |
| *ACXA18_00650* | *vanH* gene in *vanF* cluster | Antibiotic target alteration | 31.3 | 65.3 | ≤ 1e-5 |
| *ACXA18_00330* | *vanS* gene in *vanL* cluster | Antibiotic target alteration | 28.1 | 47.2 | ≤ 1e-5 |
| *ACXA18_01660* | *Mycobacterium tuberculosis pncA* mutations conferring resistance to pyrazinamide | Antibiotic target alteration | 37.6 | 82.1 | ≤ 1e-5 |
| *ACXA18_07130* | *Helicobacter pylori frxA* mutation conferring resistance to metronidazole | Antibiotic target alteration | 26.2 | 84.8 | ≤ 1e-5 |
| *ACXA18_08650* | *vanH* gene in *vanA* cluster | Antibiotic target alteration | 33.5 | 85.2 | ≤ 1e-5 |
| *ACXA18_10135* | *tet(X)* | Antibiotic inactivation | 98.2 | 99.7 | ≤ 1e-5 |
| *ACXA18_03820* | *Staphylococcus aureus mupA* conferring resistance to mupirocin | Antibiotic target alteration | 24.8 | 76 | ≤ 1e-5 |
| *ACXA18_00900* | *bcrA* | Antibiotic efflux | 32.9 | 71.8 | ≤ 1e-5 |
| *ACXA18_02540* | *macB* | Antibiotic efflux | 36.9 | 99.5 | ≤ 1e-5 |
| *ACXA18_08045* | *msbA* | Antibiotic efflux | 36.3 | 84.8 | ≤ 1e-5 |
| *ACXA18_03550* | *RanA* | Antibiotic efflux | 99.6 | 99.6 | ≤ 1e-5 |
| *ACXA18_09140* | *bcrA* | Antibiotic efflux | 34.2 | 90.3 | ≤ 1e-5 |
| *ACXA18_04725* | *Agrobacterium fabrum* chloramphenicol acetyltransferase | Antibiotic inactivation | 39.6 | 28.4 | ≤ 1e-5 |
| *ACXA18_06320* | *ADC-131* | Antibiotic inactivation | 25.3 | 77.9 | ≤ 1e-5 |
| *ACXA18_07355* | *TxR* | Antibiotic efflux | 36.9 | 65.6 | ≤ 1e-5 |
| *ACXA18_05805* | *Streptococcus mitis CdsA* with mutation conferring daptomycin resistance | Antibiotic target alteration | 36.7 | 53.1 | ≤ 1e-5 |
| *ACXA18_10070* | *arr-1* | Antibiotic inactivation | 57 | 92.6 | ≤ 1e-5 |
| *ACXA18_08310* | *TaeA* | Antibiotic efflux | 42.7 | 99.5 | ≤ 1e-5 |
| *ACXA18_08110* | *ugd* | Antibiotic target alteration | 29 | 81.7 | ≤ 1e-5 |
| *ACXA18_05500* | *mdtC* | Antibiotic efflux | 29.8 | 97.8 | ≤ 1e-5 |
| *ACXA18_04910* | *Staphylococcus aureus fusE* with mutation conferring resistance to fusidic acid | Antibiotic target alteration | 48.9 | 98.9 | ≤ 1e-5 |
| *ACXA18_10130* | *catB8* | Antibiotic inactivation | 71.5 | 98.6 | ≤ 1e-5 |
| *ACXA18_02840* | *OmpA* | Reduced permeability to antibiotic | 52.9 | 10.3 | ≤ 1e-5 |
| *ACXA18_02130* | *salE* | Antibiotic target protection | 29.3 | 61.3 | ≤ 1e-5 |
| *ACXA18_06315* | *Escherichia coli fabG* mutations conferring resistance to triclosan | Antibiotic target alteration | 51.9 | 97.2 | ≤ 1e-5 |
| *ACXA18_10125* | *DfrA36* | Antibiotic target replacement | 67.3 | 72.2 | ≤ 1e-5 |
| *ACXA18_05455* | *mlaF* | Antibiotic efflux | 24.6 | 83.9 | ≤ 1e-5 |
| *ACXA18_07225* | *Streptomyces cinnamoneus EF-Tu* mutants conferring resistance to elfamycin | Antibiotic target alteration | 28 | 21.8 | ≤ 1e-5 |
| *ACXA18_06040* | *MexF* | Antibiotic efflux | 39.8 | 97.8 | ≤ 1e-5 |
| *ACXA18_07665* | *rpsJ* | Antibiotic target protection | 65 | 98 | ≤ 1e-5 |
| *ACXA18_07650* | *Mycobacterium tuberculosis rpsL* mutations conferring resistance to Streptomycin | Antibiotic target alteration | 76.4 | 96.1 | ≤ 1e-5 |
| *ACXA18_05630* | *Clostridioides difficile murG* with mutation conferring resistance to vancomycin | Antibiotic target alteration | 28.1 | 82.4 | ≤ 1e-5 |
| *ACXA18_10550* | *abeM* | Antibiotic efflux | 32.9 | 96.6 | ≤ 1e-5 |
| *ACXA18_05320* | *Mycobacterium tuberculosis kasA* mutant conferring resistance to isoniazid | Antibiotic target alteration | 38.2 | 98.3 | ≤ 1e-5 |
| *ACXA18_05445* | *vanS* gene in *vanL* cluster | Antibiotic target alteration | 37.8 | 23.1 | ≤ 1e-5 |
| *ACXA18_07905* | *vatF* | Antibiotic inactivation | 30.4 | 31.4 | ≤ 1e-5 |
| *ACXA18_02190* | *cfr(B)* | Antibiotic target alteration | 31.1 | 91 | ≤ 1e-5 |
| *ACXA18_10095* | *LnuH* | Antibiotic inactivation | 100 | 99.6 | ≤ 1e-5 |
| *ACXA18_09040* | *vanS* gene in *vanG* cluster | Antibiotic target alteration | 29.5 | 69.4 | ≤ 1e-5 |
| *ACXA18_06145* | *Staphylococcus aureus fusA* with mutation conferring resistance to fusidic acid | Antibiotic target alteration | 24.9 | 63.5 | ≤ 1e-5 |
| *ACXA18_03505* | *arnA* | Antibiotic target alteration | 31.6 | 83 | ≤ 1e-5 |
| *ACXA18_02275* | *ykkD* | Antibiotic efflux | 39.4 | 98.1 | ≤ 1e-5 |
| *ACXA18_02565* | *Escherichia coli folP* with mutation conferring resistance to sulfonamides | Antibiotic target alteration | 38.4 | 96.7 | ≤ 1e-5 |
| *ACXA18_03390* | *Mycobacterium tuberculosis thyA* with mutation conferring resistance to para-aminosalicylic acid | Antibiotic target alteration | 65.5 | 99.6 | ≤ 1e-5 |
| *ACXA18_10690* | *Escherichia coli fabG* mutations conferring resistance to triclosan | Antibiotic target alteration | 31 | 72.3 | ≤ 1e-5 |
| *ACXA18_10080* | *OXA-209* | Antibiotic inactivation | 83.2 | 99.6 | ≤ 1e-5 |
| *ACXA18_09275* | *bcrA* | Antibiotic efflux | 34.7 | 88.4 | ≤ 1e-5 |
| *ACXA18_07490* | *Helicobacter pylori pbp2* mutants conferring resistance to amoxicillin | Antibiotic target alteration | 25.6 | 81.5 | ≤ 1e-5 |
| *ACXA18_00050* | *bacA* | Antibiotic target alteration | 40.9 | 97.8 | ≤ 1e-5 |
| *ACXA18_03545* | *RanB* | Antibiotic efflux | 100 | 99.6 | ≤ 1e-5 |
| *ACXA18_05570* | *optrA* | Antibiotic target protection | 26.4 | 96.5 | ≤ 1e-5 |
| *ACXA18_04985* | *Thermus thermophilus uL3* mutations conferring resistance to pleuromutilin antibiotics | Antibiotic target alteration | 52.5 | 98.1 | ≤ 1e-5 |
| *ACXA18_07620* | *Mycobacterium tuberculosis katG* mutations conferring resistance to isoniazid | Antibiotic target alteration | 54.5 | 95.4 | ≤ 1e-5 |
| *ACXA18_01055* | *Clostridioides difficile gyrA* conferring resistance to fluoroquinolones | Antibiotic target alteration | 29.8 | 54.8 | ≤ 1e-5 |
| *ACXA18_04150* | *PmrF* | Antibiotic target alteration | 31.6 | 65.9 | ≤ 1e-5 |
| *ACXA18_08950* | *msbA* | Antibiotic efflux | 34.6 | 87.3 | ≤ 1e-5 |
| *ACXA18_08290* | *Escherichia coli rpoB* mutants conferring resistance to rifampicin | Antibiotic target alteration or replacement | 45.6 | 98 | ≤ 1e-5 |
| *ACXA18_01500* | *Clostridioides difficile gyrB* conferring resistance to fluoroquinolones | Antibiotic target alteration | 57 | 98 | ≤ 1e-5 |
| *ACXA18_06235* | *Borreliella burgdorferi murA* with mutation conferring resistance to fosfomycin | Antibiotic target alteration | 48.1 | 99.1 | ≤ 1e-5 |
| *ACXA18_06225* | *vanR* gene in *vanL* cluster | Antibiotic target alteration | 29.2 | 90.8 | ≤ 1e-5 |
| *ACXA18_07660* | *Staphylococcus aureus fusA* with mutation conferring resistance to fusidic acid | Antibiotic target alteration | 53.6 | 99.3 | ≤ 1e-5 |
| *ACXA18_06425* | *Neisseria gonorrhoeae PBP1* conferring resistance to beta-lactam Antibiotics | Antibiotic target alteration | 29.9 | 86.2 | ≤ 1e-5 |
| *ACXA18_00065* | *Erm(52)* | Antibiotic target alteration | 26 | 67.9 | ≤ 1e-5 |
| *ACXA18_03800* | *PmrF* | Antibiotic target alteration | 33.1 | 98.7 | ≤ 1e-5 |
| *ACXA18_03425* | *LpxC* | Antibiotic target alteration | 29.1 | 63.4 | ≤ 1e-5 |
| *ACXA18_05105* | *tetA(60)* | Antibiotic efflux | 30.2 | 98.6 | ≤ 1e-5 |
| *ACXA18_08955* | *bcrA* | Antibiotic efflux | 36.7 | 92.6 | ≤ 1e-5 |
| *ACXA18_08230* | *Enterococcus faecium EF-Tu* mutants conferring resistance to GE2270A | Antibiotic target alteration | 70.5 | 99.2 | ≤ 1e-5 |

^a^Resistance genes in the RA-BJ-C1 genome predicted by CARD.

^b^The standardized entry name assigned to an antimicrobial resistance determinant in the Antibiotic Resistance Ontology (ARO), which typically represents a specific resistance gene, mutation, mechanism, or drug class.

**Table S9 Comparison of resistance genes and phenotypes in RA-FJ-C1, RA-FJ-C4, and RA-BJ-C**

| **Categories of resistance genes** | **Antimicrobials** | **Comparison of resistance genes and phenotypes** | | |
| --- | --- | --- | --- | --- |
|  |  | **RA-FJ-C1**  (**resistance gene, phenotype**) | **RA-FJ-C4**  (**resistance gene, phenotype**) | **RA-BJ-C1**  (**resistance gene, phenotype**) |
| Penam | Penicillins | Yes^a^ (+, +) | No^b^ (+, -) | No (+, -) |
|  | Ampicillin | Yes (+, +) | No (+, -) | No (+, -) |
|  | Piperacillin | No (+, -) | No (+, -) | No (+, -) |
| Cephalosporin | Cefazolin | Yes (+, +) | No (+, -) | No (+, -) |
|  | Cefuroxime | No (+, -) | No (+, -) | No (+, -) |
|  | Ceftazidime | No (+, -) | No (+, -) | No (+, -) |
|  | Ceftriaxone | No (+, -) | No (+, -) | No (+, -) |
|  | Cefoperazone | No (+, -) | No (+, -) | No (+, -) |
| Aminoglycosides | Amikacin | Yes (+, +) | Yes (+, +) | Yes (+, +) |
|  | Gentamicin | Yes (+, +) | Yes (+, +) | Yes (+, +) |
|  | Kanamycin | Yes (+, +) | Yes (+, +) | Yes (+, +) |
| Tetracyclines | Tetracycline | Yes (+, +) | Yes (+, +) | No (+, -) |
|  | Doxycycline | No (+, -) | No (+, -) | No (+, -) |
|  | Minocycline | No (+, -) | No (+, -) | No (+, -) |
| Macrolides | Erythromycin | Yes (+, +) | No (+, -) | Yes (+, +) |
| Fluoroquinolones | Norfloxacin | Yes (+, +) | Yes (+, +) | Yes (+, +) |
| Diaminopyrimidine | Trimethoprim-  sulfamethoxazole | Yes (+, +) | No (+, -) | Yes (+, +) |
| Phenicols | Chloramphenicol | No (+, -) | No (+, -) | No (+, -) |
| Peptides | Polymyxin B | Yes (+, +) | Yes (+, +) | Yes (+, +) |

"Yes", the resistance genotype is consistent with the resistance phenotype.

"No", the resistance genotype is inconsistent with the resistance phenotype.

"+", the strain carries the resistance gene or is resistant to the antimicrobial.

"-", the strain does not carry the resistance gene or is not resistant to the antimicrobial.

**Table S10 Predicted genes associated with pathogen-host interactions in RA-FJ-C1**

| **Gene ID^a^** | **Hit Gene Name^b^** | **Mutant Phenotype** |
| --- | --- | --- |
| *ACXA16_00010* | *BCAL2200* | Reduced virulence |
| *ACXA16_00050* | *ChpE (CMM_0039)* | Effector (plant avirulence determinant) |
| *ACXA16_00065* | *ksgA* | Reduced virulence |
| *ACXA16_00100* | *cob* | Unaffected pathogenicity |
| *ACXA16_00105* | *IDTR* | Reduced virulence |
| *ACXA16_00265* | *BbCypC* | Unaffected pathogenicity |
| *ACXA16_00285* | *Fox3p* | Unaffected pathogenicity |
| *ACXA16_00290* | *MoIVD* | Reduced virulence |
| *ACXA16_00375* | *oprF* | Reduced virulence |
| *ACXA16_00390* | *STM14_RS07785* | Reduced virulence |
| *ACXA16_00415* | *PSPTO_2696* | Unaffected pathogenicity |
| *ACXA16_00420* | *murF* | Unaffected pathogenicity; Loss of pathogenicity |
| *ACXA16_00470* | *KdsD* | Reduced virulence |
| *ACXA16_00475* | *tatC* | Reduced virulence |
| *ACXA16_00500* | *tsaA* | Reduced virulence |
| *ACXA16_00540* | *mnmE* | Reduced virulence |
| *ACXA16_00580* | *ScpA* | Unaffected pathogenicity |
| ACXA16_00610 | Rv3213c | Increased virulence |
| *ACXA16_00665* | *gua1* | Loss of pathogenicity |
| *ACXA16_00670* | *Myco4* | Reduced virulence |
| *ACXA16_00685* | *glcV* | Reduced virulence |
| *ACXA16_00700* | *MepS* | Reduced virulence |
| *ACXA16_00740* | *essC (lmo0061)* | Increased virulence |
| *ACXA16_00810* | *idi* | Reduced virulence |
| *ACXA16_00820* | *pgm* | Reduced virulence; Increased virulence |
| *ACXA16_00930* | *URA3* | Reduced virulence |
| *ACXA16_00935* | *pqsD* | Reduced virulence |
| *ACXA16_00940* | *ADE5* | Reduced virulence |
| *ACXA16_00945* | *purN* | Unaffected pathogenicity; Reduced virulence |
| *ACXA16_00955* | *asnB* | Unaffected pathogenicity |
| *ACXA16_00975* | *StpC* | Unaffected pathogenicity |
| *ACXA16_01000* | *FGSG_13826* | Unaffected pathogenicity |
| *ACXA16_01050* | *bglB* | Reduced virulence; Increased virulence |
| *ACXA16_01085* | *rny; cvfA* | Reduced virulence |
| *ACXA16_01110* | *GyrA* | Chemistry target: sensitivity to chemical |
| *ACXA16_01115* | *rsh* | Unaffected pathogenicity; Reduced virulence |
| *ACXA16_01120* | *nhaA* | Reduced virulence |
| *ACXA16_01125* | *gGT* | Reduced virulence |
| *ACXA16_01145* | *TRPS* | Lethal |
| *ACXA16_01155* | *trpB* | Reduced virulence |
| *ACXA16_01165* | *trpC (PXO_04004)* | Reduced virulence |
| *ACXA16_01185* | *trpA* | Reduced virulence |
| *ACXA16_01210* | *clbD* | Unaffected pathogenicity |
| *ACXA16_01230* | *purF (Dda3937_02099)* | Reduced virulence |
| *ACXA16_01245* | *GzOB034* | Rnaffected pathogenicity |
| *ACXA16_01255* | *PurA* | Reduced virulence |
| *ACXA16_01275* | *bipA* | Reduced virulence |
| *ACXA16_01300* | *anmK* | Effector (plant avirulence determinant) |
| *ACXA16_01380* | *yaaA* | Reduced virulence |
| *ACXA16_01415* | *pnp* | Reduced virulence |
| *ACXA16_01500* | *mexB* | Unaffected pathogenicity |
| *ACXA16_01565* | *cps2J* | Lethal; Reduced virulence |
| *ACXA16_01575* | *acs* | Unaffected pathogenicity; Reduced virulence |
| *ACXA16_01600* | *Lmo0866; CshA* | Unaffected pathogenicity; Increased virulence |
| *ACXA16_01625* | *GyrA* | Chemistry target: resistance or sensitivity to chemical |
| *ACXA16_01640* | *SSBX* | Reduced virulence |
| *ACXA16_01660* | *MoIVD (MGG_02540)* | Reduced virulence |
| *ACXA16_01680* | *ppc* | Unaffected pathogenicity |
| *ACXA16_01705* | *pqsH* | Reduced virulence |
| *ACXA16_01715* | *pimt* | Reduced virulence |
| *ACXA16_01790* | *tatA (A0889)* | Reduced virulence |
| *ACXA16_01850* | *tktA (FTN_1333)* | Unaffected pathogenicity |
| *ACXA16_01900* | *yedX* | Unaffected pathogenicity |
| *ACXA16_01900* | *yedX* | Unaffected pathogenicity |
| *ACXA16_01905* | *pdhB* | Reduced virulence |
| *ACXA16_01930* | *GroEL* | Increased virulence |
| *ACXA16_01980* | *galE* | Unaffected pathogenicity; Reduced virulence |
| *ACXA16_01985* | *arnB* | Unaffected pathogenicity |
| *ACXA16_02275* | *ADE13* | Reduced virulence |
| *ACXA16_02290* | *MoSCAD2 (MGG_08690)* | Loss of pathogenicity |
| *ACXA16_02295* | *Lmo0866; CshA* | Increased virulence |
| *ACXA16_02310* | *MoSCAD2(MGG_08690)* | Loss of pathogenicity |
| *ACXA16_02315* | *fadD1* | Unaffected pathogenicity |
| *ACXA16_02370* | *odhB* | Unaffected pathogenicity |
| *ACXA16_02375* | *VdOGDH (VDAG_10018)* | Reduced virulence |
| *ACXA16_02395* | *atpG* | Reduced virulence |
| *ACXA16_02400* | *ATP1* | Loss of pathogenicity |
| ACXA16_02500 | thiE (pspto_4799) | Reduced virulence |
| *ACXA16_02505* | *thiG (pspto_0434)* | Reduced virulence |
| *ACXA16_02515* | *Uba2; Mouba2* | Reduced virulence |
| *ACXA16_02520* | *VdTHI20* | Reduced virulence |
| *ACXA16_02525* | *GUS1* | Lethal |
| *ACXA16_02565* | *nit2* | Unaffected pathogenicity |
| *ACXA16_02580* | *hmuV* | Unaffected pathogenicity |
| *ACXA16_02610* | *carA (Dda3937_01390)* | Reduced virulence |
| *ACXA16_02710* | *cspC* | Unaffected pathogenicity; Reduced virulence; Loss of pathogenicity |
| *ACXA16_02720* | *RhlE2 (PA0428); RhlE1 (PA3950)* | Reduced virulence |
| *ACXA16_02765* | *CLC-A* | Reduced virulence |
| *ACXA16_02795* | *Als1; ALS1* | Reduced virulence; Loss of pathogenicity |
| *ACXA16_02805* | *cys4* | Reduced virulence |
| *ACXA16_02825* | *LpdA* | Effector (plant avirulence determinant) |
| *ACXA16_02830* | *FTT1564* | Unaffected pathogenicity |
| *ACXA16_02835* | *FTT1564* | Unaffected pathogenicity |
| *ACXA16_02850* | *thioredoxin 1* | Reduced virulence |
| *ACXA16_02965* | *Mfd* | Loss of pathogenicity |
| *ACXA16_02970* | *potA* | Reduced virulence |
| *ACXA16_03020* | *PitA* | Unaffected pathogenicity; Reduced virulence |
| *ACXA16_03035* | *MSY1* | Reduced virulence |
| *ACXA16_03090* | *radC* | Reduced virulence |
| *ACXA16_03095* | *virB (lmo1747)* | Unaffected pathogenicity; Reduced virulence |
| *ACXA16_03120* | *folP* | Unaffected pathogenicity |
| *ACXA16_03140* | *XC_2466* | Reduced virulence |
| *ACXA16_03145* | *hupA* | Reduced virulence |
| *ACXA16_03170* | *aroB* | Reduced virulence |
| *ACXA16_03190* | *lipA (SAUSA300_0829)* | Reduced virulence |
| *ACXA16_03230* | *Zmp1* | Increased virulence |
| *ACXA16_03270* | *ppk* | Reduced virulence |
| *ACXA16_03365* | *OmpA1* | Reduced virulence |
| *ACXA16_03370* | *OmpA1* | Reduced virulence |
| *ACXA16_03395* | *greA* | Loss of pathogenicity |
| *ACXA16_03405* | *ClpX* | Reduced virulence |
| *ACXA16_03435* | *SPD_1366 (CvfD)* | Reduced virulence |
| *ACXA16_03440* | *pdhB* | Reduced virulence |
| *ACXA16_03470* | *GAPDH* | Reduced virulence |
| *ACXA16_03475* | *pfkA* | Reduced virulence |
| *ACXA16_03485* | *bfpR (THC0290_1679)* | Unaffected pathogenicity; Reduced virulence |
| *ACXA16_03505* | *PA4485* | Unaffected pathogenicity |
| *ACXA16_03575* | *lpxD* | Reduced virulence |
| *ACXA16_03580* | *EF-P* | Increased virulence |
| *ACXA16_03585* | *lpxA* | Reduced virulence |
| *ACXA16_03590* | *lpxA* | Reduced virulence |
| *ACXA16_03595* | *lpxD* | Reduced virulence |
| *ACXA16_03940* | *pnp* | Reduced virulence |
| *ACXA16_03950* | *ldh-1* | Unaffected pathogenicity; Reduced virulence |
| *ACXA16_03960* | *FMT* | Reduced virulence |
| *ACXA16_03965* | *MurI* | Reduced virulence |
| *ACXA16_03995* | *gumD* | Loss of pathogenicity |
| *ACXA16_04000* | *mlaE* | Reduced virulence |
| *ACXA16_04005* | *potA* | Reduced virulence |
| *ACXA16_04025* | *cifA* | Reduced virulence |
| *ACXA16_04040* | *ADE2* | Reduced virulence |
| *ACXA16_04045* | *ADE2* | Reduced virulence; Loss of pathogenicity |
| *ACXA16_04100* | *XC_0450* | Reduced virulence |
| *ACXA16_04120* | *Rv0462 (lpdC)* | Increased virulence |
| *ACXA16_04130* | *StpC* | Unaffected pathogenicity |
| *ACXA16_04135* | *dnaJ* | Reduced virulence |
| *ACXA16_04145* | *mntH2* | Unaffected pathogenicity; Reduced virulence |
| *ACXA16_04150* | *B739_1208* | Reduced virulence |
| *ACXA16_04175* | *rpe (FTN_1221)* | Reduced virulence |
| *ACXA16_04180* | *rpoS* | Unaffected pathogenicity |
| *ACXA16_04185* | *MoHYR1; MoHyr1* | Reduced virulence |
| *ACXA16_04210* | *PGM* | Reduced virulence |
| *ACXA16_04220* | *arnC* | Unaffected pathogenicity |
| *ACXA16_04275* | *ERG10* | Lethal |
| *ACXA16_04285* | *pepP (CJJ81176_0681)* | Reduced virulence |
| *ACXA16_04345* | *lsrA* | Reduced virulence |
| *ACXA16_04350* | *gcvP* | Reduced virulence |
| *ACXA16_04365* | *dnaJ* | Reduced virulence |
| *ACXA16_04390* | *MnhA1* | Reduced virulence |
| *ACXA16_04400* | *nuoJ (PA2645)* | Unaffected pathogenicity; Reduced virulence |
| *ACXA16_04415* | *nuoG* | Unaffected pathogenicity |
| *ACXA16_04445* | *STM14_RS15275* | Unaffected pathogenicity |
| *ACXA16_04505* | *rmlA* | Reduced virulence; Loss of pathogenicity |
| *ACXA16_04510* | *USX1* | Loss of pathogenicity |
| *ACXA16_04520* | *gumD* | Loss of pathogenicity |
| *ACXA16_04525* | *pglE* | Reduced virulence |
| *ACXA16_04530* | *gigX4* | Increased virulence |
| *ACXA16_04550* | *gigX3* | Increased virulence |
| *ACXA16_04565* | *gumD* | Loss of pathogenicity |
| *ACXA16_04620* | *algD* | Reduced virulence |
| *ACXA16_04630* | *AS87_04050* | Reduced virulence |
| *ACXA16_04640* | *wza* | Reduced virulence |
| *ACXA16_04645* | *wbtA* | Reduced virulence |
| *ACXA16_04655* | *glyA* | Reduced virulence |
| *ACXA16_04660* | *pgi* | Reduced virulence |
| *ACXA16_04685* | *aroA* | Unaffected pathogenicity; Reduced virulence |
| *ACXA16_04740* | *pyrG* | Unaffected pathogenicity |
| *ACXA16_04745* | *RecA* | Reduced virulence |
| *ACXA16_04815* | *Nmo2* | Reduced virulence |
| *ACXA16_04860* | *nfu* | Reduced virulence |
| *ACXA16_05125* | *clpB* | Unaffected pathogenicity |
| *ACXA16_05155* | *MoCA1 (MGG_04611)* | Reduced virulence |
| *ACXA16_05160* | *lacA* | Reduced virulence |
| *ACXA16_05210* | *YmxG (HP0657)* | Unaffected pathogenicity; Reduced virulence |
| *ACXA16_05215* | *MFP1* | Reduced virulence |
| *ACXA16_05250* | *AmiC1 (XC_1816)* | Reduced virulence |
| *ACXA16_05270* | *Eno* | Unaffected pathogenicity |
| *ACXA16_05325* | *secY* | Increased virulence |
| *ACXA16_05375* | *rplN* | Unaffected pathogenicity; Increased virulence |
| *ACXA16_05410* | *GzOB009* | Lethal |
| *ACXA16_05460* | *yeaZ* | Reduced virulence |
| *ACXA16_05470* | *accD* | Reduced virulence |
| *ACXA16_05545* | *LmABCB3* | Reduced virulence |
| *ACXA16_05605* | *htrA; HtrA* | Reduced virulence |
| *ACXA16_05610* | *yhbU* | Unaffected pathogenicity |
| *ACXA16_05670* | *PykM (BAB1_1761); pyk* | Reduced virulence |
| *ACXA16_05680* | *rnc* | Unaffected pathogenicity; Reduced virulence |
| *ACXA16_05685* | *KSA1; PKS9* | Unaffected pathogenicity |
| *ACXA16_05690* | *Acp1 (PA1869)* | Increased virulence |
| *ACXA16_05790* | *argD* | Unaffected pathogenicity; Reduced virulence; Loss of pathogenicity |
| *ACXA16_05795* | *DUR1,2* | Reduced virulence |
| *ACXA16_05825* | *sreS* | Unaffected pathogenicity |
| *ACXA16_05835* | *VraD* | Reduced virulence |
| *ACXA16_05885* | *yegN* | Reduced virulence |
| *ACXA16_05930* | *prfA (lmo2543)* | Reduced virulence |
| *ACXA16_05945* | *OmpA2* | Reduced virulence |
| *ACXA16_05950* | *smpB* | Reduced virulence |
| *ACXA16_05955* | *cpxA* | Reduced virulence |
| *ACXA16_05990* | *VK055_1952* | Unaffected pathogenicity |
| *ACXA16_06015* | *A1479* | Reduced virulence |
| *ACXA16_06025* | *murD* | Reduced virulence; Loss of pathogenicity |
| *ACXA16_06040* | *pbpC* | Unaffected pathogenicity; Increased virulence |
| *ACXA16_06050* | *rsmH* | Reduced virulence |
| *ACXA16_06075* | *msrA2* | Unaffected pathogenicity; Reduced virulence |
| *ACXA16_06080* | *Lon* | Loss of pathogenicity |
| *ACXA16_06120* | *GacA* | Lethal |
| *ACXA16_06130* | *B739_0832 (OmpH)* | Reduced virulence |
| *ACXA16_06135* | *omp17* | Reduced virulence |
| *ACXA16_06165* | *CDS1* | Lethal |
| *ACXA16_06235* | *mlh1* | Unaffected pathogenicity |
| *ACXA16_06320* | *PGK2* | Unaffected pathogenicity |
| *ACXA16_06345* | *gidA* | Reduced virulence |
| *ACXA16_06375* | *PCK1* | Reduced virulence |
| *ACXA16_06385* | *mexE* | Increased virulence |
| *ACXA16_06390* | *mexF* | Increased virulence |
| *ACXA16_06395* | *adeK* | Unaffected pathogenicity |
| *ACXA16_06400* | *RfbB* | Reduced virulence |
| *ACXA16_06470* | *PpdaAc* | Reduced virulence |
| *ACXA16_06495* | *bipA* | Reduced virulence |
| *ACXA16_06505* | *sufB; STM14_RS07705 (sufB)* | Unaffected pathogenicity; Reduced virulence |
| *ACXA16_06535* | *STM14_RS07710 (sufC)* | Reduced virulence |
| *ACXA16_06540* | *STM14_RS07715 (sufD)* | Reduced virulence |
| *ACXA16_06565* | *srrB* | Reduced virulence; Increased virulence |
| *ACXA16_06570* | *cpxR (BFW97_22310)* | Reduced virulence |
| *ACXA16_06580* | *murA* | Unaffected pathogenicity; Reduced virulence |
| *ACXA16_06585* | *cptRNA-ipt* | Reduced virulence; Loss of pathogenicity |
| *ACXA16_06615* | *gloB* | Unaffected pathogenicity; Reduced virulence |
| *ACXA16_06645* | *murF* | Unaffected pathogenicity; Loss of pathogenicity |
| *ACXA16_06660* | *rsmI* | Reduced virulence |
| *ACXA16_06665* | *fabG1* | Lethal |
| *ACXA16_06675* | *ssdA (AFUB_010850)* | Reduced virulence; Increased virulence |
| *ACXA16_06695* | *clpB* | Reduced virulence |
| *ACXA16_06705* | *STM14_RS07725 (ynhA/sufE)* | Reduced virulence |
| *ACXA16_06725* | *Prc* | Reduced virulence |
| *ACXA16_06780* | *PG_RS04465 (PG1010)* | Reduced virulence |
| *ACXA16_06855* | *MoMca1 (MGG_04626)* | Reduced virulence |
| *ACXA16_06885* | *Oligopeptidase B* | Reduced virulence |
| *ACXA16_06925* | *cps2J* | Lethal; Reduced virulence |
| *ACXA16_06945* | *BCAM1331* | Reduced virulence |
| *ACXA16_06950* | *msh2* | Unaffected pathogenicity |
| *ACXA16_07000* | *ftsH* | Unaffected pathogenicity |
| *ACXA16_07045* | *uvrB* | Unaffected pathogenicity |
| *ACXA16_07070* | *FTT0673p/prsAp* | Unaffected pathogenicity |
| *ACXA16_07090* | *yfiB* | Unaffected pathogenicity |
| *ACXA16_07135* | *lytS* | Unaffected pathogenicity; Reduced virulence |
| *ACXA16_07190* | *sseA* | Reduced virulence |
| *ACXA16_07225* | *feoB* | Loss of pathogenicity |
| *ACXA16_07270* | *RSp0930* | Effector (plant avirulence determinant) |
| *ACXA16_07320* | *fabD* | Reduced virulence |
| *ACXA16_07420* | *fleQ* | Unaffected pathogenicity; Increased virulence; Reduced virulence; Loss of pathogenicity |
| *ACXA16_07490* | *AS87_04050* | Reduced virulence |
| *ACXA16_07500* | *secA* | Reduced virulence |
| *ACXA16_07510* | *hmuR* | Unaffected pathogenicity |
| *ACXA16_07525* | *mnmE* | Reduced virulence |
| *ACXA16_07580* | *ireA* | Unaffected pathogenicity |
| *ACXA16_07660* | *fumA* | Reduced virulence |
| *ACXA16_07685* | *STM14_RS07720 (sufS)* | Reduced virulence |
| *ACXA16_07705* | *katG* | Increased virulence |
| *ACXA16_07750* | *GzOB039* | Unaffected pathogenicity |
| *ACXA16_07815* | *hemH* | Unaffected pathogenicity |
| *ACXA16_07905* | *FgEch1 (FGSG_13111)* | Reduced virulence |
| *ACXA16_07915* | *katA* | Unaffected pathogenicity |
| *ACXA16_07920* | *SoxR* | Reduced virulence |
| *ACXA16_07925* | *MGG_00383* | Reduced virulence |
| *ACXA16_07930* | *sodA1* | Reduced virulence |
| *ACXA16_07960* | *VK055_1961* | Unaffected pathogenicity |
| *ACXA16_07975* | *ABC3* | Loss of pathogenicity |
| *ACXA16_07985* | *cps2J* | Lethal; Reduced virulence |
| *ACXA16_08010* | *lacA* | Reduced virulence |
| *ACXA16_08075* | *RIB1 (CAGL0F04279g)* | Reduced virulence |
| *ACXA16_08080* | *B9H01_RS05605* | Reduced virulence |
| *ACXA16_08110* | *CBU_0265* | Unaffected pathogenicity |
| *ACXA16_08150* | *eutD* | Reduced virulence |
| *ACXA16_08170* | *IMPDH* | Reduced virulence |
| *ACXA16_08175* | *LmABCB3* | Reduced virulence |
| *ACXA16_08205* | *Rho* | Reduced virulence |
| *ACXA16_08255* | *UGD1* | Loss of pathogenicity |
| *ACXA16_08270* | *ABC3* | Loss of pathogenicity |
| *ACXA16_08285* | *A0396* | Unaffected pathogenicity |
| *ACXA16_08300* | *CcoN2 (PA14_44340)* | Unaffected pathogenicity; Reduced virulence |
| *ACXA16_08310* | *copA* | Reduced virulence |
| *ACXA16_08380* | *PYR4 (XM_003719109)* | Reduced virulence |
| *ACXA16_08385* | *tufA* | Unaffected pathogenicity; Increased virulence |
| *ACXA16_08420* | *Rv0652 (rplL)* | Increased virulence |
| *ACXA16_08450* | *RpoB* | Reduced virulence |
| *ACXA16_08455* | *FgRbp1* | Reduced virulence |
| *ACXA16_08470* | *VraD* | Reduced virulence |
| *ACXA16_08485* | *paaB* | Reduced virulence |
| *ACXA16_08595* | *RpoN* | Reduced virulence |
| *ACXA16_08600* | *GzOB046* | Unaffected pathogenicity |
| *ACXA16_08645* | *PrsA2; prsA2* | Reduced virulence |
| *ACXA16_08665* | *himD* | Reduced virulence |
| *ACXA16_08670* | *RNase E* | Reduced virulence |
| *ACXA16_08710* | *PSPTO_2691* | Unaffected pathogenicity |
| *ACXA16_08760* | *PG0343* | Reduced virulence |
| *ACXA16_08810* | *pdh* | Reduced virulence |
| *ACXA16_08845* | *ypo2062* | Reduced virulence |
| *ACXA16_08885* | *adeN* | Increased virulence |
| *ACXA16_08900* | *tbdR1* | Reduced virulence |
| *ACXA16_08950* | *Fur* | Reduced virulence |
| *ACXA16_08960* | *argD* | Unaffected pathogenicity; Reduced virulence; Loss of pathogenicity |
| *ACXA16_09010* | *mecA* | Unaffected pathogenicity; Reduced virulence |
| *ACXA16_09035* | *iroC* | Reduced virulence |
| *ACXA16_09040* | *znuC* | Reduced virulence |
| *ACXA16_09065* | *Pbl1* | Unaffected pathogenicity |
| *ACXA16_09080* | *glmS* | Reduced virulence |
| *ACXA16_09085* | *GldK (AS87_08785)* | Reduced virulence |
| *ACXA16_09095* | *gldM (AS87_RS08465)* | Reduced virulence |
| *ACXA16_09100* | *gldN* | Reduced virulence |
| *ACXA16_09120* | *phoB* | Reduced virulence |
| *ACXA16_09125* | *walK* | Reduced virulence |
| *ACXA16_09135* | *Mfd* | Loss of pathogenicity |
| *ACXA16_09165* | *aroE* | Unaffected pathogenicity; Increased virulence |
| *ACXA16_09185* | *srrA* | Reduced virulence; Increased virulence |
| *ACXA16_09195* | *rpoE* | Reduced virulence |
| *ACXA16_09205* | *Bgp (BB_0588)* | Reduced virulence |
| *ACXA16_09220* | *ABC4* | Reduced virulence; Loss of pathogenicity |
| *ACXA16_09300* | *FGSG_07816* | Unaffected pathogenicity |
| *ACXA16_09350* | *ybdB* | Lethal |
| *ACXA16_09365* | *StpC* | Unaffected pathogenicity |
| *ACXA16_09405* | *ribC* | Unaffected pathogenicity |
| *ACXA16_09410* | *Flil* | Reduced virulence |
| *ACXA16_09420* | *XC_2203* | Reduced virulence |
| *ACXA16_09425* | *Fgbat1/2 (FGSG_09512)* | Unaffected pathogenicity |
| *ACXA16_09480* | *XC_0892* | Reduced virulence |
| *ACXA16_09485* | *DPPIV* | Reduced virulence |
| *ACXA16_09575* | *odhB* | Unaffected pathogenicity |
| *ACXA16_09580* | *pdh-e1alpha* | Unaffected pathogenicity |
| *ACXA16_09595* | *bcaP* | Unaffected pathogenicity; Reduced virulence |
| *ACXA16_09755* | *aroB* | Reduced virulence |
| *ACXA16_09775* | *AlgR; algR* | Unaffected pathogenicity; Reduced virulence |
| *ACXA16_09800* | *MET13* | Loss of pathogenicity |
| *ACXA16_09810* | *AS87_RS09170 (bioF)* | Reduced virulence; Increased virulence |
| *ACXA16_09935* | *znuC* | Reduced virulence |
| *ACXA16_09980* | *Rv1988* | Reduced virulence |
| *ACXA16_10000* | *dfr1* | Reduced virulence |
| *ACXA16_10005* | *GzZC087* | Reduced virulence |
| *ACXA16_10125* | *mazF* | Unaffected pathogenicity; Increased virulence |
| *ACXA16_10125* | *mazF* | Unaffected pathogenicity; Increased virulence |
| *ACXA16_10290* | *RovA* | Reduced virulence |
| *ACXA16_10525* | *ClpP* | Reduced virulence |
| *ACXA16_10535* | *dbcA(bglu_1g06460)* | Reduced virulence |
| *ACXA16_10540* | *prfA (lmo2543)* | Reduced virulence |
| *ACXA16_10550* | *PXO_RS06935; pilR (PXO_04881)* | Unaffected pathogenicity; Reduced virulence |
| *ACXA16_10560* | *VdP4-4 (VDAG_00595)* | Reduced virulence |
| *ACXA16_10575* | *GzRad003* | Unaffected pathogenicity |
| *ACXA16_10635* | *XC_3129* | Reduced virulence |
| *ACXA16_10665* | *fabG1* | Lethal |
| *ACXA16_10670* | *STM14_1002* | Reduced virulence |
| *ACXA16_10675* | *XC_3615* | Reduced virulence |
| *ACXA16_10760* | *TRR1* | Lethal |

^a^Predicted genes associated with pathogen–host interactions in RA-FJ-C1.

^b^Homologous genes matched by BLAST alignment in the PHI database.

**Table S11 Predicted genes associated with pathogen–host interactions in RA-FJ-C4**

| **Gene ID^a^** | **Hit Gene Name^b^** | **Mutant Phenotype** |
| --- | --- | --- |
| *ACXA17_00010* | *BCAL2200* | Reduced virulence |
| *ACXA17_00050* | *ChpE (CMM_0039)* | Effector (plant avirulence determinant) |
| *ACXA17_00065* | *ksgA* | Reduced virulence |
| *ACXA17_00095* | *cob* | Unaffected pathogenicity |
| *ACXA17_00100* | *IDTR* | Reduced virulence |
| *ACXA17_00205* | *BbCypC* | Unaffected pathogenicity |
| *ACXA17_00225* | *Fox3p* | Unaffected pathogenicity |
| *ACXA17_00230* | *MoIVD (MGG_02540)* | Reduced virulence |
| *ACXA17_00315* | *oprF* | Reduced virulence |
| *ACXA17_00330* | *STM14_RS07785 (ttrS)* | Reduced virulence |
| *ACXA17_00355* | *PSPTO_2696* | Unaffected pathogenicity |
| *ACXA17_00360* | *murF* | Unaffected pathogenicity; Loss of pathogenicity |
| *ACXA17_00410* | *KdsD* | Reduced virulence |
| *ACXA17_00415* | *tatC* | Reduced virulence |
| *ACXA17_00435* | *tsaA* | Reduced virulence |
| *ACXA17_00465* | *mnmE* | Reduced virulence |
| *ACXA17_00510* | *ScpA* | Unaffected pathogenicity |
| *ACXA17_00540* | *Rv3213c* | Increased virulence |
| *ACXA17_00565* | *essC (lmo0061)* | Increased virulence |
| *ACXA17_00605* | *MepS* | Reduced virulence |
| *ACXA17_00635* | *Myco4* | Reduced virulence |
| *ACXA17_00640* | *gua1* | Loss of pathogenicity |
| *ACXA17_00705* | *idi* | Reduced virulence |
| *ACXA17_00715* | *pgm* | Reduced virulence; Increased virulence |
| *ACXA17_00795* | *cvfB* | Reduced virulence |
| *ACXA17_00860* | *URA3* | Reduced virulence |
| *ACXA17_00865* | *pqsD* | Reduced virulence |
| *ACXA17_00870* | *ADE5* | Reduced virulence |
| *ACXA17_00875* | *purN* | Unaffected pathogenicity; Reduced virulence |
| *ACXA17_00885* | *asnB* | Unaffected pathogenicity |
| *ACXA17_00895* | *StpC* | Unaffected pathogenicity |
| *ACXA17_01195* | *FGSG_13826* | Unaffected pathogenicity |
| *ACXA17_01270* | *bglB* | Reduced virulence; Increased virulence |
| *ACXA17_01310* | *rny; cvfA* | Reduced virulence |
| *ACXA17_01335* | *GyrA* | Chemistry target: sensitivity to chemical |
| *ACXA17_01340* | *rsh* | Unaffected pathogenicity; Reduced virulence |
| *ACXA17_01345* | *nhaA* | Reduced virulence |
| *ACXA17_01350* | *gGT* | Reduced virulence |
| *ACXA17_01365* | *TRPS* | Lethal |
| *ACXA17_01375* | *trpB* | Reduced virulence |
| *ACXA17_01385* | *trpC (PXO_04004)* | Reduced virulence |
| *ACXA17_01405* | *trpA* | Reduced virulence |
| *ACXA17_01430* | *clbD* | Unaffected pathogenicity |
| *ACXA17_01450* | *purF (Dda3937_02099)* | Reduced virulence |
| *ACXA17_01455* | *GzOB034* | Unaffected pathogenicity |
| *ACXA17_01465* | *PurA* | Reduced virulence |
| *ACXA17_01770* | *raxB* | Increased virulence |
| *ACXA17_01860* | *bipA* | Reduced virulence |
| *ACXA17_01945* | *yaaA* | Reduced virulence |
| *ACXA17_01985* | *pnp* | Reduced virulence |
| *ACXA17_02020* | *mexB* | Unaffected pathogenicity |
| *ACXA17_02085* | *cps2J* | Lethal; Reduced virulence |
| *ACXA17_02095* | *acs* | Unaffected pathogenicity; Reduced virulence |
| *ACXA17_02120* | *Lmo0866; CshA* | Unaffected pathogenicity; Increased virulence |
| *ACXA17_02145* | *GyrA* | Chemistry target: resistance to chemical; Chemistry target: sensitivity to chemical |
| *ACXA17_02160* | *SSBX* | Reduced virulence |
| *ACXA17_02190* | *MoIVD (MGG_02540)* | Reduced virulence |
| *ACXA17_02210* | *ppc* | Unaffected pathogenicity |
| *ACXA17_02235* | *pimt* | Reduced virulence |
| *ACXA17_02310* | *SpxA2* | Unaffected pathogenicity; Reduced virulence; Increased virulence |
| *ACXA17_02370* | *tktA (FTN_1333)* | Unaffected pathogenicity |
| *ACXA17_02400* | *yedX* | Unaffected pathogenicity |
| *ACXA17_02405* | *pdhB* | Reduced virulence |
| *ACXA17_02425* | *GroEL* | Increased virulence |
| *ACXA17_02475* | *galE* | Unaffected pathogenicity; Reduced virulence |
| *ACXA17_02480* | *arnB* | Unaffected pathogenicity |
| *ACXA17_02520* | *ADE13* | Reduced virulence |
| *ACXA17_02540* | *fabG1* | Lethal |
| *ACXA17_02550* | *MoSCAD2 (MGG_08690)* | Loss of pathogenicity |
| *ACXA17_02555* | *Lmo0866; CshA* | Unaffected pathogenicity; Increased virulence |
| *ACXA17_02565* | *MoSCAD2 (MGG_08690)* | Loss of pathogenicity |
| *ACXA17_02570* | *fadD2* | Reduced virulence |
| *ACXA17_02640* | *odhB* | Unaffected pathogenicity |
| *ACXA17_02645* | *VdOGDH (VDAG_10018)* | Reduced virulence |
| *ACXA17_02665* | *atpG* | Reduced virulence |
| *ACXA17_02670* | *ATP1* | Loss of pathogenicity |
| *ACXA17_02770* | *thiE (pspto_4799)* | Reduced virulence |
| *ACXA17_02775* | *thiG (pspto_0434)* | Reduced virulence |
| *ACXA17_02785* | *Uba2; Mouba2* | Reduced virulence |
| *ACXA17_02790* | *VdTHI20* | Reduced virulence |
| *ACXA17_02795* | *GUS1* | Lethal |
| *ACXA17_02815* | *nit2* | Unaffected pathogenicity |
| *ACXA17_02830* | *hmuV* | Unaffected pathogenicity |
| *ACXA17_02855* | *carA (Dda3937_01390)* | Reduced virulence |
| *ACXA17_03085* | *cspC* | Unaffected pathogenicity; Reduced virulence; Loss of pathogenicity |
| *ACXA17_03095* | *RhlE2 (PA0428); RhlE1 (PA3950)* | Reduced virulence |
| *ACXA17_03145* | *CLC-A* | Reduced virulence |
| *ACXA17_03165* | *Als1; ALS1* | Reduced virulence; Loss of pathogenicity |
| *ACXA17_03180* | *cys4* | Reduced virulence |
| *ACXA17_03200* | *LpdA* | Effector (plant avirulence determinant) |
| *ACXA17_03205* | *FTT1564* | Unaffected pathogenicity |
| *ACXA17_03210* | *FTT1564* | Unaffected pathogenicity |
| *ACXA17_03225* | *thioredoxin 1* | Reduced virulence |
| *ACXA17_03325* | *Mfd* | Loss of pathogenicity |
| *ACXA17_03335* | *potA* | Reduced virulence |
| *ACXA17_03385* | *PitA* | Unaffected pathogenicity; Reduced virulence |
| *ACXA17_03400* | *MSY1* | Reduced virulence |
| *ACXA17_03450* | *radC* | Reduced virulence |
| *ACXA17_03455* | *virB (lmo1747)* | Unaffected pathogenicity; Reduced virulence |
| *ACXA17_03480* | *folP* | Unaffected pathogenicity |
| *ACXA17_03500* | *XC_2466* | Reduced virulence |
| *ACXA17_03505* | *hupA* | Reduced virulence |
| *ACXA17_03610* | *aroB* | Reduced virulence |
| *ACXA17_03640* | *lipA (SAUSA300_0829)* | Reduced virulence |
| *ACXA17_03680* | *Zmp1* | Increased virulence |
| *ACXA17_03725* | *ireA* | Unaffected pathogenicity |
| *ACXA17_03740* | *ppk* | Reduced virulence |
| *ACXA17_03830* | *OmpA1* | Reduced virulence |
| *ACXA17_03835* | *OmpA1* | Reduced virulence |
| *ACXA17_03860* | *greA* | Loss of pathogenicity |
| *ACXA17_03870* | *ClpX* | Reduced virulence |
| *ACXA17_03900* | *SPD_1366 (CvfD)* | Reduced virulence |
| *ACXA17_03905* | *pdhB* | Reduced virulence |
| *ACXA17_03940* | *GAPDH* | Reduced virulence |
| *ACXA17_03945* | *pfkA* | Reduced virulence |
| *ACXA17_03955* | *bfpR (THC0290_1679)* | Unaffected pathogenicity; Reduced virulence |
| *ACXA17_03975* | *PA4485* | Unaffected pathogenicity |
| *ACXA17_04065* | *lpxD1* | Loss of pathogenicity |
| *ACXA17_04070* | *EF-P* | Increased virulence |
| *ACXA17_04075* | *lpxA* | Reduced virulence |
| *ACXA17_04080* | *lpxA* | Reduced virulence |
| *ACXA17_04085* | *lpxD* | Reduced virulence |
| *ACXA17_04140* | *pnp* | Reduced virulence |
| *ACXA17_04150* | *ldh-1* | Unaffected pathogenicity; Reduced virulence |
| *ACXA17_04160* | *FMT* | Reduced virulence |
| *ACXA17_04165* | *MurI* | Reduced virulence |
| *ACXA17_04195* | *gumD* | Loss of pathogenicity |
| *ACXA17_04200* | *mlaE* | Reduced virulence |
| *ACXA17_04205* | *proV* | Reduced virulence |
| *ACXA17_04225* | *ydiD* | Reduced virulence |
| *ACXA17_04240* | *ADE2* | Reduced virulence |
| *ACXA17_04245* | *ADE2* | Reduced virulence; Loss of pathogenicity |
| *ACXA17_04310* | *XC_0450* | Reduced virulence |
| *ACXA17_04355* | *Rv0462 (lpdC)* | Increased virulence |
| *ACXA17_04365* | *StpC* | Unaffected pathogenicity |
| *ACXA17_04370* | *dnaJ* | Reduced virulence |
| *ACXA17_04380* | *mntH2* | Unaffected pathogenicity; Reduced virulence |
| *ACXA17_04385* | *B739_1208* | Reduced virulence |
| *ACXA17_04410* | *rpe (FTN_1221)* | Reduced virulence |
| *ACXA17_04415* | *rpoS* | Unaffected pathogenicity |
| *ACXA17_04420* | *MoHYR1; MoHyr1* | Reduced virulence |
| *ACXA17_04445* | *PGM* | Reduced virulence |
| *ACXA17_04455* | *arnC* | Unaffected pathogenicity |
| *ACXA17_04510* | *ERG10* | Lethal |
| *ACXA17_04520* | *pepP (CJJ81176_0681)* | Reduced virulence |
| *ACXA17_04580* | *lsrA* | Reduced virulence |
| *ACXA17_04585* | *gcvP* | Reduced virulence |
| *ACXA17_04605* | *dnaJ* | Reduced virulence |
| *ACXA17_04630* | *MnhA1* | Reduced virulence |
| *ACXA17_04640* | *nuoJ (PA2645)* | Unaffected pathogenicity; Reduced virulence |
| *ACXA17_04655* | *nuoG* | Unaffected pathogenicity |
| *ACXA17_04685* | *STM14_RS15275* | Unaffected pathogenicity |
| *ACXA17_04750* | *rmlA* | Reduced virulence; Loss of pathogenicity |
| *ACXA17_04760* | *arnB* | Unaffected pathogenicity |
| *ACXA17_04775* | *USX1* | Loss of pathogenicity |
| *ACXA17_04785* | *gumD* | Loss of pathogenicity |
| *ACXA17_04790* | *pglE* | Reduced virulence |
| *ACXA17_04795* | *gigX6* | Increased virulence |
| *ACXA17_04800* | *gumD* | Loss of pathogenicity |
| *ACXA17_04845* | *CapD* | Unaffected pathogenicity |
| *ACXA17_04855* | *epsC* | Reduced virulence |
| *ACXA17_04860* | *algD* | Reduced virulence |
| *ACXA17_04870* | *AS87_04050* | Reduced virulence |
| *ACXA17_04880* | *wza* | Reduced virulence |
| *ACXA17_04845* | *wbtA* | Reduced virulence |
| *ACXA17_04895* | *glyA* | Reduced virulence |
| *ACXA17_04900* | *pgi* | Reduced virulence |
| *ACXA17_04930* | *aroA* | Unaffected pathogenicity; Reduced virulence |
| *ACXA17_04985* | *pyrG* | Unaffected pathogenicity |
| *ACXA17_04990* | *RecA* | Reduced virulence |
| *ACXA17_05060* | *Nmo2* | Reduced virulence |
| *ACXA17_05105* | *nfu* | Reduced virulence |
| *ACXA17_05365* | *clpB* | Unaffected pathogenicity |
| *ACXA17_05395* | *MoCA1 (MGG_04611)* | Reduced virulence |
| *ACXA17_05400* | *lacA* | Reduced virulence |
| *ACXA17_05445* | *MFP1* | Reduced virulence |
| *ACXA17_05480* | *AmiC1 (XC_1816)* | Reduced virulence |
| *ACXA17_05500* | *Eno* | Unaffected pathogenicity |
| *ACXA17_05555* | *secY* | Increased virulence |
| *ACXA17_05605* | *rplN* | Unaffected pathogenicity; Increased virulence |
| *ACXA17_05640* | *GzOB009* | Lethal |
| *ACXA17_05690* | *yeaZ* | Reduced virulence |
| *ACXA17_05700* | *accD* | Reduced virulence |
| *ACXA17_05780* | *LmABCB3* | Reduced virulence |
| *ACXA17_05835* | *cps2J* | Lethal; Reduced virulence |
| *ACXA17_05840* | *degQ (Dda3937_00697)* | Reduced virulence |
| *ACXA17_05845* | *yhbU* | Unaffected pathogenicity |
| *ACXA17_05980* | *PykM (BAB1_1761); pyk* | Reduced virulence |
| *ACXA17_05990* | *rnc* | Unaffected pathogenicity; Reduced virulence |
| *ACXA17_05995* | *KSA1; PKS9* | Unaffected pathogenicity |
| *ACXA17_06000* | *Acp1 (PA1869)* | Increased virulence |
| *ACXA17_06065* | *HP0102* | Reduced virulence |
| *ACXA17_06085* | *argD* | Unaffected pathogenicity; Reduced virulence; Loss of pathogenicity |
| *ACXA17_06090* | *DUR1,2* | Reduced virulence |
| *ACXA17_06120* | *sreS* | Unaffected pathogenicity |
| *ACXA17_06130* | *VraD* | Reduced virulence |
| *ACXA17_06175* | *yegN* | Reduced virulence |
| *ACXA17_06220* | *prfA (lmo2543)* | Reduced virulence |
| *ACXA17_06235* | *MopB* | Reduced virulence |
| *ACXA17_06240* | *smpB* | Reduced virulence |
| *ACXA17_06245* | *cpxA* | Reduced virulence |
| *ACXA17_06280* | *VK055_1952* | Unaffected pathogenicity |
| *ACXA17_06305* | *A1479* | Reduced virulence |
| *ACXA17_06315* | *murD* | Reduced virulence; Loss of pathogenicity |
| *ACXA17_06330* | *pbpC* | Unaffected pathogenicity; Increased virulence |
| *ACXA17_06340* | *rsmH* | Reduced virulence |
| *ACXA17_06365* | *msrA2* | Unaffected pathogenicity; Reduced virulence |
| *ACXA17_06370* | *Lon* | Loss of pathogenicity |
| *ACXA17_06410* | *GacA* | Lethal |
| *ACXA17_06420* | *B739_0832 (OmpH)* | Reduced virulence |
| *ACXA17_06425* | *omp17* | Reduced virulence |
| *ACXA17_06455* | *CDS1* | Lethal |
| *ACXA17_06585* | *PGK2* | Unaffected pathogenicity |
| *ACXA17_06620* | *gidA* | Reduced virulence |
| *ACXA17_06665* | *PCK1* | Reduced virulence |
| *ACXA17_06675* | *mexE* | Increased virulence |
| *ACXA17_06680* | *mexF* | Increased virulence |
| *ACXA17_06685* | *adeK* | Unaffected pathogenicity |
| *ACXA17_06690* | *RfbB* | Reduced virulence |
| *ACXA17_06760* | *PpdaAc* | Reduced virulence |
| *ACXA17_06785* | *cps2J* | Lethal; Reduced virulence |
| *ACXA17_06790* | *bipA* | Reduced virulence |
| *ACXA17_06805* | *sufB; STM14_RS07705 (sufB)* | Unaffected pathogenicity; Reduced virulence |
| *ACXA17_06835* | *STM14_RS07710 (sufC)* | Reduced virulence |
| *ACXA17_06840* | *STM14_RS07715 (sufD)* | Reduced virulence |
| *ACXA17_06865* | *srrB* | Reduced virulence; Increased virulence |
| *ACXA17_06870* | *cpxR (BFW97_22310)* | Reduced virulence |
| *ACXA17_06880* | *murA* | Unaffected pathogenicity; Reduced virulence |
| *ACXA17_06885* | *cptRNA-ipt* | Reduced virulence; Loss of pathogenicity |
| *ACXA17_06920* | *gloB* | Unaffected pathogenicity; Reduced virulence |
| *ACXA17_06940* | *murF* | Unaffected pathogenicity; Loss of pathogenicity |
| *ACXA17_00015* | *rsmI* | Reduced virulence |
| *ACXA17_06960* | *fabG1* | Lethal |
| *ACXA17_06965* | *pbpX* | Reduced virulence |
| *ACXA17_06980* | *ssdA (AFUB_010850)* | Reduced virulence; Increased virulence |
| *ACXA17_06995* | *clpB* | Reduced virulence |
| *ACXA17_07005* | *STM14_RS07725 (ynhA/sufE)* | Reduced virulence |
| *ACXA17_07025* | *Prc* | Reduced virulence |
| *ACXA17_07080* | *PG_RS04465 (PG1010)* | Reduced virulence |
| *ACXA17_07140* | *MoMca1 (MGG_04626)* | Reduced virulence |
| *ACXA17_07170* | *Oligopeptidase B* | Reduced virulence |
| *ACXA17_07210* | *cps2J* | Lethal; Reduced virulence |
| *ACXA17_07230* | *BCAM1331* | Reduced virulence |
| *ACXA17_07235* | *msh2* | Unaffected pathogenicity |
| *ACXA17_07270* | *ftsH* | Unaffected pathogenicity |
| *ACXA17_07390* | *uvrB* | Unaffected pathogenicity |
| *ACXA17_07415* | *FTT0673p/prsAp* | Unaffected pathogenicity |
| *ACXA17_07435* | *yfiB* | Unaffected pathogenicity |
| *ACXA17_07485* | *lytS* | Unaffected pathogenicity; Reduced virulence |
| *ACXA17_07530* | *sip* | Reduced virulence |
| *ACXA17_07590* | *feoB* | Loss of pathogenicity |
| *ACXA17_07635* | *RSp0930* | Effector (plant avirulence determinant) |
| *ACXA17_07685* | *fabD* | Reduced virulence |
| *ACXA17_07785* | *fleQ* | Unaffected pathogenicity; Increased virulence; Reduced virulence; Loss of pathogenicity |
| *ACXA17_07850* | *AS87_04050* | Reduced virulence |
| *ACXA17_07860* | *secA* | Reduced virulence |
| *ACXA17_07870* | *hmuR* | Unaffected pathogenicity |
| *ACXA17_07885* | *mnmE* | Reduced virulence |
| *ACXA17_07940* | *ireA* | Unaffected pathogenicity |
| *ACXA17_07995* | *fumA* | Reduced virulence |
| *ACXA17_08020* | *STM14_RS07720 (sufS)* | Reduced virulence |
| *ACXA17_08035* | *MakatG1* | Unaffected pathogenicity; Reduced virulence |
| *ACXA17_08065* | *GzOB039* | Unaffected pathogenicity |
| *ACXA17_08140* | *hemH* | Unaffected pathogenicity |
| *ACXA17_08180* | *PGIP2* | Effector (plant avirulence determinant) |
| *ACXA17_08230* | *FgEch1 (FGSG_13111)* | Reduced virulence |
| *ACXA17_08240* | *katA* | Unaffected pathogenicity |
| *ACXA17_08245* | *SoxR* | Reduced virulence |
| *ACXA17_08250* | *MGG_00383* | Reduced virulence |
| *ACXA17_08255* | *sodA1* | Reduced virulence |
| *ACXA17_08285* | *VK055_1961* | Unaffected pathogenicity |
| *ACXA17_08305* | *cps2J* | Lethal; Reduced virulence |
| *ACXA17_08325* | *cps2J* | Lethal; Reduced virulence |
| *ACXA17_08330* | *lacA* | Reduced virulence |
| *ACXA17_08335* | *cps2J* | Lethal; Reduced virulence |
| *ACXA17_08385* | *RIB1 (CAGL0F04279g)* | Reduced virulence |
| *ACXA17_08390* | *B9H01_RS05605* | Reduced virulence |
| *ACXA17_08430* | *CBU_0265* | Unaffected pathogenicity |
| *ACXA17_08465* | *eutD* | Reduced virulence |
| *ACXA17_08485* | *IMPDH* | Reduced virulence |
| *ACXA17_08490* | *LmABCB3* | Reduced virulence |
| *ACXA17_08500* | *Rho* | Reduced virulence |
| *ACXA17_08520* | *XOC_1946* | Reduced virulence |
| *ACXA17_08520* | *XOC_1946* | Reduced virulence |
| *ACXA17_08555* | *UGD1* | Loss of pathogenicity |
| *ACXA17_08560* | *ABC3* | Loss of pathogenicity |
| *ACXA17_08575* | *A0396* | Unaffected pathogenicity |
| *ACXA17_08590* | *CcoN2 (PA14_44340)* | Unaffected pathogenicity; Reduced virulence |
| *ACXA17_08600* | *copA* | Reduced virulence |
| *ACXA17_08670* | *PYR4 (XM_003719109)* | Reduced virulence |
| *ACXA17_08675* | *tufA* | Unaffected pathogenicity; Increased virulence |
| *ACXA17_08710* | *Rv0652 (rplL)* | Increased virulence |
| *ACXA17_08745* | *RpoB* | Reduced virulence |
| *ACXA17_08750* | *FgRbp1* | Reduced virulence |
| *ACXA17_08765* | *VraD* | Reduced virulence |
| *ACXA17_08780* | *paaB* | Reduced virulence |
| *ACXA17_08900* | *RpoN* | Reduced virulence |
| *ACXA17_08905* | *GzOB046* | Unaffected pathogenicity |
| *ACXA17_08965* | *PrsA2; prsA2* | Reduced virulence |
| *ACXA17_08985* | *himD* | Reduced virulence |
| *ACXA17_08990* | *RNase E* | Reduced virulence |
| *ACXA17_09035* | *PSPTO_2691* | Unaffected pathogenicity |
| *ACXA17_09115* | *PG0343* | Reduced virulence |
| *ACXA17_09165* | *pdh* | Reduced virulence |
| *ACXA17_09210* | *adeN* | Increased virulence |
| *ACXA17_09220* | *tbdR1* | Reduced virulence |
| *ACXA17_09280* | *Fur* | Reduced virulence |
| *ACXA17_09290* | *gabT (RSc0029)* | Reduced virulence |
| *ACXA17_09350* | *mecA* | Unaffected pathogenicity; Reduced virulence |
| *ACXA17_09375* | *iroC* | Reduced virulence |
| *ACXA17_09380* | *StpC* | Unaffected pathogenicity |
| *ACXA17_09405* | *Pbl1* | Unaffected pathogenicity |
| *ACXA17_09420* | *glmS* | Reduced virulence |
| *ACXA17_09425* | *GldK (AS87_08785)* | Reduced virulence |
| *ACXA17_09435* | *gldM (AS87_RS08465)* | Reduced virulence |
| *ACXA17_09440* | *gldN* | Reduced virulence |
| *ACXA17_09460* | *phoB* | Reduced virulence |
| *ACXA17_09465* | *walK* | Reduced virulence |
| *ACXA17_09485* | *Mfd* | Loss of pathogenicity |
| *ACXA17_09510* | *aroE* | Unaffected pathogenicity; Increased virulence |
| *ACXA17_09530* | *srrA* | Reduced virulence; Increased virulence |
| *ACXA17_09540* | *rpoE* | Reduced virulence |
| *ACXA17_09550* | *Bgp (BB_0588)* | Reduced virulence |
| *ACXA17_09565* | *ABC4* | Reduced virulence; Loss of pathogenicity |
| *ACXA17_09645* | *FGSG_07816* | Unaffected pathogenicity |
| *ACXA17_09685* | *ybdB* | Lethal |
| *ACXA17_09700* | *StpC* | Unaffected pathogenicity |
| *ACXA17_09735* | *ribC* | Unaffected pathogenicity |
| *ACXA17_09740* | *Flil* | Reduced virulence |
| *ACXA17_09750* | *XC_2203* | Reduced virulence |
| *ACXA17_09805* | *bipA* | Reduced virulence |
| *ACXA17_09810* | *PXO_RS21535 (StoS); stoS* | Unaffected pathogenicity; Reduced virulence |
| *ACXA17_09815* | *ntrC* | Reduced virulence |
| *ACXA17_09990* | *Fgbat1/2 (FGSG_09512)* | Unaffected pathogenicity |
| *ACXA17_10045* | *XC_0892* | Reduced virulence |
| *ACXA17_10050* | *DPPIV* | Reduced virulence |
| *ACXA17_10150* | *odhB* | Unaffected pathogenicity |
| *ACXA17_10155* | *pdh-e1alpha* | Unaffected pathogenicity |
| *ACXA17_10170* | *bcaP* | Unaffected pathogenicity; Reduced virulence |
| *ACXA17_10325* | *aroB* | Reduced virulence |
| *ACXA17_10350* | *MET13* | Loss of pathogenicity |
| *ACXA17_10360* | *AS87_RS09170 (bioF)* | Reduced virulence; Increased virulence |
| *ACXA17_10560* | *ClpP* | Reduced virulence |
| *ACXA17_10570* | *dbcA(bglu_1g06460)* | Reduced virulence |
| *ACXA17_10575* | *prfA (lmo2543)* | Reduced virulence |
| *ACXA17_10585* | *PXO_RS06935; pilR (PXO_04881)* | Unaffected pathogenicity; Reduced virulence |
| *ACXA17_10595* | *VdP4-4 (VDAG_00595)* | Reduced virulence |
| *ACXA17_10610* | *GzRad003* | Unaffected pathogenicity |
| *ACXA17_10690* | *XC_3129* | Reduced virulence |
| *ACXA17_10720* | *fabG1* | Lethal |
| *ACXA17_10725* | *STM14_1002* | Reduced virulence |
| *ACXA17_10730* | *XC_3615* | Reduced virulence |
| *ACXA17_10825* | *TRR1* | Lethal |

^a^Predicted genes associated with pathogen–host interactions in RA-FJ-C4.

^b^Homologous genes matched by BLAST alignment in the PHI database.

**Table S12 Predicted genes associated with pathogen–host interactions in RA-BJ-C1**

| **Gene ID^a^** | **Hit Gene Name^b^** | **Mutant Phenotype** |
| --- | --- | --- |
| *ACXA18_00010* | *BCAL2200* | Reduced virulence |
| *ACXA18_00050* | *ChpE (CMM_0039)* | Effector (plant avirulence determinant) |
| *ACXA18_00065* | *ksgA* | Reduced virulence |
| *ACXA18_00095* | *cob* | Unaffected pathogenicity |
| *ACXA18_00100* | *IDTR* | Reduced virulence |
| *ACXA18_00205* | *BbCypC* | Unaffected pathogenicity |
| *ACXA18_00225* | *Fox3p* | Unaffected pathogenicity |
| *ACXA18_00230* | *MoIVD (MGG_02540)* | Reduced virulence |
| *ACXA18_00315* | *oprF* | Reduced virulence |
| *ACXA18_00330* | *STM14_RS07785 (ttrS)* | Reduced virulence |
| *ACXA18_00355* | *PSPTO_2696* | Unaffected pathogenicity |
| *ACXA18_00360* | *murF* | Unaffected pathogenicity; Loss of pathogenicity |
| *ACXA18_00410* | *KdsD* | Reduced virulence |
| *ACXA18_00415* | *tatC* | Reduced virulence |
| *ACXA18_00435* | *tsaA* | Reduced virulence |
| *ACXA18_00460* | *mnmE* | Reduced virulence |
| *ACXA18_00505* | *ScpA* | Unaffected pathogenicity |
| *ACXA18_00535* | *Rv3213c* | Increased virulence |
| *ACXA18_00560* | *essC (lmo0061)* | Increased virulence |
| *ACXA18_00600* | *MepS* | Reduced virulence |
| *ACXA18_00630* | *Myco4* | Reduced virulence |
| *ACXA18_00635* | *gua1* | Loss of pathogenicity |
| *ACXA18_10080* | *Oxa-2* | Reduced virulence |
| *ACXA18_00710* | *idi* | Reduced virulence |
| *ACXA18_00720* | *pgm* | Reduced virulence; Increased virulence |
| *ACXA18_00800* | *cvfB* | Reduced virulence |
| *ACXA18_00865* | *URA3* | Reduced virulence |
| *ACXA18_00870* | *pqsD* | Reduced virulence |
| *ACXA18_00875* | *ADE5* | Reduced virulence |
| *ACXA18_00880* | *purN* | Unaffected pathogenicity; Reduced virulence |
| *ACXA18_00890* | *asnB* | Unaffected pathogenicity |
| *ACXA18_00900* | *StpC* | Unaffected pathogenicity |
| *ACXA18_00920* | *FGSG_13826* | Unaffected pathogenicity |
| *ACXA18_00995* | *bglB* | Reduced virulence; Increased virulence |
| *ACXA18_01030* | *rny; cvfA* | Reduced virulence |
| *ACXA18_01055* | *GyrA* | Chemistry target: sensitivity to chemical |
| *ACXA18_01060* | *rsh* | Unaffected pathogenicity; Reduced virulence |
| *ACXA18_01065* | *nhaA* | Reduced virulence |
| *ACXA18_01070* | *gGT* | Reduced virulence |
| *ACXA18_01090* | *TRPS* | Lethal |
| *ACXA18_01100* | *trpB* | Reduced virulence |
| *ACXA18_01110* | *trpC (PXO_04004)* | Reduced virulence |
| *ACXA18_01130* | *trpA* | Reduced virulence |
| *ACXA18_01155* | *clbD* | Unaffected pathogenicity |
| *ACXA18_01175* | *purF (Dda3937_02099)* | Reduced virulence |
| *ACXA18_01180* | *GzOB034* | Unaffected pathogenicity |
| *ACXA18_01190* | *PurA* | Reduced virulence |
| *ACXA18_01210* | *bipA* | Reduced virulence |
| *ACXA18_01295* | *yaaA* | Reduced virulence |
| *ACXA18_01335* | *pnp* | Reduced virulence |
| *ACXA18_01370* | *mexB* | Unaffected pathogenicity |
| *ACXA18_01430* | *cps2J* | Lethal; Reduced virulence |
| *ACXA18_01440* | *acs* | Unaffected pathogenicity; Reduced virulence |
| *ACXA18_01465* | *Lmo0866; CshA* | Unaffected pathogenicity; Increased virulence |
| *ACXA18_01490* | *GyrA* | Chemistry target: resistance to chemical; Chemistry target: sensitivity to chemical |
| *ACXA18_01505* | *SSBX* | Reduced virulence |
| *ACXA18_01530* | *MoIVD (MGG_02540)* | Reduced virulence |
| *ACXA18_01550* | *ppc* | Unaffected pathogenicity |
| *ACXA18_01575* | *pimt* | Reduced virulence |
| *ACXA18_01650* | *SpxA2* | Unaffected pathogenicity; Reduced virulence; Increased virulence |
| *ACXA18_01710* | *tktA (FTN_1333)* | Unaffected pathogenicity |
| *ACXA18_01740* | *yedX* | Unaffected pathogenicity |
| *ACXA18_01745* | *pdhB* | Reduced virulence |
| *ACXA18_01765* | *GroEL* | Increased virulence |
| *ACXA18_01815* | *galE* | Unaffected pathogenicity; Reduced virulence |
| *ACXA18_01820* | *arnB* | Unaffected pathogenicity |
| *ACXA18_01860* | *ADE13* | Reduced virulence |
| *ACXA18_01880* | *fabG1* | Lethal |
| *ACXA18_01890* | *MoSCAD2 (MGG_08690)* | Loss of pathogenicity |
| *ACXA18_01895* | *Lmo0866; CshA* | Unaffected pathogenicity; Increased virulence |
| *ACXA18_01905* | *MoSCAD2 (MGG_08690)* | Loss of pathogenicity |
| *ACXA18_01910* | *fadD2* | Reduced virulence |
| *ACXA18_01980* | *odhB* | Unaffected pathogenicity |
| *ACXA18_01985* | *VdOGDH (VDAG_10018)* | Reduced virulence |
| *ACXA18_02005* | *atpG* | Reduced virulence |
| *ACXA18_02010* | *ATP1* | Loss of pathogenicity |
| *ACXA18_02095* | *GUS1* | Lethal |
| *ACXA18_02115* | *nit2* | Unaffected pathogenicity |
| *ACXA18_02130* | *hmuV* | Unaffected pathogenicity |
| *ACXA18_02155* | *carA (Dda3937_01390)* | Reduced virulence |
| *ACXA18_02175* | *cspC* | Unaffected pathogenicity; Reduced virulence; Loss of pathogenicity |
| *ACXA18_02185* | *RhlE2 (PA0428); RhlE1 (PA3950)* | Reduced virulence |
| *ACXA18_02235* | *CLC-A* | Reduced virulence |
| *ACXA18_02255* | *Als1; ALS1* | Reduced virulence; Loss of pathogenicity |
| *ACXA18_02270* | *cys4* | Reduced virulence |
| *ACXA18_02290* | *LpdA* | Effector (plant avirulence determinant) |
| *ACXA18_02295* | *FTT1564* | Unaffected pathogenicity |
| *ACXA18_02300* | *FTT1564* | Unaffected pathogenicity |
| *ACXA18_02315* | *thioredoxin 1* | Reduced virulence |
| *ACXA18_02415* | *Mfd* | Loss of pathogenicity |
| *ACXA18_02420* | *potA* | Reduced virulence |
| *ACXA18_02470* | *PitA* | Unaffected pathogenicity; Reduced virulence |
| *ACXA18_02485* | *MSY1* | Reduced virulence |
| *ACXA18_02535* | *radC* | Reduced virulence |
| *ACXA18_02540* | *VraD* | Reduced virulence |
| *ACXA18_02565* | *folP* | Unaffected pathogenicity |
| *ACXA18_02585* | *XC_2466* | Reduced virulence |
| *ACXA18_02590* | *hupA* | Reduced virulence |
| *ACXA18_02620* | *aroB* | Reduced virulence |
| *ACXA18_02650* | *lipA (SAUSA300_0829)* | Reduced virulence |
| *ACXA18_02690* | *Zmp1* | Increased virulence |
| *ACXA18_02735* | *ireA* | Unaffected pathogenicity |
| *ACXA18_02750* | *ppk* | Reduced virulence |
| *ACXA18_02840* | *OmpA1* | Reduced virulence |
| *ACXA18_02845* | *OmpA1* | Reduced virulence |
| *ACXA18_02870* | *greA* | Loss of pathogenicity |
| *ACXA18_02880* | *ClpX* | Reduced virulence |
| *ACXA18_03250* | *SPD_1366 (CvfD)* | Reduced virulence |
| *ACXA18_03255* | *pdhB* | Reduced virulence |
| *ACXA18_03275* | *recA* | Reduced virulence |
| *ACXA18_03285* | *GAPDH* | Reduced virulence |
| *ACXA18_03290* | *pfkA* | Reduced virulence |
| *ACXA18_03300* | *bfpR (THC0290_1679)* | Unaffected pathogenicity; Reduced virulence |
| *ACXA18_03320* | *PA4485* | Unaffected pathogenicity |
| *ACXA18_03410* | *lpxD1* | Loss of pathogenicity |
| *ACXA18_03415* | *EF-P* | Increased virulence |
| *ACXA18_03420* | *lpxA* | Reduced virulence |
| *ACXA18_03425* | *lpxA* | Reduced virulence |
| *ACXA18_03430* | *lpxD* | Reduced virulence |
| *ACXA18_03485* | *pnp* | Reduced virulence |
| *ACXA18_03495* | *ldh-1* | Unaffected pathogenicity; Reduced virulence |
| *ACXA18_03505* | *FMT* | Reduced virulence |
| *ACXA18_03510* | *MurI* | Reduced virulence |
| *ACXA18_03540* | *gumD* | Loss of pathogenicity |
| *ACXA18_03545* | *mlaE* | Reduced virulence |
| *ACXA18_03550* | *proV* | Reduced virulence |
| *ACXA18_03570* | *ydiD* | Reduced virulence |
| *ACXA18_03585* | *ADE2* | Reduced virulence |
| *ACXA18_03590* | *ADE2* | Reduced virulence; Loss of pathogenicity |
| *ACXA18_03655* | *XC_0450* | Reduced virulence |
| *ACXA18_03700* | *Rv0462 (lpdC)* | Increased virulence |
| *ACXA18_03710* | *StpC* | Unaffected pathogenicity |
| *ACXA18_03715* | *dnaJ* | Reduced virulence |
| *ACXA18_03725* | *mntH2* | Unaffected pathogenicity; Reduced virulence |
| *ACXA18_03730* | *B739_1208* | Reduced virulence |
| *ACXA18_03755* | *rpe (FTN_1221)* | Reduced virulence |
| *ACXA18_03760* | *rpoS* | Unaffected pathogenicity |
| *ACXA18_03765* | *MoHYR1; MoHyr1* | Reduced virulence |
| *ACXA18_03790* | *PGM* | Reduced virulence |
| *ACXA18_03800* | *arnC* | Unaffected pathogenicity |
| *ACXA18_03855* | *ERG10* | Lethal |
| *ACXA18_03865* | *pepP (CJJ81176_0681)* | Reduced virulence |
| *ACXA18_03925* | *lsrA* | Reduced virulence |
| *ACXA18_03930* | *gcvP* | Reduced virulence |
| *ACXA18_03950* | *dnaJ* | Reduced virulence |
| *ACXA18_03975* | *MnhA1* | Reduced virulence |
| *ACXA18_03985* | *nuoJ (PA2645)* | Unaffected pathogenicity; Reduced virulence |
| *ACXA18_04000* | *nuoG* | Unaffected pathogenicity |
| *ACXA18_04030* | *STM14_RS15275* | Unaffected pathogenicity |
| *ACXA18_04095* | *rmlA* | Reduced virulence; Loss of pathogenicity |
| *ACXA18_04100* | *USX1* | Loss of pathogenicity |
| *ACXA18_04110* | *gumD* | Loss of pathogenicity |
| *ACXA18_04115* | *pglE* | Reduced virulence |
| *ACXA18_04120* | *gigX6* | Increased virulence |
| *ACXA18_04125* | *gumD* | Loss of pathogenicity |
| *ACXA18_04150* | *cps2J* | Lethal; Reduced virulence |
| *ACXA18_04160* | *cps2J* | Lethal; Reduced virulence |
| *ACXA18_04170* | *epsC* | Reduced virulence |
| *ACXA18_04175* | *algD* | Reduced virulence |
| *ACXA18_04185* | *AS87_04050* | Reduced virulence |
| *ACXA18_04195* | *wza* | Reduced virulence |
| *ACXA18_04200* | *wbtA* | Reduced virulence |
| *ACXA18_04210* | *glyA* | Reduced virulence |
| *ACXA18_04215* | *pgi* | Reduced virulence |
| *ACXA18_04245* | *aroA* | Unaffected pathogenicity; Reduced virulence |
| *ACXA18_04300* | *pyrG* | Unaffected pathogenicity |
| *ACXA18_04305* | *RecA* | Reduced virulence |
| *ACXA18_04375* | *Nmo2* | Reduced virulence |
| *ACXA18_04420* | *nfu* | Reduced virulence |
| *ACXA18_04690* | *clpB* | Unaffected pathogenicity |
| *ACXA18_04720* | *MoCA1 (MGG_04611)* | Reduced virulence |
| *ACXA18_04725* | *lacA* | Reduced virulence |
| *ACXA18_04775* | *MFP1* | Reduced virulence |
| *ACXA18_04810* | *AmiC1 (XC_1816)* | Reduced virulence |
| *ACXA18_04830* | *Eno* | Unaffected pathogenicity |
| *ACXA18_04885* | *secY* | Increased virulence |
| *ACXA18_04935* | *rplN* | Unaffected pathogenicity; Increased virulence |
| *ACXA18_04970* | *GzOB009* | Lethal |
| *ACXA18_05020* | *yeaZ* | Reduced virulence |
| *ACXA18_05030* | *accD* | Reduced virulence |
| *ACXA18_05105* | *LmABCB3* | Reduced virulence |
| *ACXA18_05160* | *cps2J* | Lethal; Reduced virulence |
| *ACXA18_05165* | *degQ (Dda3937_00697)* | Reduced virulence |
| *ACXA18_05170* | *yhbU* | Unaffected pathogenicity |
| *ACXA18_05305* | *PykM (BAB1_1761); pyk* | Reduced virulence |
| *ACXA18_05315* | *rnc* | Unaffected pathogenicity; Reduced virulence |
| *ACXA18_05320* | *KSA1; PKS9* | Unaffected pathogenicity |
| *ACXA18_05325* | *Acp1 (PA1869)* | Increased virulence |
| *ACXA18_05390* | *HP0102* | Reduced virulence |
| *ACXA18_05410* | *argD* | Unaffected pathogenicity; Reduced virulence; Loss of pathogenicity |
| *ACXA18_05415* | *DUR1,2* | Reduced virulence |
| *ACXA18_05445* | *sreS* | Unaffected pathogenicity |
| *ACXA18_05455* | *VraD* | Reduced virulence |
| *ACXA18_05500* | *yegN* | Reduced virulence |
| *ACXA18_05545* | *prfA (lmo2543)* | Reduced virulence |
| *ACXA18_05560* | *MopB* | Reduced virulence |
| *ACXA18_05565* | *smpB* | Reduced virulence |
| *ACXA18_05570* | *cpxA* | Reduced virulence |
| *ACXA18_05605* | *VK055_1952* | Unaffected pathogenicity |
| *ACXA18_05630* | *A1479* | Reduced virulence |
| *ACXA18_05640* | *murD* | Reduced virulence; Loss of pathogenicity |
| *ACXA18_05655* | *pbpC* | Unaffected pathogenicity; Increased virulence |
| *ACXA18_05665* | *rsmH* | Reduced virulence |
| *ACXA18_05690* | *msrA2* | Unaffected pathogenicity; Reduced virulence |
| *ACXA18_05695* | *Lon* | Loss of pathogenicity |
| *ACXA18_05725* | *mazF* | Unaffected pathogenicity; Increased virulence |
| *ACXA18_04200* | *GacA* | Lethal |
| *ACXA18_05770* | *B739_0832 (OmpH)* | Reduced virulence |
| *ACXA18_05775* | *omp17* | Reduced virulence |
| *ACXA18_05805* | *CDS1* | Lethal |
| *ACXA18_05875* | *mlh1* | Unaffected pathogenicity |
| *ACXA18_05955* | *PGK2* | Unaffected pathogenicity |
| *ACXA18_05980* | *gidA* | Reduced virulence |
| *ACXA18_06025* | *PCK1* | Reduced virulence |
| *ACXA18_06035* | *mexE* | Increased virulence |
| *ACXA18_06040* | *mexF* | Increased virulence |
| *ACXA18_06045* | *adeK* | Unaffected pathogenicity |
| *ACXA18_03790* | *RfbB* | Reduced virulence |
| *ACXA18_06115* | *PpdaAc* | Reduced virulence |
| *ACXA18_06140* | *cps2J* | Lethal; Reduced virulence |
| *ACXA18_06145* | *bipA* | Reduced virulence |
| *ACXA18_06160* | *sufB; STM14_RS07705 (sufB)* | Unaffected pathogenicity; Reduced virulence |
| *ACXA18_06190* | *STM14_RS07710 (sufC)* | Reduced virulence |
| *ACXA18_06195* | *STM14_RS07715 (sufD)* | Reduced virulence |
| *ACXA18_06220* | *srrB* | Reduced virulence; Increased virulence |
| *ACXA18_06225* | *cpxR (BFW97_22310)* | Reduced virulence |
| *ACXA18_06235* | *murA* | Unaffected pathogenicity; Reduced virulence |
| *ACXA18_06240* | *cptRNA-ipt* | Reduced virulence; Loss of pathogenicity |
| *ACXA18_06275* | *gloB* | Unaffected pathogenicity; Reduced virulence |
| *ACXA18_06295* | *murF* | Unaffected pathogenicity; Loss of pathogenicity |
| *ACXA18_00015* | *rsmI* | Reduced virulence |
| *ACXA18_06315* | *fabG1* | Lethal |
| *ACXA18_06320* | *pbpX* | Reduced virulence |
| *ACXA18_06335* | *ssdA (AFUB_010850)* | Reduced virulence; Increased virulence |
| *ACXA18_06355* | *clpB* | Reduced virulence |
| *ACXA18_06365* | *STM14_RS07725 (ynhA/sufE)* | Reduced virulence |
| *ACXA18_06385* | *Prc* | Reduced virulence |
| *ACXA18_06440* | *PG_RS04465 (PG1010)* | Reduced virulence |
| *ACXA18_06500* | *MoMca1 (MGG_04626)* | Reduced virulence |
| *ACXA18_06530* | *Oligopeptidase B* | Reduced virulence |
| *ACXA18_06570* | *cps2J* | Lethal; Reduced virulence |
| *ACXA18_06590* | *BCAM1331* | Reduced virulence |
| *ACXA18_06595* | *msh2* | Unaffected pathogenicity |
| *ACXA18_06630* | *ftsH* | Unaffected pathogenicity |
| *ACXA18_06960* | *uvrB* | Unaffected pathogenicity |
| *ACXA18_06985* | *FTT0673p/prsAp* | Unaffected pathogenicity |
| *ACXA18_07005* | *yfiB* | Unaffected pathogenicity |
| *ACXA18_07055* | *lytS* | Unaffected pathogenicity; Reduced virulence |
| *ACXA18_07100* | *sip* | Reduced virulence |
| *ACXA18_07160* | *feoB* | Loss of pathogenicity |
| *ACXA18_07205* | *RSp0930* | Effector (plant avirulence determinant) |
| *ACXA18_07255* | *fabD* | Reduced virulence |
| *ACXA18_07355* | *fleQ* | Unaffected pathogenicity; Increased virulence; Reduced virulence; Loss of pathogenicity |
| *ACXA18_07420* | *AS87_04050* | Reduced virulence |
| *ACXA18_07430* | *secA* | Reduced virulence |
| *ACXA18_07440* | *hmuR* | Unaffected pathogenicity |
| *ACXA18_07470* | *mnmE* | Reduced virulence |
| *ACXA18_07525* | *ireA* | Unaffected pathogenicity |
| *ACXA18_07580* | *fumA* | Reduced virulence |
| *ACXA18_02320* | *STM14_RS07720 (sufS)* | Reduced virulence |
| *ACXA18_07620* | *MakatG1* | Unaffected pathogenicity; Reduced virulence |
| *ACXA18_07650* | *GzOB039* | Unaffected pathogenicity |
| *ACXA18_07710* | *hemH* | Unaffected pathogenicity |
| *ACXA18_07795* | *FgEch1 (FGSG_13111)* | Reduced virulence |
| *ACXA18_07805* | *katA* | Unaffected pathogenicity |
| *ACXA18_07810* | *SoxR* | Reduced virulence |
| *ACXA18_07815* | *MGG_00383* | Reduced virulence |
| *ACXA18_07820* | *sodA1* | Reduced virulence |
| *ACXA18_07850* | *VK055_1961* | Unaffected pathogenicity |
| *ACXA18_07870* | *cps2J* | Lethal; Reduced virulence |
| *ACXA18_07900* | *cps2J* | Lethal; Reduced virulence |
| *ACXA18_07905* | *lacA* | Reduced virulence |
| *ACXA18_07910* | *cps2J* | Lethal; Reduced virulence |
| *ACXA18_07960* | *RIB1 (CAGL0F04279g)* | Reduced virulence |
| *ACXA18_07965* | *B9H01_RS05605* | Reduced virulence |
| *ACXA18_07995* | *CBU_0265* | Unaffected pathogenicity |
| *ACXA18_08020* | *eutD* | Reduced virulence |
| *ACXA18_08040* | *IMPDH* | Reduced virulence |
| *ACXA18_08045* | *LmABCB3* | Reduced virulence |
| *ACXA18_08055* | *Rho* | Reduced virulence |
| *ACXA18_08075* | *XOC_1946* | Reduced virulence |
| *ACXA18_08110* | *UGD1* | Loss of pathogenicity |
| *ACXA18_08115* | *ABC3* | Loss of pathogenicity |
| *ACXA18_08130* | *A0396* | Unaffected pathogenicity |
| *ACXA18_08145* | *CcoN2 (PA14_44340)* | Unaffected pathogenicity; Reduced virulence |
| *ACXA18_08155* | *copA* | Reduced virulence |
| *ACXA18_08225* | *PYR4 (XM_003719109)* | Reduced virulence |
| *ACXA18_08230* | *tufA* | Unaffected pathogenicity; Increased virulence |
| *ACXA18_08265* | *Rv0652 (rplL)* | Increased virulence |
| *ACXA18_08290* | *RpoB* | Reduced virulence |
| *ACXA18_08295* | *FgRbp1* | Reduced virulence |
| *ACXA18_08310* | *VraD* | Reduced virulence |
| *ACXA18_08325* | *paaB* | Reduced virulence |
| *ACXA18_08450* | *RpoN* | Reduced virulence |
| *ACXA18_08455* | *GzOB046* | Unaffected pathogenicity |
| *ACXA18_08515* | *PrsA2; prsA2* | Reduced virulence |
| *ACXA18_08535* | *himD* | Reduced virulence |
| *ACXA18_08540* | *RNase E* | Reduced virulence |
| *ACXA18_08585* | *PSPTO_2691* | Unaffected pathogenicity |
| *ACXA18_08665* | *PG0343* | Reduced virulence |
| *ACXA18_08715* | *pdh* | Reduced virulence |
| *ACXA18_08745* | *ypo2062* | Reduced virulence |
| *ACXA18_08805* | *adeN* | Increased virulence |
| *ACXA18_08815* | *tbdR1* | Reduced virulence |
| *ACXA18_08865* | *Fur* | Reduced virulence |
| *ACXA18_05410* | *gabT (RSc0029)* | Reduced virulence |
| *ACXA18_08925* | *mecA* | Unaffected pathogenicity; Reduced virulence |
| *ACXA18_08950* | *iroC* | Reduced virulence |
| *ACXA18_08955* | *StpC* | Unaffected pathogenicity |
| *ACXA18_08980* | *Pbl1* | Unaffected pathogenicity |
| *ACXA18_08990* | *glmS* | Reduced virulence |
| *ACXA18_09000* | *GldK (AS87_08785)* | Reduced virulence |
| *ACXA18_09010* | *gldM (AS87_RS08465)* | Reduced virulence |
| *ACXA18_09015* | *gldN* | Reduced virulence |
| *ACXA18_09035* | *phoB* | Reduced virulence |
| *ACXA18_09040* | *walK* | Reduced virulence |
| *ACXA18_09060* | *Mfd* | Loss of pathogenicity |
| *ACXA18_09085* | *aroE* | Unaffected pathogenicity; Increased virulence |
| *ACXA18_09105* | *srrA* | Reduced virulence; Increased virulence |
| *ACXA18_09115* | *rpoE* | Reduced virulence |
| *ACXA18_09125* | *Bgp (BB_0588)* | Reduced virulence |
| *ACXA18_09140* | *ABC4* | Reduced virulence; Loss of pathogenicity |
| *ACXA18_09220* | *FGSG_07816* | Unaffected pathogenicity |
| *ACXA18_09260* | *ybdB* | Lethal |
| *ACXA18_09275* | *StpC* | Unaffected pathogenicity |
| *ACXA18_09325* | *ribC* | Unaffected pathogenicity |
| *ACXA18_09330* | *Flil* | Reduced virulence |
| *ACXA18_09340* | *XC_2203* | Reduced virulence |
| *ACXA18_09345* | *Fgbat1/2 (FGSG_09512)* | Unaffected pathogenicity |
| *ACXA18_09400* | *XC_0892* | Reduced virulence |
| *ACXA18_09405* | *DPPIV* | Reduced virulence |
| *ACXA18_09790* | *odhB* | Unaffected pathogenicity |
| *ACXA18_09795* | *pdh-e1alpha* | Unaffected pathogenicity |
| *ACXA18_09810* | *bcaP* | Unaffected pathogenicity; Reduced virulence |
| *ACXA18_09965* | *aroB* | Reduced virulence |
| *ACXA18_09990* | *MET13* | Loss of pathogenicity |
| *ACXA18_10000* | *AS87_RS09170 (bioF)* | Reduced virulence; Increased virulence |
| *ACXA18_10050* | *Rv1988* | Reduced virulence |
| *ACXA18_10125* | *dfr1* | Reduced virulence |
| *ACXA18_10130* | *GzZC087* | Reduced virulence |
| *ACXA18_10530* | *ClpP* | Reduced virulence |
| *ACXA18_10540* | *dbcA(bglu_1g06460)* | Reduced virulence |
| *ACXA18_10545* | *prfA (lmo2543)* | Reduced virulence |
| *ACXA18_10555* | *PXO_RS06935; pilR (PXO_04881)* | Unaffected pathogenicity; Reduced virulence |
| *ACXA18_10565* | *VdP4-4 (VDAG_00595)* | Reduced virulence |
| *ACXA18_10580* | *GzRad003* | Unaffected pathogenicity |
| *ACXA18_10660* | *XC_3129* | Reduced virulence |
| *ACXA18_10690* | *fabG1* | Lethal |
| *ACXA18_10695* | *STM14_1002* | Reduced virulence |
| *ACXA18_10700* | *XC_3615* | Reduced virulence |
| *ACXA18_10780* | *TRR1* | Lethal |

^a^Predicted genes associated with pathogen–host interactions in RA-BJ-C1.

^b^Homologous genes matched by BLAST alignment in the PHI database.

**Table S13** **RA-FJ-C1 strain-specific genes**

| **Gene ID^a^** | **Gene Descriptions** |
| --- | --- |
| *ACXA16_00075* | Hypothetical protein |
| *ACXA16_00080* | SIR2 family protein |
| *ACXA16_00095* | IPT/TIG domain-containing protein |
| *ACXA16_00145* | Hypothetical protein |
| *ACXA16_00150* | Hypothetical protein |
| *ACXA16_00155* | Hypothetical protein |
| *ACXA16_00200* | Probable site-specific recombinase |
| *ACXA16_00175* | Hypothetical protein |
| *ACXA16_00180* | YncE family protein |
| *ACXA16_00245* | Hypothetical protein |
| *ACXA16_00480* | Subtype B tannase |
| *ACXA16_00515* | Immunity protein Imm33 domain-containing protein |
| *ACXA16_00520* | CPBP family glutamic-type intramembrane protease |
| *ACXA16_00525* | Type II toxin-antitoxin system prevent-host-death family antitoxin |
| *ACXA16_00530* | Txe/YoeB family addiction module toxin |
| *ACXA16_00620* | Hypothetical protein |
| *ACXA16_00705* | Hypothetical protein |
| *ACXA16_00710* | Hypothetical protein |
| *ACXA16_00760* | Hypothetical protein |
| *ACXA16_00795* | Hypothetical protein |
| *ACXA16_00805* | GLPGLI family protein |
| *ACXA16_00850* | Hypothetical protein |
| *ACXA16_00960* | Hypothetical protein |
| *ACXA16_00980* | ABC transporter permease |
| *ACXA16_01130* | Hypothetical protein |
| *ACXA16_01145* | Tryptophan synthase subunit alpha |
| *ACXA16_01235* | 5-methylcytosine restriction system specificity protein McrC |
| *ACXA16_01240* | AAA family ATPase |
| *ACXA16_01330* | Hypothetical protein |
| *ACXA16_01335* | Porin family protein |
| *ACXA16_01340* | Hypothetical protein |
| *ACXA16_01345* | Hypothetical protein |
| *ACXA16_01395* | Endonuclease NucS domain-containing protein |
| *ACXA16_01420* | Hypothetical protein |
| *ACXA16_01425* | VapE domain-containing protein |
| *ACXA16_01430* | Hypothetical protein |
| *ACXA16_01435* | Hypothetical protein |
| *ACXA16_01440* | Tyrosine-type recombinase/integrase |
| *ACXA16_01445* | Metal-dependent hydrolase |
| *ACXA16_01455* | Hypothetical protein |
| *ACXA16_01460* | Type II toxin-antitoxin system RelE/ParE family toxin |
| *ACXA16_01465* | Hypothetical protein |
| *ACXA16_01535* | Beta-lactamase |
| *ACXA16_01545* | DUF6058 family natural product biosynthesis protein |
| *ACXA16_01550* | Hypothetical protein |
| *ACXA16_01685* | Hypothetical protein |
| *ACXA16_01695* | FEKKY domain-containing protein |
| *ACXA16_01700* | Hypothetical protein |
| *ACXA16_01705* | FAD-dependent monooxygenase |
| *ACXA16_01740* | Hypothetical protein |
| *ACXA16_01745* | Hypothetical protein |
| *ACXA16_01775* | Hypothetical protein |
| *ACXA16_01915* | Hypothetical protein |
| *ACXA16_02010* | Helix-turn-helix transcriptional regulator |
| *ACXA16_02015* | Hypothetical protein |
| *ACXA16_02020* | Hypothetical protein |
| *ACXA16_02025* | Primase-helicase family protein |
| *ACXA16_02030* | Hypothetical protein |
| *ACXA16_02035* | Hypothetical protein |
| *ACXA16_02040* | Hypothetical protein |
| *ACXA16_02045* | Hypothetical protein |
| *ACXA16_02050* | DUF7352 domain-containing protein |
| *ACXA16_02055* | Hypothetical protein |
| *ACXA16_02060* | Hypothetical protein |
| *ACXA16_02070* | Hypothetical protein |
| *ACXA16_02075* | S49 family peptidase |
| *ACXA16_02080* | Hypothetical protein |
| *ACXA16_02085* | Hypothetical protein |
| *ACXA16_02090* | Hypothetical protein |
| *ACXA16_02095* | Hypothetical protein |
| *ACXA16_02100* | Hypothetical protein |
| *ACXA16_02105* | Hypothetical protein |
| *ACXA16_02110* | DNA modification methylase |
| *ACXA16_02115* | Hypothetical protein |
| *ACXA16_02120* | Hypothetical protein |
| *ACXA16_02130* | Hypothetical protein |
| *ACXA16_02135* | DUF6712 family protein |
| *ACXA16_02140* | Hypothetical protein |
| *ACXA16_02150* | Hypothetical protein |
| *ACXA16_02155* | DUF1353 domain-containing protein |
| *ACXA16_02160* | Phage holin family protein |
| *ACXA16_02165* | Hypothetical protein |
| *ACXA16_02175* | Hypothetical protein |
| *ACXA16_02180* | Hypothetical protein |
| *ACXA16_02185* | Type II toxin-antitoxin system HicA family toxin |
| *ACXA16_02190* | Type II toxin-antitoxin system HicB family antitoxin |
| *ACXA16_02195* | Exonuclease domain-containing protein |
| *ACXA16_02200* | Hypothetical protein |
| *ACXA16_02205* | Hypothetical protein |
| *ACXA16_02210* | Hypothetical protein |
| *ACXA16_02215* | Hypothetical protein |
| *ACXA16_02220* | Hypothetical protein |
| *ACXA16_02225* | Hypothetical protein |
| *ACXA16_02230* | Hypothetical protein |
| *ACXA16_02235* | Hypothetical protein |
| *ACXA16_02240* | Phage tail tape measure protein |
| *ACXA16_02245* | Hypothetical protein |
| *ACXA16_02250* | Hypothetical protein |
| *ACXA16_02255* | Hypothetical protein |
| *ACXA16_02260* | Site-specific integrase |
| *ACXA16_02280* | Hypothetical protein |
| *ACXA16_02300* | GLPGLI family protein |
| *ACXA16_02530* | Sensor histidine kinase |
| *ACXA16_02535* | Hypothetical protein |
| *ACXA16_02540* | Hypothetical protein |
| *ACXA16_02545* | DNA methyltransferase |
| *ACXA16_02585* | Hypothetical protein |
| *ACXA16_02600* | DUF6261 family protein |
| *ACXA16_02605* | Hypothetical protein |
| *ACXA16_02630* | Hypothetical protein |
| *ACXA16_02635* | Hypothetical protein |
| *ACXA16_02640* | Hypothetical protein |
| *ACXA16_02645* | Hypothetical protein |
| *ACXA16_02650* | Hypothetical protein |
| *ACXA16_02655* | DUF2750 domain-containing protein |
| *ACXA16_02660* | Polymorphic toxin type 8 domain-containing protein |
| *ACXA16_02670* | Hypothetical protein |
| *ACXA16_02675* | RHS repeat-associated core domain-containing protein |
| *ACXA16_02685* | IS982-like element ISRa1 family transposase |
| *ACXA16_02690* | IS982-like element ISRa1 family transposase |
| *ACXA16_02695* | Transposase is4 family protein |
| *ACXA16_02700* | Hypothetical protein |
| *ACXA16_02705* | T9SS type A sorting domain-containing protein |
| *ACXA16_02740* | GIY-YIG nuclease family protein |
| *ACXA16_02745* | Hypothetical protein |
| *ACXA16_02775* | ImmA/IrrE family metallo-endopeptidase |
| *ACXA16_02780* | DUF4411 family protein |
| *ACXA16_02885* | DUF5977 domain-containing protein |
| *ACXA16_02890* | Hypothetical protein |
| *ACXA16_02895* | Hypothetical protein |
| *ACXA16_02920* | DEAD/DEAH box helicase |
| *ACXA16_02925* | Hypothetical protein |
| *ACXA16_02930* | Hypothetical protein |
| *ACXA16_03255* | Hypothetical protein |
| *ACXA16_03260* | Hypothetical protein |
| *ACXA16_03305* | Hypothetical protein |
| *ACXA16_03530* | Hypothetical protein |
| *ACXA16_03535* | Hypothetical protein |
| *ACXA16_03550* | T9SS type A sorting domain-containing protein |
| *ACXA16_03635* | Site-specific integrase |
| *ACXA16_03640* | Hypothetical protein |
| *ACXA16_03645* | Hypothetical protein |
| *ACXA16_03655* | Hypothetical protein |
| *ACXA16_03660* | Phage tail tape measure protein |
| *ACXA16_03665* | Hypothetical protein |
| *ACXA16_03670* | Hypothetical protein |
| *ACXA16_03675* | Type I addiction module toxin, SymE family |
| *ACXA16_03680* | DUF6943 family protein |
| *ACXA16_03695* | Hypothetical protein |
| *ACXA16_03700* | Hypothetical protein |
| *ACXA16_03705* | Hypothetical protein |
| *ACXA16_03710* | DUF6712 family protein |
| *ACXA16_03715* | Hypothetical protein |
| *ACXA16_03720* | Hypothetical protein |
| *ACXA16_03725* | Hypothetical protein |
| *ACXA16_03730* | Hypothetical protein |
| *ACXA16_03735* | Hypothetical protein |
| *ACXA16_03740* | Hypothetical protein |
| *ACXA16_03745* | BRCT domain-containing protein |
| *ACXA16_03750* | DUF5053 domain-containing protein |
| *ACXA16_03755* | Hypothetical protein |
| *ACXA16_03760* | Hypothetical protein |
| *ACXA16_03765* | Hypothetical protein |
| *ACXA16_03770* | Hypothetical protein |
| *ACXA16_03780* | Hypothetical protein |
| *ACXA16_03785* | Hypothetical protein |
| *ACXA16_03790* | Hypothetical protein |
| *ACXA16_03795* | Hypothetical protein |
| *ACXA16_03800* | S49 family peptidase |
| *ACXA16_03805* | M23 family metallopeptidase |
| *ACXA16_03810* | Hypothetical protein |
| *ACXA16_03815* | Phage holin family protein |
| *ACXA16_03820* | DUF1353 domain-containing protein |
| *ACXA16_03825* | Hypothetical protein |
| *ACXA16_03830* | Hypothetical protein |
| *ACXA16_03835* | Hypothetical protein |
| *ACXA16_03840* | Hypothetical protein |
| *ACXA16_03845* | Hypothetical protein |
| *ACXA16_03850* | Hypothetical protein |
| *ACXA16_03855* | Hypothetical protein |
| *ACXA16_03860* | Hypothetical protein |
| *ACXA16_03865* | Hypothetical protein |
| *ACXA16_03870* | Hypothetical protein |
| *ACXA16_03875* | Hypothetical protein |
| *ACXA16_03880* | DNA primase |
| *ACXA16_03885* | Hypothetical protein |
| *ACXA16_03890* | Hypothetical protein |
| *ACXA16_03895* | Hypothetical protein |
| *ACXA16_03900* | Helix-turn-helix transcriptional regulator |
| *ACXA16_03905* | Hypothetical protein |
| *ACXA16_03910* | Hypothetical protein |
| *ACXA16_03925* | Hypothetical protein |
| *ACXA16_04050* | Hypothetical protein |
| *ACXA16_04065* | Hypothetical protein |
| *ACXA16_04085* | Lipocalin family protein |
| *ACXA16_04170* | TlpA disulfide reductase family protein |
| *ACXA16_04265* | Hypothetical protein |
| *ACXA16_04530* | SDR family oxidoreductase |
| *ACXA16_04535* | Acyl carrier protein |
| *ACXA16_04540* | HAD-IIIC family phosphatase |
| *ACXA16_04545* | VOC family protein |
| *ACXA16_04550* | Ketoacyl-ACP synthase III |
| *ACXA16_04555* | Acyl carrier protein |
| *ACXA16_04560* | Acetyltransferase |
| *ACXA16_04575* | Asparagine synthase C-terminal domain-containing protein |
| *ACXA16_04580* | Hypothetical protein |
| *ACXA16_04585* | Hypothetical protein |
| *ACXA16_04590* | Glycosyltransferase family 4 protein |
| *ACXA16_04595* | Glycosyltransferase family 2 protein |
| *ACXA16_04600* | Right-handed parallel beta-helix repeat-containing protein |
| *ACXA16_04605* | EpsG family protein |
| *ACXA16_04610* | Glycosyltransferase family 2 protein |
| *ACXA16_04615* | Polysaccharide biosynthesis protein |
| *ACXA16_04625* | Four helix bundle protein |
| *ACXA16_04720* | SusD/RagB family nutrient-binding outer membrane lipoprotein |
| *ACXA16_04725* | SusC/RagA family TonB-linked outer membrane protein |
| *ACXA16_05020* | Hypothetical protein |
| *ACXA16_05030* | Hypothetical protein |
| *ACXA16_05035* | Carboxypeptidase-like regulatory domain-containing protein |
| *ACXA16_05070* | HU family DNA-binding protein |
| *ACXA16_05180* | Glycosyltransferase family 2 protein |
| *ACXA16_05185* | Glycosyltransferase |
| *ACXA16_05700* | Hypothetical protein |
| *ACXA16_05705* | Hypothetical protein |
| *ACXA16_05710* | Hypothetical protein |
| *ACXA16_05725* | Hypothetical protein |
| *ACXA16_05730* | GTP cyclohydrolase |
| *ACXA16_05770* | Glycosyltransferase |
| *ACXA16_05775* | Hypothetical protein |
| *ACXA16_06220* | IS982-like element ISRa1 family transposase |
| *ACXA16_06245* | IS982-like element ISRa1 family transposase |
| *ACXA16_06350* | Uma2 family endonuclease |
| *ACXA16_06445* | TonB-dependent receptor plug domain-containing protein |
| *ACXA16_06450* | Fic/DOC family N-terminal domain-containing protein |
| *ACXA16_06620* | Carboxypeptidase-like regulatory domain-containing protein |
| *ACXA16_06625* | Glucosaminidase domain-containing protein |
| *ACXA16_06630* | Hypothetical protein |
| *ACXA16_06635* | MBOAT family O-acyltransferase |
| *ACXA16_06640* | GDSL-type esterase/lipase family protein |
| *ACXA16_06780* | ATP-binding cassette domain-containing protein |
| *ACXA16_06790* | IS982-like element ISRa1 family transposase |
| *ACXA16_06955* | DUF2809 domain-containing protein |
| *ACXA16_06960* | Hypothetical protein |
| *ACXA16_07060* | IS1 family transposase |
| *ACXA16_07175* | DNA alkylation repair protein |
| *ACXA16_07190* | Sulfurtransferase |
| *ACXA16_07425* | Hypothetical protein |
| *ACXA16_07600* | DUF6261 family protein |
| *ACXA16_07625* | Cyclic nucleotide-binding domain-containing protein |
| *ACXA16_07630* | Hypothetical protein |
| *ACXA16_07635* | GLPGLI family protein |
| *ACXA16_07640* | Hypothetical protein |
| *ACXA16_07690* | Hypothetical protein |
| *ACXA16_07695* | Hypothetical protein |
| *ACXA16_07715* | Hypothetical protein |
| *ACXA16_07885* | Hypothetical protein |
| *ACXA16_07975* | ABC transporter ATP-binding protein |
| *ACXA16_07985* | Glycosyltransferase family 2 protein |
| *ACXA16_07990* | Capsular polysaccharide synthesis protein |
| *ACXA16_07995* | Glycosyltransferase family 4 protein |
| *ACXA16_08000* | Hypothetical protein |
| *ACXA16_08010* | Acyltransferase |
| *ACXA16_08015* | Hypothetical protein |
| *ACXA16_08020* | Hypothetical protein |
| *ACXA16_08025* | Glycosyltransferase family 2 protein |
| *ACXA16_08130* | Hypothetical protein |
| *ACXA16_08135* | EcoRI family type II restriction endonuclease |
| *ACXA16_08140* | Adenine-specific methyltransferase EcoRI family protein |
| *ACXA16_08180* | IS982-like element ISRa1 family transposase |
| *ACXA16_08270* | ATP-binding cassette domain-containing protein |
| *ACXA16_08425* | Hypothetical protein |
| *ACXA16_08530* | Hypothetical protein |
| *ACXA16_08535* | IS982-like element ISRa1 family transposase |
| *ACXA16_08690* | Hypothetical protein |
| *ACXA16_08830* | Hypothetical protein |
| *ACXA16_08835* | Hypothetical protein |
| *ACXA16_08850* | M12 family metallo-peptidase |
| *ACXA16_08890* | Hypothetical protein |
| *ACXA16_08940* | Hypothetical protein |
| *ACXA16_09320* | TonB-dependent receptor plug domain-containing protein |
| *ACXA16_09385* | Hypothetical protein |
| *ACXA16_09540* | Restriction endonuclease subunit S |
| *ACXA16_09545* | Hypothetical protein |
| *ACXA16_09550* | DNA damage-inducible protein D |
| *ACXA16_09665* | PepSY domain-containing protein |
| *ACXA16_09670* | HU family DNA-binding protein |
| *ACXA16_09675* | Hypothetical protein |
| *ACXA16_09765* | Outer membrane beta-barrel family protein |
| *ACXA16_09770* | Sensor histidine kinase |
| *ACXA16_09775* | LytTR family DNA-binding domain-containing protein |
| *ACXA16_09825* | Hypothetical protein |
| *ACXA16_09840* | Glycoside hydrolase family 2 TIM barrel-domain containing protein |
| *ACXA16_09845* | Carbohydrate binding domain-containing protein |
| *ACXA16_09850* | Glycoside hydrolase family 16 protein |
| *ACXA16_09855* | Hypothetical protein |
| *ACXA16_09860* | Glycosyl hydrolase family 17 |
| *ACXA16_09865* | Glycoside hydrolase family 30 protein |
| *ACXA16_09870* | MFS transporter |
| *ACXA16_09875* | Glycosyl hydrolase family 17 protein |
| *ACXA16_09880* | Carbohydrate binding domain-containing protein |
| *ACXA16_09885* | Triple tyrosine motif-containing protein |
| *ACXA16_09905* | Hypothetical protein |
| *ACXA16_09920* | Hypothetical protein |
| *ACXA16_09925* | Hypothetical protein |
| *ACXA16_09930* | Phosphotransferase |
| *ACXA16_09935* | ABC-F family ATP-binding cassette domain-containing protein |
| *ACXA16_09940* | DUF6934 family protein |
| *ACXA16_09945* | Hypothetical protein |
| *ACXA16_09955* | DUF6934 family protein |
| *ACXA16_09960* | Hypothetical protein |
| *ACXA16_09975* | Chloramphenicol/florfenicol efflux MFS transporter FloR |
| *ACXA16_09990* | Aminoglycoside 6-adenylyltransferase |
| *ACXA16_10015* | DUF6364 family protein |
| *ACXA16_10020* | PIN domain-containing protein |
| *ACXA16_10025* | DUF4184 family protein |
| *ACXA16_10030* | Hypothetical protein |
| *ACXA16_10035* | Cation diffusion facilitator family transporter |
| *ACXA16_10440* | Hypothetical protein |
| *ACXA16_10055* | DUF2971 domain-containing protein |
| *ACXA16_10060* | Hypothetical protein |
| *ACXA16_10065* | Hypothetical protein |
| *ACXA16_10070* | IS1 family transposase |
| *ACXA16_10085* | GNAT family N-acetyltransferase |
| *ACXA16_10090* | Type II toxin-antitoxin system RelE/ParE family toxin |
| *ACXA16_10095* | Helix-turn-helix transcriptional regulator |
| *ACXA16_10100* | NIF family HAD-type phosphatase |
| *ACXA16_10105* | Phosphoribosyltransferase |
| *ACXA16_10110* | DNA-processing protein DprA |
| *ACXA16_10115* | Hypothetical protein |
| *ACXA16_10130* | Hypothetical protein |
| *ACXA16_10135* | Nucleotidyltransferase domain-containing protein |
| *ACXA16_10140* | STING domain-containing protein |
| *ACXA16_10155* | GNAT family N-acetyltransferase |
| *ACXA16_10160* | Zeta toxin family protein |
| *ACXA16_10165* | Hypothetical protein |
| *ACXA16_10170* | Hypothetical protein |
| *ACXA16_10175* | Hypothetical protein |
| *ACXA16_10180* | Hypothetical protein |
| *ACXA16_10185* | Hypothetical protein |
| *ACXA16_10190* | Hypothetical protein |
| *ACXA16_10195* | DinB family protein |
| *ACXA16_10200* | Hypothetical protein |
| *ACXA16_10205* | Type II toxin-antitoxin system ParD family antitoxin |
| *ACXA16_10210* | Type II toxin-antitoxin system RelE/ParE family toxin |
| *ACXA16_10215* | YcxB family protein |
| *ACXA16_10220* | DUF6364 family protein |
| *ACXA16_10225* | PIN domain-containing protein |
| *ACXA16_10230* | Hypothetical protein |
| *ACXA16_10235* | Hypothetical protein |
| *ACXA16_10240* | DUF4184 family protein |
| *ACXA16_10245* | Carboxypeptidase-like regulatory domain-containing protein |
| *ACXA16_10250* | Suppressor of fused domain protein |
| *ACXA16_10255* | Type II toxin-antitoxin system RelE/ParE family toxin |
| *ACXA16_10260* | Helix-turn-helix transcriptional regulator |
| *ACXA16_10265* | Alpha/beta fold hydrolase |
| *ACXA16_10270* | ABC transporter permease |
| *ACXA16_10275* | HlyD family efflux transporter periplasmic adaptor subunit |
| *ACXA16_10280* | Biotin/lipoyl-binding protein |
| *ACXA16_10285* | TolC family protein |
| *ACXA16_10290* | MarR family transcriptional regulator |
| *ACXA16_10295* | Alpha/beta hydrolase-fold protein |
| *ACXA16_10300* | DinB family protein |
| *ACXA16_10305* | Hypothetical protein |
| *ACXA16_10315* | Aspartyl protease family protein |
| *ACXA16_10320* | CPBP family intramembrane glutamic endopeptidase |
| *ACXA16_10330* | Effector binding domain-containing protein |
| *ACXA16_10335* | Hypothetical protein |
| *ACXA16_10340* | Antitoxin VbhA family protein |
| *ACXA16_10345* | Fic family protein |
| *ACXA16_10350* | Aspartyl protease family protein |
| *ACXA16_10355* | CPBP family intramembrane glutamic endopeptidase |
| *ACXA16_10360* | Hypothetical protein |
| *ACXA16_10365* | Hypothetical protein |
| *ACXA16_10370* | DinB family protein |
| *ACXA16_10375* | GLPGLI family protein |
| *ACXA16_10380* | DinB family protein |
| *ACXA16_10385* | GLPGLI family protein |
| *ACXA16_10390* | Hypothetical protein |
| *ACXA16_10395* | Hypothetical protein |
| *ACXA16_10400* | AAA family ATPase |
| *ACXA16_10405* | Type II toxin-antitoxin system RelE/ParE family toxin |
| *ACXA16_10410* | Helix-turn-helix transcriptional regulator |
| *ACXA16_10415* | Giding motility protein GldB-related protein |
| *ACXA16_10420* | Hypothetical protein |
| *ACXA16_10425* | Hypothetical protein |
| *ACXA16_10430* | Hypothetical protein |
| *ACXA16_10435* | Hypothetical protein |
| *ACXA16_10440* | DapH/DapD/GlmU-related protein |
| *ACXA16_10445* | Hypothetical protein |
| *ACXA16_10450* | Hypothetical protein |
| *ACXA16_10455* | Restriction endonuclease |
| *ACXA16_10460* | Hypothetical protein |
| *ACXA16_10465* | Nucleotidyltransferase domain-containing protein |
| *ACXA16_10470* | STING domain-containing protein |
| *ACXA16_10490* | VOC family protein |
| *ACXA16_10510* | Hypothetical protein |
| *ACXA16_10735* | Hypothetical protein |
| *ACXA16_10740* | Fibronectin type III domain-containing protein |
| *ACXA16_10780* | Hypothetical protein |

^a^Specific genes in RA-FJ-C1 strain, but not in RA-FJ-C4 and RA-BJ-C1 strains.

**Table S14** **RA-FJ-C4 strain-specific genes**

| **Gene ID^a^** | **Gene Description** |
| --- | --- |
| *ACXA17_00115* | SusC/RagA family TonB-linked outer membrane protein |
| *ACXA17_00455* | CPBP family glutamic-type intramembrane protease |
| *ACXA17_00600* | IS1595-like element ISRan1 family transposase |
| *ACXA17_00745* | Hypothetical protein |
| *ACXA17_00905* | Hypothetical protein |
| *ACXA17_00925* | Hypothetical protein |
| *ACXA17_00930* | Hypothetical protein |
| *ACXA17_00935* | ATP-binding protein |
| *ACXA17_00940* | Hypothetical protein |
| *ACXA17_00955* | Hypothetical protein |
| *ACXA17_00960* | Hypothetical protein |
| *ACXA17_00970* | DUF3164 family protein |
| *ACXA17_00980* | Hypothetical protein |
| *ACXA17_00990* | Hypothetical protein |
| *ACXA17_00995* | Hypothetical protein |
| *ACXA17_01000* | Hypothetical protein |
| *ACXA17_01005* | Hypothetical protein |
| *ACXA17_01015* | Hypothetical protein |
| *ACXA17_01020* | Hypothetical protein |
| *ACXA17_01025* | Phage minor head protein |
| *ACXA17_01030* | DUF935 family protein |
| *ACXA17_01035* | Hypothetical protein |
| *ACXA17_01040* | Hypothetical protein |
| *ACXA17_01045* | Hypothetical protein |
| *ACXA17_01050* | Hypothetical protein |
| *ACXA17_01055* | Phage protease |
| *ACXA17_01060* | Hypothetical protein |
| *ACXA17_01065* | N-acetylmuramoyl-L-alanine amidase |
| *ACXA17_01070* | Hypothetical protein |
| *ACXA17_01075* | Hypothetical protein |
| *ACXA17_01080* | DUF2586 family protein |
| *ACXA17_01085* | Hypothetical protein |
| *ACXA17_01090* | Hypothetical protein |
| *ACXA17_01095* | Hypothetical protein |
| *ACXA17_01100* | DUF6046 domain-containing protein |
| *ACXA17_01105* | Hypothetical protein |
| *ACXA17_01110* | Hypothetical protein |
| *ACXA17_01115* | Hypothetical protein |
| *ACXA17_01120* | Hypothetical protein |
| *ACXA17_01130* | Nucleotidyltransferase |
| *ACXA17_01135* | Hypothetical protein |
| *ACXA17_01140* | Phage baseplate protein |
| *ACXA17_01160* | Cell wall anchor protein |
| *ACXA17_01295* | Hypothetical protein |
| *ACXA17_01485* | Site-specific integrase |
| *ACXA17_01490* | Helix-turn-helix domain-containing protein |
| *ACXA17_01495* | Hypothetical protein |
| *ACXA17_01500* | Outer membrane beta-barrel family protein |
| *ACXA17_01505* | Hypothetical protein |
| *ACXA17_01510* | 4Fe-4S cluster-binding domain-containing protein |
| *ACXA17_01765* | SPASM domain-containing protein |
| *ACXA17_01770* | Peptidase domain-containing ABC transporter |
| *ACXA17_01775* | RteC domain-containing protein |
| *ACXA17_01780* | Helix-turn-helix domain-containing protein |
| *ACXA17_01785* | Hypothetical protein |
| *ACXA17_01795* | Hypothetical protein |
| *ACXA17_01800* | AAA family ATPase |
| *ACXA17_01805* | DUF6602 domain-containing protein |
| *ACXA17_01810* | Mov34/MPN/PAD-1 family protein |
| *ACXA17_01815* | ThiF family adenylyltransferase |
| *ACXA17_01820* | Nucleotidyltransferase |
| *ACXA17_01825* | XRE family transcriptional regulator |
| *ACXA17_01830* | Hypothetical protein |
| *ACXA17_01835* | DUF6943 family protein |
| *ACXA17_01840* | Hypothetical protein |
| *ACXA17_01845* | DUF5675 family protein |
| *ACXA17_01850* | Hypothetical protein |
| *ACXA17_02940* | Hypothetical protein |
| *ACXA17_03550* | MutS N-terminal domain-containing protein |
| *ACXA17_03555* | RNA-directed DNA polymerase |
| *ACXA17_03560* | Four helix bundle protein |
| *ACXA17_03565* | Hypothetical protein |
| *ACXA17_03575* | Rhodanese-like domain-containing protein |
| *ACXA17_03585* | Sulfite exporter TauE/SafE family protein |
| *ACXA17_03600* | Hypothetical protein |
| *ACXA17_03665* | Endonuclease |
| *ACXA17_04755* | Hypothetical protein |
| *ACXA17_04760* | dTDP-4-amino-4,6-dideoxygalactose transaminase |
| *ACXA17_04765* | GNAT family N-acetyltransferase |
| *ACXA17_04770* | Glycosyltransferase family 2 protein |
| *ACXA17_04805* | O-antigen ligase family protein |
| *ACXA17_04810* | Glycosyltransferase family 4 protein |
| *ACXA17_04815* | Glycosyltransferase family 4 protein |
| *ACXA17_04820* | Hypothetical protein |
| *ACXA17_04825* | O-antigen ligase family protein |
| *ACXA17_04830* | Glycosyltransferase |
| *ACXA17_04835* | Oligosaccharide flippase family protein |
| *ACXA17_04840* | Sugar nucleotide-binding protein |
| *ACXA17_04845* | Polysaccharide biosynthesis protein |
| *ACXA17_04850* | UDP-N-acetyl-D-mannosamine dehydrogenase |
| *ACXA17_05770* | T9SS type A sorting domain-containing protein |
| *ACXA17_06600* | SusC/RagA family TonB-linked outer membrane protein |
| *ACXA17_06605* | RagB/SusD family nutrient uptake outer membrane protein |
| *ACXA17_06815* | Membrane-binding protein |
| *ACXA17_08105* | Choice of anchor J domain-containing protein |
| *ACXA17_08180* | T9SS type A sorting domain-containing protein |
| *ACXA17_08300* | UDP-galactopyranose mutase |
| *ACXA17_08420* | MFS transporter |
| *ACXA17_08450* | GtrA family protein |
| *ACXA17_08520* | TonB-dependent receptor |
| *ACXA17_08715* | Thrombospondin type 3 repeat-containing protein |
| *ACXA17_08720* | Hypothetical protein |
| *ACXA17_08725* | Hypothetical protein |
| *ACXA17_09275* | KUP/HAK/KT family potassium transporter |
| *ACXA17_09320* | Hypothetical protein |
| *ACXA17_09755* | Site-specific integrase |
| *ACXA17_09760* | RhuM protein |
| *ACXA17_09775* | Helix-turn-helix domain-containing protein |
| *ACXA17_09785* | DUF3945 domain-containing protein |
| *ACXA17_09790* | Type IA DNA topoisomerase |
| *ACXA17_09795* | Protein of unknown function (DUF1896) |
| *ACXA17_09800* | N-6 DNA methylase |
| *ACXA17_09805* | Putative translation elongation factor G |
| *ACXA17_09810* | ATP-binding protein |
| *ACXA17_09815* | MULTISPECIES: sigma-54 dependent transcriptional regulator |
| *ACXA17_09820* | Dihydrofolate reductase family protein |
| *ACXA17_09825* | RteC protein |
| *ACXA17_09830* | Antitoxin SocA domain-containing protein |
| *ACXA17_09835* | Hypothetical protein |
| *ACXA17_09840* | Conjugal transfer protein MobC |
| *ACXA17_09845* | Relaxase/mobilization nuclease domain-containing protein |
| *ACXA17_09850* | Conjugal transfer protein MobA |
| *ACXA17_09855* | ParA family protein |
| *ACXA17_09860* | DUF3408 domain-containing protein |
| *ACXA17_09865* | DUF3408 domain-containing protein |
| *ACXA17_09870* | Conjugal transfer protein TraD |
| *ACXA17_09875* | DUF4134 domain-containing protein |
| *ACXA17_09885* | TraG family conjugative transposon ATPase |
| *ACXA17_09890* | DUF3876 domain-containing protein |
| *ACXA17_09895* | DUF4141 domain-containing protein |
| *ACXA17_09900* | Conjugative transposon protein TraJ |
| *ACXA17_09905* | Conjugative transposon protein TraK |
| *ACXA17_09915* | Conjugative transposon protein TraM |
| *ACXA17_09920* | Conjugative transposon protein TraN |
| *ACXA17_09925* | Conjugal transfer protein TraO |
| *ACXA17_09930* | Toprim domain-containing protein |
| *ACXA17_09935* | DUF3872 domain-containing protein |
| *ACXA17_09940* | Glycoside hydrolase family protein |
| *ACXA17_09945* | DUF7688 family protein |
| *ACXA17_09950* | Hypothetical protein |
| *ACXA17_09955* | DUF6956 domain-containing protein |
| *ACXA17_09960* | DUF3873 domain-containing protein |
| *ACXA17_09965* | Hypothetical protein |
| *ACXA17_09970* | PcfJ domain-containing protein |
| *ACXA17_09975* | PcfK-like family protein |
| *ACXA17_09980* | Molybdenum ABC transporter ATP-binding protein |
| *ACXA17_10410* | NADPH-dependent FMN reductase |
| *ACXA17_10420* | Subclass B1 metallo-beta-lactamase |
| *ACXA17_10790* | Fibronectin type III domain-containing protein |
| *ACXA17_10800* | Fibronectin type III domain-containing protein |
| *ACXA17_10805* | Hypothetical protein |

^a^Specific genes in RA-FJ-C4 strain, but not in RA-FJ-C1 and RA-BJ-C1 strains.

**Table S15 RA-BJ-C1 strain-specific genes**

| **Gene ID^a^** | **Gene Description** |
| --- | --- |
| *ACXA18_00595* | IS1595 family transposase |
| *ACXA18_00680* | RAD family carbapenem-hydrolyzing class D beta-lactamase |
| *ACXA18_00750* | Hypothetical protein |
| *ACXA18_01085* | Hypothetical protein |
| *ACXA18_02915* | DUF3440 domain-containing protein |
| *ACXA18_02920* | Hypothetical protein |
| *ACXA18_02925* | DEAD/DEAH box helicase |
| *ACXA18_02930* | Hypothetical protein |
| *ACXA18_02935* | Hypothetical protein |
| *ACXA18_02945* | Hypothetical protein |
| *ACXA18_02950* | Hypothetical protein |
| *ACXA18_02960* | Hypothetical protein |
| *ACXA18_02965* | Phage tail spike protein |
| *ACXA18_02970* | Galactose-binding protein |
| *ACXA18_02975* | Hypothetical protein |
| *ACXA18_02980* | Tape measure protein |
| *ACXA18_02985* | Hypothetical protein |
| *ACXA18_02990* | Hypothetical protein |
| *ACXA18_02995* | Hypothetical protein |
| *ACXA18_03000* | Hypothetical protein |
| *ACXA18_03005* | Hypothetical protein |
| *ACXA18_03010* | Hypothetical protein |
| *ACXA18_03015* | DUF6706 family protein |
| *ACXA18_03020* | Major capsid protein |
| *ACXA18_03025* | Hypothetical protein |
| *ACXA18_03030* | Hypothetical protein |
| *ACXA18_03035* | Hypothetical protein |
| *ACXA18_03040* | Hypothetical protein |
| *ACXA18_03045* | Hypothetical protein |
| *ACXA18_03050* | Hypothetical protein |
| *ACXA18_03055* | Phage portal protein |
| *ACXA18_03060* | Phage terminase large subunit |
| *ACXA18_03065* | PhBC6A51 family helix-turn-helix protein |
| *ACXA18_03070* | Phosphoadenosine phosphosulfate reductase family protein |
| *ACXA18_03075* | ParB N-terminal domain-containing protein |
| *ACXA18_03080* | ParB N-terminal domain-containing protein |
| *ACXA18_03090* | Hypothetical protein |
| *ACXA18_03095* | Hypothetical protein |
| *ACXA18_03100* | Hypothetical protein |
| *ACXA18_03110* | Hypothetical protein |
| *ACXA18_03115* | Hypothetical protein |
| *ACXA18_03120* | Hypothetical protein |
| *ACXA18_03125* | MazG-like family protein |
| *ACXA18_03130* | Hypothetical protein |
| *ACXA18_03135* | Hypothetical protein |
| *ACXA18_03140* | Phosphoadenosine phosphosulfate reductase family protein |
| *ACXA18_03145* | Hypothetical protein |
| *ACXA18_03150* | Hypothetical protein |
| *ACXA18_03155* | Hypothetical protein |
| *ACXA18_03160* | Hypothetical protein |
| *ACXA18_03165* | DUF6291 domain-containing protein |
| *ACXA18_03170* | Hypothetical protein |
| *ACXA18_03175* | Hypothetical protein |
| *ACXA18_03180* | PD-(D/E) XK nuclease-like domain-containing protein |
| *ACXA18_03185* | ERF family protein |
| *ACXA18_03190* | Hypothetical protein |
| *ACXA18_03195* | DUF3853 family protein |
| *ACXA18_03200* | Hypothetical protein |
| *ACXA18_03205* | Hypothetical protein |
| *ACXA18_03210* | Hypothetical protein |
| *ACXA18_03215* | Hypothetical protein |
| *ACXA18_03220* | S24 family peptidase |
| *ACXA18_03225* | Type I restriction endonuclease |
| *ACXA18_03230* | Hypothetical protein |
| *ACXA18_03235* | Hypothetical protein |
| *ACXA18_03240* | Hypothetical protein |
| *ACXA18_04305* | Recombinase RecA |
| *ACXA18_03875* | Restriction endonuclease |
| *ACXA18_04135* | Glycosyltransferase family 2 protein |
| *ACXA18_04140* | EpsG family protein |
| *ACXA18_04145* | Hypothetical protein |
| *ACXA18_04150* | Glycosyltransferase |
| *ACXA18_04155* | Glycosyltransferase |
| *ACXA18_06570* | Glycosyltransferase family 2 protein |
| *ACXA18_04165* | Lipopolysaccharide biosynthesis protein |
| *ACXA18_04455* | TonB-dependent receptor plug domain-containing protein |
| *ACXA18_04740* | Hypothetical protein |
| *ACXA18_05125* | TPR repeat-containing protein |
| *ACXA18_05705* | Cell envelope integrity protein TolA |
| *ACXA18_05715* | T9SS type A sorting domain-containing protein |
| *ACXA18_07095* | DNA alkylation repair protein |
| *ACXA18_07450* | Hypothetical protein |
| *ACXA18_07455* | Hypothetical protein |
| *ACXA18_07880* | Glycosyltransferase |
| *ACXA18_08270* | Dienelactone hydrolase family protein |
| *ACXA18_08275* | Hypothetical protein |
| *ACXA18_08280* | Hypothetical protein |
| *ACXA18_08750* | Outer membrane beta-barrel protein |
| *ACXA18_08755* | Outer membrane beta-barrel protein |
| *ACXA18_08760* | T9SS type A sorting domain-containing protein |
| *ACXA18_08765* | SpvB/TcaC N-terminal domain-containing protein |
| *ACXA18_08770* | IS1 family transposase |
| *ACXA18_09290* | Hypothetical protein |
| *ACXA18_09295* | Hypothetical protein |
| *ACXA18_09480* | Hypothetical protein |
| *ACXA18_09485* | N-acetylmuramoyl-L-alanine amidase |
| *ACXA18_09500* | Hypothetical protein |
| *ACXA18_09505* | Hypothetical protein |
| *ACXA18_09510* | Hypothetical protein |
| *ACXA18_09515* | Nucleotidyltransferase |
| *ACXA18_09525* | Hypothetical protein |
| *ACXA18_09530* | Hypothetical protein |
| *ACXA18_09535* | Hypothetical protein |
| *ACXA18_09540* | Hypothetical protein |
| *ACXA18_09545* | DUF6046 domain-containing protein |
| *ACXA18_09550* | Phage tail tape measure protein |
| *ACXA18_09555* | Hypothetical protein |
| *ACXA18_09560* | Hypothetical protein |
| *ACXA18_09565* | DUF2586 family protein |
| *ACXA18_09570* | Hypothetical protein |
| *ACXA18_09575* | Phage major capsid protein |
| *ACXA18_09580* | HK97 family phage prohead protease |
| *ACXA18_09585* | Phage terminase small subunit-related protein |
| *ACXA18_09590* | Phage terminase large subunit |
| *ACXA18_09595* | Phage protein Gp36 family protein |
| *ACXA18_09600* | Hypothetical protein |
| *ACXA18_09605* | DUF935 family protein |
| *ACXA18_09610* | Phage minor head protein |
| *ACXA18_09615* | Hypothetical protein |
| *ACXA18_09620* | Hypothetical protein |
| *ACXA18_09625* | Hypothetical protein |
| *ACXA18_09630* | Hypothetical protein |
| *ACXA18_09635* | Hypothetical protein |
| *ACXA18_09640* | Hypothetical protein |
| *ACXA18_09680* | DUF3164 family protein |
| *ACXA18_09690* | Hypothetical protein |
| *ACXA18_09705* | AAA family ATPase |
| *ACXA18_09710* | ATP-binding protein |
| *ACXA18_09715* | Hypothetical protein |
| *ACXA18_09720* | Hypothetical protein |
| *ACXA18_09725* | Hypothetical protein |
| *ACXA18_09750* | Helix-turn-helix domain-containing protein |
| *ACXA18_09755* | Alkaline phosphatase |
| *ACXA18_10065* | MarR family transcriptional regulator |
| *ACXA18_10070* | NAD (+) --rifampin ADP-ribosyltransferase |
| *ACXA18_10075* | Toxin-antitoxin system YwqK family antitoxin |
| *ACXA18_10085* | EreD family erythromycin esterase |
| *ACXA18_10090* | DUF4932 domain-containing protein |
| *ACXA18_10100* | Hypothetical protein |
| *ACXA18_10105* | Hypothetical protein |
| *ACXA18_10115* | S41 family peptidase |
| *ACXA18_10120* | GTPase |
| *ACXA18_10140* | WG repeat-containing protein |
| *ACXA18_10145* | DUF5694 domain-containing protein |
| *ACXA18_10150* | Hypothetical protein |
| *ACXA18_10405* | Hypothetical protein |
| *ACXA18_10410* | Hypothetical protein |
| *ACXA18_10420* | Hypothetical protein |
| *ACXA18_10760* | DNA-binding domain-containing protein |

^a^Specific genes in RA-BJ-C1 strain, but not in RA-FJ-C1 and RA-FJ-C4 strains.
